# Optimizing reversible solid oxide cell technology for long-term energy storage in Europe’s decarbonized frameworks

**DOI:** 10.1038/s41467-026-74229-z

**Published:** 2026-06-25

**Authors:** Xinyi Wei, Arthur Waeber, Shivom Sharma, Jan Van herle, Francois Maréchal

**Affiliations:** 1 Industrial Process and Energy Systems Engineering-École Polytechnique Fédérale de Lausanne (EPFL), Sion, Switzerland; 2 Group of Energy Materials-École Polytechnique Fédérale de Lausanne (EPFL), Sion, Switzerland

**Keywords:** Energy modelling, Electrical and electronic engineering, Batteries

## Abstract

Energy storage is crucial for enhancing energy security. In this study, we explore the indispensable roles of both long-term and short-term energy storage solutions, demonstrating their combined benefits. By achieving 60% round-trip efficiency of long-term storage through reversible solid oxide cells (rSOC) technology, we affirm rSOC’s immediate environmental and economic advantages and challenge its perception as merely a future-oriented technology within the current European energy framework. Looking forward to Europe’s 2050 energy landscape, our findings indicate substantial variability in storage capacity requirements for various countries, ranging from 54 to 680 kWh/kW for the proposed rSOC system. The use of rSOC as a unique storage technology achieves a minimum levelized cost of storage (LCOS) of 0.88 €/kWh, whereas battery systems reach a lower minimum value of 0.67 €/kWh but exhibit a much broader cost range depending on country and energy context. We demonstrate that hybrid strategies emerge as the most economically viable, reducing costs by up to 78% and, in some cases, to as low as 0.19 €/kWh. These findings support hybrid energy storage strategies as a future-oriented solution.

## Abstract (web summary)

The study shows that optimized reversible solid oxide cells (rSOC) can store renewable energy over long periods with about 60% round-trip efficiency. Storage needs vary widely across Europe (54-680 kWh/kW); rSOC-battery hybrid systems are the most economical, cutting costs by up to 78%.

## Introduction

Fossil fuels significantly impact the environment and climate through the release of greenhouse gases and air pollutants, making the transition to renewable energy sources essential for achieving the environmentally target^1,2^. Renewable energy sources, such as wind, solar, and hydropower, can provide cleaner energy and enhance energy security by diversifying supply sources^3^. However, the integration of intermittent renewables into electrical grids poses challenges. As their share in the energy mix grows, their variability becomes increasingly important; therefore, a reliable energy supply requires robust storage strategies^4^. High-capacity storage solutions are needed to balance supply and demand, ensuring the reliability of energy systems^5^.

By 2030, lithium-ion batteries are expected to be the most cost-effective short-term storage solution, typically covering durations of up to a few days^6^. Yet, the need for long-term storage, which extends to weeks or even seasonal scales, will also grow, emphasizing strategic planning for continuous energy supply during low-generation periods^7–9^. Long-term storage is therefore crucial for achieving zero-carbon electricity, maintaining grid stability, and ensuring cost-effectiveness^10^. Reversible solid oxide cell (rSOC) is a promising technology for long-term energy storage, enabling low-carbon energy services for future decarbonized systems^11^. An rSOC system can be designed to store excess renewable electricity by converting it to an energy carrier, such as hydrogen or other fuels that can easily be stored and subsequently converted back into electricity during supply shortages. This technology offers a geographically flexible storage solution, independent of site-specific features^12^. Despite high initial costs, increased rSOC production rates can mitigate these, making rSOC suitable for long-term energy storage strategies^13^. Several studies and demonstration projects have investigated rSOC systems for renewable energy integration and long-term energy storage. Reported systems demonstrate round-trip efficiencies typically ranging from 50 to 70%, with applications including power-to-X processes, synthetic fuel production, and seasonal energy storage^14–20^. Additional discussion of representative studies and key performance considerations is provided in the Supplementary Information (SI Method S0). Interestingly, in 2024, a pioneering rSOC system (25 kW fuel cell/75 kW electrolyzer) was demonstrated in the Netherlands^21,22^, and it was operated continuously in both modes ( > 500 h). While not configured for large-scale energy storage, this demonstrator validated the technical feasibility and dual-mode operability of rSOC systems, reinforcing their potential relevance in sustainable energy systems from both environmental and economic perspectives. Despite ongoing research and development, critical gaps remain in integrating key metrics, such as efficiency, waste heat valorization, and normalized storage capacity, into a realistic energy system framework.

In this study, we optimized the rSOC system design as a fundamental step toward improving long-term storage performance and enabling seamless integration into existing energy systems^23^. Key features include atmospheric pressure operation, ejector integration, and a hybrid storage tank that can store both methane for use in the fuel cell and CO_2_ for methane synthesis using hydrogen produced from the electrolyzer^24^. Building on this foundation, we explored the effective integration of rSOC systems into broader energy frameworks. Although their immediate economic benefits in the current systems may be limited, the improved environmental performance and scalability of rSOC systems justify a detailed evaluation. We investigated rSOC implementation in scenarios with a modest renewable energy share, shedding light on its role in ongoing energy transitions. Looking ahead to 2050, we developed country-specific guidelines for energy storage based on projected electricity generation profiles across Europe. By integrating rSOC with complementary technologies, such as batteries, the study identifies optimal strategies that balance short- and long-term storage needs. Additionally, we provide standardized parametric values for storage sizes and the levelized cost of storage across Europe, supporting the development of customized strategies that leverage the unique benefits of diverse energy storage technologies.

## Results

### Long-term storage performance analysis of the rSOC system

This section evaluates the key system employed within the energy storage framework, namely the rSOC system. The system operates in solid oxide electrolyzer (SOEC) mode during periods of excess or low-cost, typically renewable, electricity to synthesize fuel, and switches to solid oxide fuel cell (SOFC) mode when there is a demand for electricity. Overall, the proposed advanced rSOC system (as presented in the Methods section: rSOC system modeling for energy conversion and storage) demonstrates improved performance in both SOEC and SOFC modes, achieved through advanced features, such as using a two-ejector and a hybrid storage tank, instead of the typical one-ejector/two tanks setup^25–28^. Moreover, due to its high-temperature operation feature, rSOC system generates heat as a by-product. This heat can be converted into electricity via a Rankine cycle (RC) to improve system efficiency or used in industrial processes and district heating networks. In this study, the RC is integrated to enhance efficiency and assess waste heat valorization potential.

In the conventional SOEC-methanator systems, the methanator typically operates at high pressure (15 bar) to ensure high conversion efficiency, which in turn necessitates multiple compressors and heat exchangers prior to the fuel synthesis step. In this study, the proposed SOEC configuration not only eliminates the need for multi-stage heating and a mechanical expansion device for  the CO_2_ flow, but also removes one stage of compression and cooling for the hydrogen flow. As a result, the proposed SOEC system reaches a conversion efficiency of 82.1% with RC integration, surpassing the 78.6% efficiency of traditional designs (Supplementary Table B1 in the SI, Methods section: Advanced SOEC system design and modeling for process description). By adopting this system design, it significantly reduces compressor electricity consumption to 0.597 kW, compared to 1.905 kW in the traditional design, primarily due to the use of an ejector that utilizes the high pressure of the hybrid storage tank to replace mechanical compression. Moreover, the proposed system requires eighteen heat exchangers (HEX) with a total area of 0.465 m^2^, compared to twenty HEX units and 0.431 m^2^ total estimated area. In addition, due to improved thermal integration, the cold utility demand (typically provided by cooling water) used to remove the remaining excess heat after internal heat recovery is reduced to 3.318 kW, significantly lower than 4.11 kW for the traditional design. These results indicates the efficiency gains and operational advantages of the proposed atmospheric-pressure SOEC system, enabled by ejectors and advanced thermal management. Overall, the integration of ejectors and a high-pressure hybrid storage tank in the proposed design reduces reliance on these auxiliary components, minimizes energy losses, effectively utilizes the pressure of the storage tank, and lowers cold utility demand.

Current research and industrial efforts on SOFC systems focus on improving efficiency, simplifying system layout, and reducing dependence on external water sources, which can be costly when purification is required. One potential approach is the use of low- to high-temperature anode off-gas for internal steam recirculation; however, this often introduces additional system complexity. In this context, this study addresses these challenges and evaluates the proposed design against conventional configurations. Furthermore, in SOEC mode, the same stack operates at approximately twice the voltage and 1.5 times the current relative to SOFC mode. As a result, the SOFC power output is roughly one-third of the SOEC power input.

In SOFC mode, the proposed system is compared with No anode off-gas (AOG) and Warm AOG, and a third, less commonly used configuration, Hot AOG (Supplementary Table A2 in SI for design specifications, Supplementary Fig. A2 in SI for process layout; see section “Method-Advanced SOFC system design and modeling” for process description). The proposed system achieves an electrical efficiency of 72.6% with RC integration, which exceeds the efficiencies of 71.3%, 71%, and 65.9% for No AOG, Warm AOG, and Hot AOG designs, respectively. This improvement results from the AOG recirculation strategy, which eliminates the need for a compressor while allowing unconverted fuel to be recirculated and reused within the system, thereby improving overall fuel utilization compared to the No AOG configuration. However, although the Warm AOG and Hot AOG designs also benefit from AOG recirculation, the requirement for a blower becomes a limiting factor, reducing the overall system efficiency. In addition, the system streamlines its operation by eliminating external water use through AOG recirculation. The proposed SOFC system requires nine HEX units with a total area of 0.142 m^2^, compared to twenty-one HEX units and 0.425 m^2^ total area in the no-AOG design. The cold utility demand is also lowered to 5.28 kW, compared to 7.33 kW in the no-AOG design (Supplementary Table B3 and Supplementary Fig. B1 in SI). This reduction results from the use of the ejectors, which enable direct mixing of streams and improved heat utilization, thereby decreasing the remaining heat that must be removed by external cooling.

Overall, the proposed atmospheric-pressure SOFC system outperforms both traditional and emerging designs. The 10 kW system, validated against experimental data, serves as a reference model, with results scalable to larger systems. Although ejector integration poses design challenges due to system-specific requirements, promising demonstrations by Cummins and AIST (Japan) highlight its feasibility. With further development, ejectors could be standardized for mass production, supporting their broader deployment. Detailed material and energy flows and system comparisons are provided in Supplementary Results S1 and S2, Supplementary Tables B2 and B4 in SI for a more comprehensive understanding.

Several insights from this section warrant further discussion. Previous studies have demonstrated the feasibility of integrating a small-scale RC into rSOC systems for effective waste heat valorization. This study assesses a 10/30 kW SOFC/SOEC system, representative of the commercial single-stack scale, and highlights potential efficiency gains from RC integration, particularly for larger systems where a centralized market-available RC unit can recover heat from multiple stacks. Although SOEC operation requires an external high-temperature heat source to reach the required input-stream temperatures, optimal heat integration enables the system to recover medium-temperature waste heat, primarily from the exothermic methanation step, which can be utilized for district heating or industrial applications (Supplementary Fig. B2 in SI). Moreover, the rSOC system still requires a moderate number of heat exchangers even after heat integration, but more than 80% of those used in SOFC mode can be reused in SOEC mode^29^, offering potential reduction in system footprint and capital cost. At larger scales, this complexity can be further addressed by packaging the heat exchangers into standardized modules, minimizing customization and improving practical deployment.

Overall, unlike cost or environmental impact, which can be influenced by region-specific energy mixes and economies of scale, system efficiency is inherently limited by technological design. This section, therefore, focuses on optimizing system performance, laying a solid foundation for integrating rSOC into long-term energy storage systems. In addition, electrical conversion losses associated with alternating-current/direct-current (AC/DC) interfacing between the stack and the grid are not considered at this stage, but are included later in the energy system integration analysis. Nonetheless, broader challenges, such as environmental emissions, transient conditions, control complexity during mode switching, and operational stability, are linked to rSOC technology in general and lie beyond the scope of this system-level optimization. Addressing these issues will require further dedicated studies on dynamic operation and advanced control strategies to complement the proposed design improvements.

### Current energy framework: rSOC system integration for long-term storage

Building on the previously optimized rSOC system design, we examine the integration of the rSOC technology and the required storage capacity within Europe to evaluate its economic viability and environmental impact. The Inputs from the presented system are the charging (SOEC) and discharging (SOFC) efficiencies and the nominal storage conditions. We specifically focus on a 50 kW SOFC/150 kW SOEC system as an illustrative example. The strategy exploits periods of low electricity prices or low carbon intensity to operate the SOEC and convert electricity into fuel stored in the hybrid tank. Conversely, periods of high electricity prices or high carbon intensity trigger SOFC operation, generating electricity from the stored fuel. The analysis evaluates the trade-offs between environmental impacts (CO_2_ emissions) and economic performance (operational costs) under current grid conditions, using a reference case without rSOC deployment for comparison, which assumes no associated environmental or economic benefits. Part-load operation is neglected, as the system is assumed to operate either at full load in one mode (SOEC or SOFC, depending on grid conditions) or in hot standby when grid conditions are not favorable for either mode. Switzerland, with its partially decarbonized electricity production and significant winter imports, serves as a case study due to its notable variations in electricity price and carbon intensity. While Switzerland is used as a representative example to explain the findings, the outcome offers general guidelines for rSOC operation and switching strategies, providing insights into its market potential in the current energy framework.

A multi-period optimization problem was formulated using a typical-day clustering method to effectively and accurately represent country-level hourly profiles, as explained in the Methods. In general, the optimization revealed that an intelligent operation of the proposed rSOC system achieves a maximum annual CO_2_ savings of approximately 19 tons by frequent mode switching, corresponding to the annual inland CO_2_ emissions of 4–5 persons in Switzerland^30^.

**Fig. 1:**
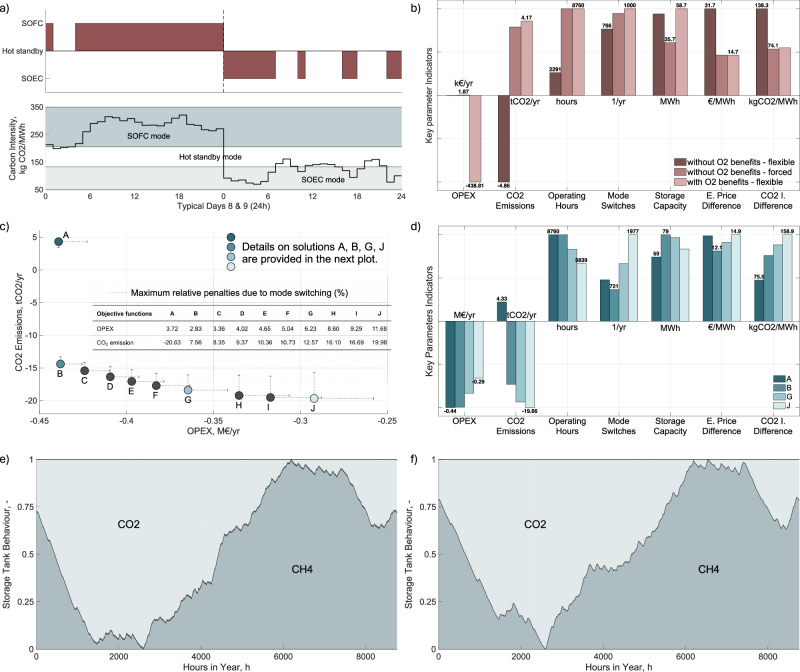
Integration of the reversible solid oxide cell (rSOC) system into Switzerland’s current energy framework. **a** rSOC operating strategy focusing on CO_2_ emissions, responding to varying carbon intensity profiles (in kg CO_2_/MWh); **b** Operational comparisons of the rSOC system under forced and flexible conditions, with and without the sale of O_2_; **c** Pareto-optimal front showing trade-offs between CO_2_ emissions and operational expenditures (OPEX), including uncertainties from mode switching (in %); **d** Operational results for solutions A, B, G, J from the Pareto-optimal front; **e** Annual dynamics of CH_4_/CO_2_ volume ratios in the hybrid tank for Solution A (targeting minimum OPEX); **f** Annual dynamics of CH_4_/CO_2_ volume ratios in the hybrid tank for Solution J (targeting minimum CO_2_ emissions). Source data are provided as a Source Data file.

Figure 1a illustrates the operating strategy for typical days 8 and 9, showing transitions among the three modes: SOFC, SOEC, and hot standby. The hot standby mode maintains the stack at operating temperature without electrochemical activity, enabling rapid mode switching with minimal energy discharge. Its impact is discussed in the following section. These transitions are triggered by significant variations in the carbon intensity of electricity, which serves as an input to the optimization framework. The optimizer determines the mode-switching schedule based on the selected objective function (i.e., minimize CO_2_). For instance, as shown at the end of a typical day 8 (24 h) and the beginning of day 9 (0 h), based on optimization results, when carbon intensity exceeds 200 kg CO_2_/MWh, the system switches to SOFC mode to supply electricity to the grid, while carbon intensity below 130 kg CO_2_/MWh prompts SOEC operation to convert electricity into fuel. For carbon intensity between 130 and 200 kg CO_2_/MWh, the system remains in hot standby mode, as seen between the 7 and 10^th^ hours on a typical day 9. The optimized strategy balances the trade-offs between emission reductions and the system’s round-trip efficiency.

### Operational costs analysis for the rSOC system

Figure 1b summarizes the optimized results for the rSOC system, using OPEX as the objective function. In the first scenario, which excludes economic benefits from oxygen sales, the flexible operation strategy leads to 2291 operating hours compared to the forced operation strategy (8760 h) and achieves negative CO_2_ emissions. However, forcing the system to operate year-round incurs additional costs of 1870 €/year, while the flexible strategy results in a net benefit of 1010 €/year. In the second scenario, incorporating oxygen sales as an economic benefit, the flexible strategy prompts continuous operation throughout the year, with an increased switching frequency of 1000 times. Although this approach maximizes economic returns, it significantly increases CO_2_ emissions due to a smaller difference in carbon intensity between electricity purchased from and injected into the electricity, resulting in net positive emissions.

While the recovery of pure oxygen from the SOEC system presents technological challenges, primarily related to safety concerns, such as the risk of explosion during pure oxygen handling, it has minimal impact on system performance, as discussed in the Methods section. Therefore, the main barrier to implementation is safety rather than technical feasibility. Overcoming these challenges could significantly enhance the market potential of rSOC systems. Accordingly, subsequent analyses in this study consider oxygen as a marketable product, reflecting its potential future value and highlighting a promising direction for further research and development^31^.

### Trade-off solutions analysis for the rSOC system

Although the current analysis focuses on single-objective optimization, exploring trade-offs between CO_2_ emission reductions and economic benefits offers a more comprehensive perspective for identifying optimal operating strategies. Figure 1c illustrates the Pareto-optimal front between CO_2_ emissions and operational costs, revealing a non-linear relationship where higher economic gains correspond to increased CO_2_ emissions compared with the no-electricity-use reference case. This study uses the weighted-sum method to solve a bi-objective optimization problem.

Solutions A and B, for example, achieve similar economic gains but with markedly different environmental outcomes. As detailed in Fig. 1d, Solution A focuses solely on maximizing economic profitability, resulting in a CO_2_ increase of 4.33 t/year. In contrast, Solution B reduces emissions by 14.38 t/year, albeit with fewer system switches (721 vs. 946). This disparity reflects the difference in CO_2_ intensity between buying and selling electricity: 120.61 kg CO_2_/MWh for Solution B compared to 75.48 kg CO_2_/MWh for Solution A. Solutions G and J provide further insights into the trade-offs between CO_2_ reductions and operating costs. To achieve an additional CO_2_ reduction of 1.28 t/year (from −18.38 to −19.66 t/year), there is an increase in operating costs by 0.072 M€/year (from −0.367 to −0.295 M€/year). Solution J, which focuses solely on minimizing CO_2_ emissions, requires a maximum carbon intensity difference between buying and selling electricity (158.92 kg CO_2_/MWh) to offset rSOC system round-trip efficiency losses. These results emphasize the importance of identifying balanced operating strategies, as pursuing extreme outcomes, whether economic or environmental, may lead to substantial compromises in the other objective. Such trade-offs require careful consideration to understand the associated gains and losses and to ensure system performance aligns with broader sustainability or financial goals. More information on all Pareto solutions is provided in Supplementary Table B5 in SI.

The impact of mode switching and hot standby on the accuracy of the results is presented as error bars for the trade-off solutions and is provided in Fig. 1c. The results shows that increasing mode switches to maximize differences in electricity price or CO_2_ intensity leads to greater penalties. OPEX penalties are less affected by mode switching due to the higher income from selling O_2_. Excessive mode switching is not beneficial, as penalties increase linearly with the number of switches. For example, Solution J, which relies on excessive switching for maximal CO_2_ savings, incurs a penalty of 19.98%. Interestingly, Solution A shows a negative penalty for CO_2_ emissions due to reduced operation time, which lowers overall emissions. Overall, Solution B emerges as the most balanced option, with penalties of 2.83% and 7.56% for economic and environmental objectives, respectively. These findings underscore the importance of considering transient impacts when evaluating trade-off solutions.

### Performance analysis of the hybrid tank in the rSOC system

This study investigates a hybrid tank configuration in which fuel and CO_2_ are maintained at high pressure in adjacent compartments. A movable partition adjusts compartment volumes based on the respective quantities of fuel and CO_2,_ as described in the Methods section: rSOC system modeling for energy conversion and storage. The energy storage (hybrid) tank capacity for the rSOC system is influenced by multiple factors, including electricity price difference, operating durations of SOFC and SOEC modes, and CO_2_ emissions. A larger price difference between selling and buying electricity tends to reduce the required storage capacity, as shown in Fig. 1d. A similar trend is visible for the number of switches, where Solution A stands out as an exception. The required storage capacity ranges from 59 to 79 MWh for the proposed rSOC system, resulting in a storage volume of 78 to 104 m^3^ at 75 bar or 27 to 37 m^3^ at 200 bar. Given that commonly available storage tanks range from 20 to 50 m^3^, operating such a 50 kW/150 kW rSOC system would require 2–5 storage units.

Figure 1e, f depict the storage tank behaviors for the two Pareto-optimal solutions: solution A (minimum OPEX) and Solution J (minimum CO_2_ emissions). Regardless of the objective, cyclic trends are evident. Methane is consumed early in the year due to high winter electricity prices and replenished in autumn as lower electricity prices enable methane production. For operational continuity, the CH_4_ to CO_2_ ratio in the storage tank must remain between 2.5 and 3.5 on January 1st, ensuring stable performance throughout the year.

### Future energy framework: rSOC system integration for long- and short-term storage in Europe

As Europe advances toward net-zero emissions by 2050, renewable energy’s intermittent nature will require robust storage solutions for both short- and long-term energy needs. rSOC technology offers a promising long-term option for managing renewable electricity fluctuations and enhancing self-sufficiency. Unlike current integration efforts focused on economic and environmental benefits, the 2050 framework prioritizes optimizing storage capacities to effectively balance surplus and shortage periods. Thus, exploring rSOC’s role in Europe’s future energy framework, emphasizing flexible stack sizing and standardized storage metrics independent of stack size for each European country, is also interesting. Mode-switching impacts are excluded, as future energy profiles inherently account for significant uncertainties, though insights from previous analyses remain applicable. The study also considers rSOC technology alongside traditional storage systems such as batteries and pumped-hydro storage, offering guidance on integrated storage strategies tailored to national renewable profiles. Moreover, the energy storage tank sizes are normalized based on the technology’s output power, enabling utility companies to easily scale storage capacities to meet their requirements. Finally, this section also highlights the potential for cost reductions in energy storage across all technologies through integrated storage solutions.

### Performance analysis of the long-term storage system

The decarbonized energy mixes projected for 2050 will vary significantly across European countries, reflecting differences in renewable energy potentials, government policies, technological advancements, and environmental conditions. Building on energy demand and production profiles detailed in Santecchia et al. ^32^, this section analyzes the correlation between renewable energy types and the normalized storage capacity. Rather than quantifying the exact share of renewables, the analysis provides general guidelines for aligning the long-term storage capacities with diverse energy profiles across Europe.

**Fig. 2:**
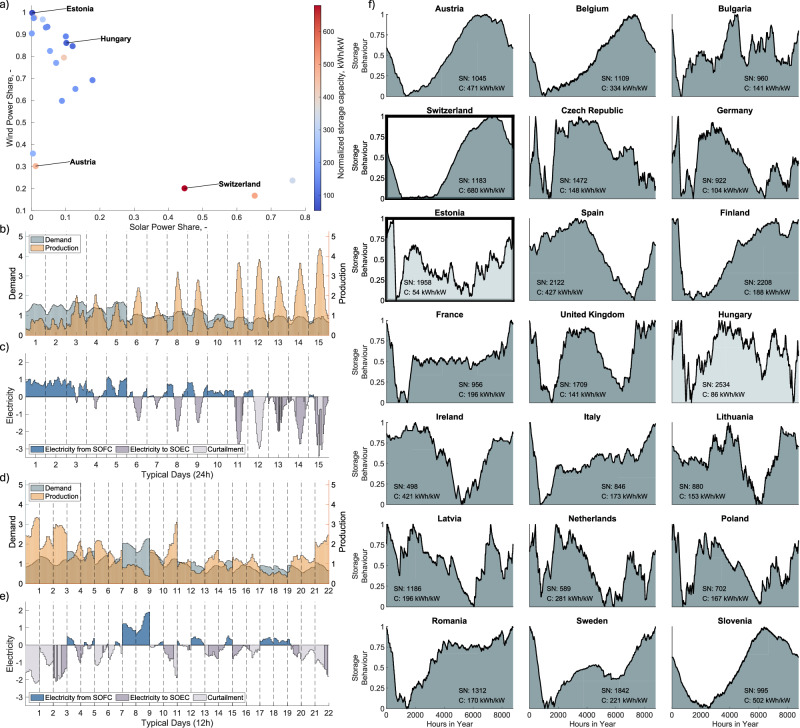
Integration of the reversible solid oxide cell (rSOC) system into future energy systems. **a** Correlation between normalized storage size (in kWh/kW) and renewable energy sources; **b** Renewable electricity production and demand (in kW/kW_avg, demand_) on typical days for Switzerland; **c** Electricity flows in the rSOC system (in kW/kW_avg, demand_) on typical days for Switzerland (long-term storage behavior); **d** Renewable electricity production and demand (in kW/kW_avg, demand_) on typical days for Estonia; **e** Electricity flows in the rSOC system (in kW/kW_avg, demand_) on typical days for Estonia (short-term storage behavior); **f** Comparative storage behaviors across 21 European countries, categorized by long-term-oriented (dark) and short-term-oriented (light) storage behaviors, including the number of switches (SN) and normalized storage capacity (C) in kWh/kW. Source data are provided as a Source Data file.

Figure 2a illustrates the shares of solar and wind power in total renewable energy production across European countries and their impact on normalized storage capacity using the proposed rSOC system. Storage tank capacities vary significantly, ranging from 54 to 680 kWh/kW. Countries with a high share of wind power, such as Estonia (0.99) or Hungary (0.86), require smaller storage capacities, shown in blue, due to the relatively stable nature of wind energy. In contrast, solar power, with its higher variability and seasonal fluctuations, necessitates larger storage capacities. For example, Switzerland, with a solar power share of 0.45, requires the largest storage capacity, indicated in dark red. Austria, relying mainly on hydraulic dams for its electricity production, also shows a high seasonality.

To further analyze the results, Switzerland and Estonia were selected as representative examples due to their contrasting storage requirements, with Switzerland requiring the largest and Estonia the smallest storage tank capacities. Figure 2b, d show the renewable electricity demand (yellow) and production (green) on selected typical days by using the clustering approach, normalized to an average electricity demand of 1 kW, for the two countries, respectively. In this approach, the full year of hourly renewable generation and demand data is statistically grouped into a limited number of representative “typical days,” which preserve the main temporal patterns of the original time series while significantly reducing the computational effort required for system optimization. These typical days are later sequenced and weighted to reconstruct the annual profile, as detailed in the Methods section: Energy system integration. In Switzerland, a significant mismatch between electricity demand and production is observed on most typical days, especially during summer and winter, highlighting the need for long-term storage. In contrast, Estonia exhibits a smaller discrepancy between electricity production and demand relative to the overall pattern across the other countries, reflecting a reduced reliance on extensive energy storage solutions. Figure 2c, e show typical days of electricity production from SOFC and electricity-to-fuel conversion using SOEC for Switzerland and Estonia as examples. When electricity production exceeds demand in Switzerland (e.g., typical day 8 in Fig. 2b), the system operates in SOEC mode, as reflected in the corresponding SOEC activity shown for typical day 8 in Fig. 2c. Conversely, when electricity demand is greater than electricity production (e.g., typical day 1 in Fig. 2b), the system switches to SOFC mode, as illustrated for typical day 1 in Fig. 2c. The same principle applies to Fig. 2d, e for the Estonia case. Although these patterns do not directly correspond to storage tank flows because typical days must be sequenced to represent the complete annual storage profile, interesting patterns emerge for visual comparisons. For instance, in Switzerland, the maximum power supply of the SOFC peaks at 1.15 kW/kW_avg, demand_, compared to 1.90 kW/kW_avg demand_ in Estonia. This emphasizes Estonia’s high peak power needs.

Figure 2f illustrates the yearly storage behaviors across most European countries by sequencing the typical day profiles, detailing the number of mode switches (SN) and normalized storage capacities (C) in kWh/kW. For instance, in Switzerland, the rSOC system undergoes 1183 mode switches and requires a storage tank capacity of 680 kWh/kW. In contrast, the Estonian rSOC system experiences more frequent switching, 1958 times, and necessitates a smaller storage tank capacity of 54 kWh/kW, approximately one-twelfth of Switzerland’s requirement. This reflects Estonia’s reduced need for long-term storage. These storage capacities, normalized to 1 kW power supply, can be scaled for different applications. For example, an industry with an average electricity consumption of 50 kW would need a maximum power supply of 58 and 95 kW and a storage tank capacity of 39 MWh and 5 MWh in Switzerland and Estonia, respectively. This results in storage volume requirements of roughly 51 and 7 m^3^, respectively, at 75 bar. The conclusions drawn are also broadly applicable across Europe. Countries with relatively low shares of wind energy, or significant seasonal fluctuations, require storage durations over 100 hours and can thus be considered as long-term-oriented storage solutions (dark-green). Conversely, countries with higher reliance on wind and negligible seasonal variation face mostly day-to-day fluctuations and benefit from smaller, short-term-oriented storage solutions (light green). Still, most of the countries exhibit mixed behaviors, demonstrating both seasonal trends (rSOC system) and high-power fluctuations (conventional battery), suggesting the need for a hybrid storage strategy, which will be explored further in subsequent section.

### Strategic integration of energy storage technologies

In energy storage, batteries and pumped-hydro storage are established technologies, providing solutions for short-term (hours to days) and long-term (weeks to seasons) energy storage, respectively. Thus, integrating batteries into the optimization framework to evaluate their required sizes for most European countries in 2050, alongside the outcomes for rSOC-based storage, is informative. For pumped-hydro storage, the analysis focuses on countries with favorable geographical conditions, evaluating their role in long-term energy storage. Rather than identifying a single optimal technology, this analysis provides guidance for combined storage strategies tailored to each country’s energy supply and demand.

**Fig. 3:**
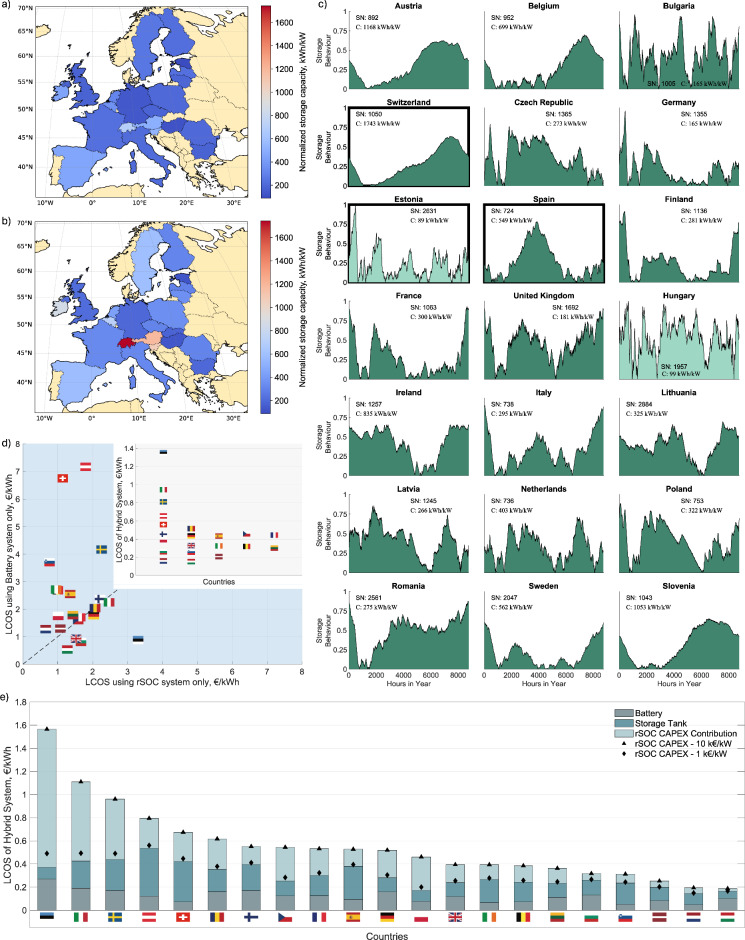
Storage systems behaviors for the future energy mix. **a** Normalized storage capacities (in kWh/kW) of reversible solid oxide cell (rSOC) systems for 21 European countries; **b** Normalized storage capacities (in kWh/kW) of battery systems for 21 European countries; **c** Storage behaviors of the battery systems for 21 European countries, categorized by long-term-oriented (dark) and short-term-oriented (light) storage behaviors; **d** Estimated levelized costs of storage (in €/kWh) for battery, rSOC and hybrid systems for the 21 considered European countries. **e** Sensitivity analysis on the rSOC CAPEX in k€/kW_FC_. Source data are provided as a Source Data file.

Figure 3a, b illustrate the storage capacities per maximum power supply across selected European countries for rSOC and batteries, respectively, highlighting the suitability of each technology based on geographic and energy characteristics. rSOC systems generally require smaller storage capacities, with countries such as Switzerland, Slovenia, and Austria exhibiting normalized storage capacities of 680, 502, and 471 kWh/kW, respectively, compared to 1743, 1053, and 1168 kWh/kW for conventional battery systems. These countries benefit from factors such as Austria’s reliance on hydro reservoirs during summer and Switzerland’s seasonal PV production, making rSOC an ideal choice for long-term storage. Conversely, Estonia, Hungary, and Bulgaria exhibit more short-term-oriented storage strategies, with normalized sizes of 54, 86, and 141 kWh/kW for the rSOC system and 89, 99, and 165 kWh/kW for battery system, respectively, due to the less intermittent nature of their renewable energy sources. In these countries, it may be more practical to consider short-term storage technologies (e.g., slightly oversized batteries) in order to avoid investment in cost-intensive long-term storage solutions. The high energy density of CH_4_ at 75 bar results in normalized storage sizes ranging from 0.07 to 0.89 m^3^/kW for the rSOC system, compared to 0.2 to 3.87 m^3^/kW for the battery system. This contrast highlights both the substantial oversizing required for battery systems in long-term storage application and the critical importance of energy density when evaluating storage volume requirements. Details on rSOC and battery results can be found in Supplementary Tables B6 and B7 in the SI, respectively.

In Fig. 3c, the storage behavior of the modeled battery system is depicted across most European countries. This analysis, while not meant for direct comparison, serves to identify general trends and potential synergies between battery and rSOC storage within complex energy frameworks. While Switzerland and Estonia represent typical long-term and short-term-oriented storage strategies, respectively, most of the countries, such as Spain, would benefit significantly from adopting hybrid storage strategies. Figure 3d further substantiates these findings by incorporating the estimated levelized cost of storage (i.e., energy storage in batteries or molecules, and conversion of stored energy into electricity) for both single-technology and hybrid storage options. The previously discussed countries exhibit a clear cost-based advantage for single-technology storage solutions. For instance, in Switzerland, the levelized cost of storage (LCOS) of the rSOC system is one-sixth that of the battery system, whereas in Estonia, the LCOS of the rSOC system is 3.4 times higher than that of the battery system. Still, the main findings indicate that a hybrid energy storage strategy is advantageous for most countries. Specifically, combining battery storage to manage peak demand with an rSOC system for energy balancing can significantly reduce overall investment costs. The minimum rated power of the rSOC system can be estimated by dividing the total required stored energy by the more restrictive of the full-charge or full-discharge durations. Implementing such a hybrid storage strategy could allow countries such as the Netherlands and Hungary to reduce their LCOS to approximately 18 cents/kWh, representing a potential reduction of up to 75% compared to single-technology storage solutions. Detailed information on the cost calculations and the actual values can be found in Supplementary section Method S4 and in Supplementary Table B8 in SI.

Although this study does not aim to emphasize absolute cost values, since costs may vary significantly over time due to policy, regulation, and market conditions, it is still important to explore cost sensitivities, particularly for rSOC systems, which are currently at a relatively low technology readiness level (TRL). Unlike battery costs, which are comparatively mature and stable, rSOC costs are subject to substantial uncertainty. Therefore, we conducted a sensitivity analysis on rSOC CAPEX, varying from 10 k€/kW_FC_ to 1 k€/kW_FC_ to assess its impact on the overall LCOS across all considered countries (FC: fuel cell). The results are presented in Fig. 3e.

The analysis reveals that a reduction in rSOC CAPEX can significantly decrease LCOS. For example, LCOS ranges from 0.19 to 1.57 €/kWh under current rSOC costs but can be reduced to 0.16 to 0.59 €/kWh as rSOC costs decline. For certain countries, this corresponds to a maximum LCOS reduction of up to 80%. In addition, the sensitivity analysis highlights notable differences in LCOS stability across countries. Short-term storage-oriented countries, such as Estonia and Hungary, exhibit distinct behaviors. Estonia shows a pronounced LCOS sensitivity to rSOC CAPEX, whereas Hungary displays only minor variation. This difference arises because Estonia requires a shorter storage duration and hence a larger rSOC power capacity.

Interestingly, long-term storage-oriented countries, such as Switzerland, also show relatively limited LCOS sensitivity to rSOC CAPEX. This can be attributed to the hybrid storage configuration, in which LCOS consists of three main components: battery costs for short-term storage, rSOC costs for long-term storage, and storage tank costs. In Switzerland’s case, energy must be stored over extended periods, which can be ensured even with low rSOC installed capacities. There, the LCOS is mainly related to the storage tank costs.

Nevertheless, the sensitivity analysis clearly demonstrates that as rSOC technology matures from low to higher TRL, leading to reduced CAPEX, LCOS decreases across all countries considered to varying degrees. This highlights the strong potential of rSOC cost reductions to enhance the economic competitiveness of long-term energy storage systems in future decarbonized energy systems. For the detailed LCOS for each country, it can be seen in the Supplementary Table B9 in SI.

The effect of additional efficiency losses from the grid-side components and transformers is shown in Supplementary Fig. B3 in the SI, for two contrasting countries: Switzerland and Estonia. This analysis indicates that the impact of transformer efficiency (97.5% in either direction) is limited to approximately 3% on storage volume and LCOS across all countries. Countries relying on long-term storage, such as Switzerland, are more sensitive to round-trip efficiency, as storage constitutes a structural energy pathway with limited alternatives to avoid losses. In contrast, countries relying primarily on short-term storage (e.g., Estonia) require mainly operational flexibility, which can be more readily adjusted to compensate for  round-trip efficiency losses.

Pumped-hydro storage was also evaluated, though its applicability is limited to countries with suitable geographical conditions and a significant share of hydroelectricity in their energy mix^33,34^. Supplementary Table B10 in the SI summarizes the results for six countries where storage sizes are considerably larger due to the lower energy density of pumped-hydro storage compared to rSOC or batteries. For example, Switzerland, Spain, and Italy require storage sizes of 775, 331, and 197 m^3^/kW, respectively, significantly exceeding the sizes of other technologies. Despite this, in countries with geographic suitability and existing infrastructure, pumped-hydro storage remains a viable and effective option for long-term energy storage. However, due to its siting constraints, it cannot be deployed universally. In contrast, rSOC and batteries offer greater deployment flexiblslitty, particularly for decentralized or space-limited applications, making them complementary technologies within a diversified storage portfolio.

## Discussion

This study evaluates the technical viability and integration potential of an rSOC system equipped with a hybrid tank for fuel and CO_2_ storage at 75 bar. The proposed system achieves optimised efficiencies of 72.6% (SOFC) and 82.1% (SOEC) through advancements, such as ejectors, a hybrid storage tank, and Rankine cycle integration, resulting in a round-trip electrical efficiency of around 60% for rSOC operation at atmospheric pressure. These findings validate rSOC as a robust energy storage solution. Furthermore, the rSOC integration analysis highlights its adaptability in both current and future energy frameworks. In the current energy landscape, rSOC storage technology demonstrates environmental and economic benefits by responding to variations in electricity prices and electricity CO_2_ intensity. In the 2050 energy framework, rSOC is essential for managing renewable variability, offering advantages in solar-reliant regions, while batteries and hybrid strategies address short- and mixed-storage needs.

### Viability of rSOC technology in the current energy system

Overall, we have highlighted both the economic and environmental benefits of the integration of the rSOC system into the current energy mix while addressing key challenges. One assumption is the marketability of oxygen produced from electrolyzers, which has shown promise but requires further investigation into safe removal, storage, and transportaction^35,36^. Additionally, while the current energy mix scenario mainly focuses on operating costs, incorporating capital expenditure (CAPEX) is essential to assess the overall viability of the rSOC system within today’s energy landscape. Based on the 50/150 kW rSOC system and the storage requirements derived for the optimal trade-off case (Solution B), the CAPEX can be estimated using the cost parameters listed in Table 1 in the Methods section. The total investment is approximately 1.8 M€ for a 20-year system lifetime. When accounting for oxygen by-product revenues, the payback period is estimated at 4–5 years. Details on the economic assumptions can be found in Supplementary Methods S4 in SI. Moreover, the fixed rSOC system size of 50 kW SOFC/150 kW SOEC is small relative to national energy needs but can be scaled linearly. For instance, based on electricity import and export data from the Swiss Federal Office of Energy, Switzerland could reduce electricity imports significantly by utilizing storage technology to store all exported electricity. This strategy (i.e., rSOC systems-based storage) could achieve a maximum annual CO_2_ savings of 2 million tonnes, accounting for more than 4% of Switzerland’s total annual CO_2_ emissions.

Nevertheless, decentralized deployment of small rSOC plants at the district level is expected to fulfill localized energy demands effectively. Importantly, the potential impact of stack degradation and temperature variations, though not included here, deserves separate dynamic analyses. However, mode switching may reduce degradation rates, as supported by long-term rSOC demonstration studies^37,38^. Despite these limitations, the findings offer valuable insights and practical guidelines for integrating rSOC systems into the current energy framework. They emphasize the potential for decentralized applications, cost reductions with scaling, and future market opportunities, paving the way for rSOC technology to play a significant role in energy transitions.

### Viability of energy storage technology in the future energy system

As future energy systems prioritize reducing carbon footprints and minimizing investments, robust energy supply planning that integrates both long- and short-term storage solutions becomes crucial. Our analysis underscores the need for integrating both types of storage technologies to create a decarbonized energy framework.

rSOC systems are particularly advantageous for handling seasonal storage demands and can operate in electrolyzer mode for hydrogen or syngas production, thus supporting both energy and chemical industries. Their ability to co-generate heat at both industrial and residential temperature levels offers additional benefits. Conversely, batteries effectively manage daily fluctuations due to their rapid charge and discharge capabilities. Based on our results, most countries could benefit from adopting hybrid storage strategies, which combine the strengths of both technologies.

While this study considers cost analysis, it does not delve into extensive detail. Nevertheless, the results suggest that hybrid energy storage solutions can offer economic benefits across all evaluated countries, although the extent of cost reduction may vary across regions. These variations are closely linked to each country’s electricity mix. Additionally, the economic advantage of hybrid systems is expected to increase over time as technological maturity improves and investor confidence grows.

Future research should address transient behaviors, modular designs, and detailed cost analyses, as well as explore waste heat utilization for enhanced efficiency. While this study evaluates countries individually to reflect national-scale planning, future work could also consider cross-border energy exchange and regional interconnectivity as promising dimensions of long-term energy storage deployment. Finally, our results are derived for Europe, where harmonized, high-resolution datasets are available. The modeling framework is transferable, but extending it to other regions (e.g., the United States, Asia) can still be interesting to assess how the role of energy storage differs beyond Europe. Overall, this study presents energy storage technologies’ technical, environmental, and economic potentials in Europe, positioning them as pivotal for supporting renewable integration in the energy mix.

## Methods

### rSOC system modeling for energy conversion and storage

**Fig. 4:**
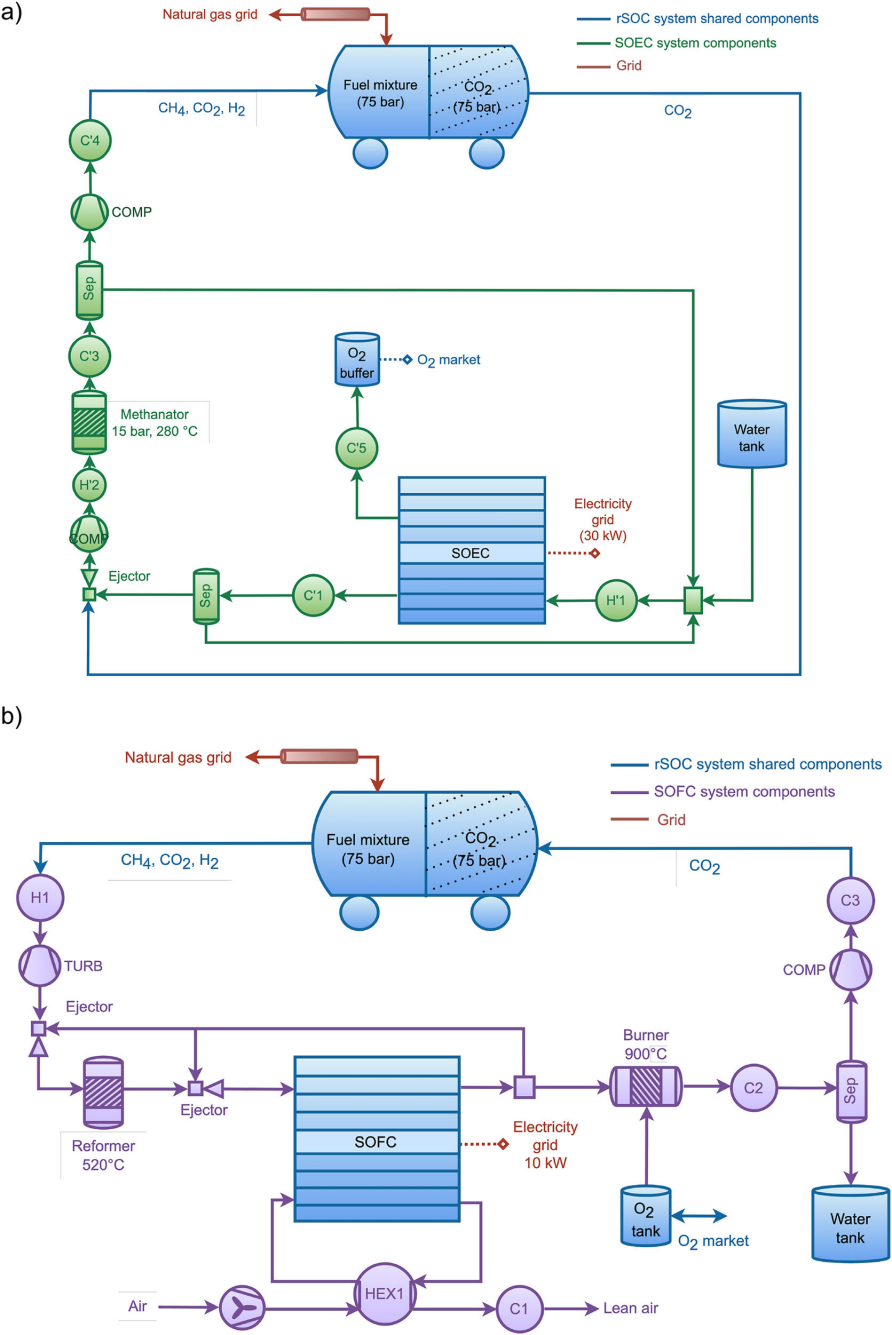
Integrated solid oxide system for methane production and power generation. **a** Integrated solid oxide electrolyzer-methanator-hybrid tank system for methane production and storage; **b** Integrated solid oxide fuel cell-hybrid tank system for electricity production. Note: solid oxide electrolyzer cell (SOEC), reversible solid oxide cell (rSOC), solid oxide fuel cell (SOFC), turbine (TURB), separator (SEP), and compressor (COMP).

Figure 4 presents an advanced rSOC-based energy conversion and storage system, highlighting shared and mode-specific components. Shared components, including the stack, hybrid storage tank, O_2_ buffer tank, and water tank, are marked in blue to facilitate seamless operation in both SOFC and SOEC modes. Distinct components of the SOFC system, such as the reformer, burner, and condenser, are highlighted in purple, while the methanator, essential for the SOEC system, is marked in green. The system is designed for flexible fuel management with direct connections to the natural gas grid, facilitating green methane synthesis and leveraging existing infrastructure.

The hybrid tank enables efficient storage of gaseous fuel and CO_2_. It has two compartments, one for gaseous fuel and another for CO_2_. The relative volumes of the compartments can be adjusted using a movable wall that can change its position according to the quantities of gaseous fuel and CO_2_. A piston-like mechanical device, a little greater fluid pressure entering the hybrid tank, or a combination of the two can be used to manage the wall movement. In the case of methane fuel, one mole of methane produces one mole of CO_2_, and vice versa. Preferably, similar pressure can be maintained on either side of the movable wall. The hybrid tank pressure can be chosen above the critical pressure of CO_2_ (73.77 bar). Although a higher pressure will result in a smaller hybrid tank, it will increase the capital and operating costs of fluid compression. This hybrid tank concept has been patented by our research group, and it is now in the process of being adopted by an industrial partner^39^. Overall, this design ensures the system’s immediate compatibility with current energy frameworks, paving the way for its integration into energy systems.

### Advanced SOEC system design and modeling

Figure 4a and Supplementary Method S1 in the SI illustrate the operation of the rSOC system in SOEC (30 kW) mode, coupled with a methanation unit and a hybrid storage tank. This study proposes an advanced design using an ejector that enables single-stage heating and expansion (75 bar to 15 bar), rather than the multi-stage processes typical of conventional systems (Supplementary Fig. A1 in SI), which often involve complex configurations with multiple components for pressure change, heat exchange, and interconnection. This simplification improves system efficiency and reduces infrastructure requirements. Although hydrogen is an attractive energy carrier due to its zero CO_2_ emissions during use, its large-scale deployment is constrained by the limited availability of dedicated transport infrastructure, such as pipelines. To address this challenge, a methanation unit is integrated with the SOEC, allowing hydrogen to be converted into methane. The produced methane can either be stored in a hybrid tank or injected directly into the existing natural gas grid, leveraging established infrastructure while reducing carbon emissions and improving commercial feasibility. This approach therefore provides a transitional pathway that utilizes current gas infrastructure while supporting the future development of hydrogen-based energy systems.

In conventional systems, methanation typically requires high-pressure hydrogen, which is usually achieved through multi-stage compression (as shown in Supplementary Fig. A1 in SI) or by operating the SOEC stack at elevated pressure. In contrast, the proposed ejector enables single-stage compression of atmospheric hydrogen by utilizing high-pressure CO₂ from the hybrid tank. This approach allows the SOEC stack to operate at atmospheric pressure, thereby avoiding the durability and stability challenges associated with high-pressure solid oxide cells.

As shown in Fig. 4a, water is heated to 750 °C through sequential heating, evaporation, and superheating (H'1) before entering the cathode side of the stack. The stack operates at atmospheric pressure in thermoneutral mode with an operating voltage of 1.32 V^40–42^, enhancing durability and commercial viability. Oxygen produced at the anode is cooled (C'5) for safe handling and stored in an O_2_ buffer tank connected to the oxygen market, whereas hydrogen and unconverted steam from the cathode are cooled (C'1) to condense water for recirculation. After electrolysis, methanation occurs using hydrogen produced in the stack and CO_2_ from the hybrid tank. To facilitate this process, an ejector is employed instead of a conventional turbine to reduce the CO_2_ pressure from the hybrid tank. The required heat for superheated steam production (H’1) is recovered from hot streams (e.g., C’1, C’3), and only a small additional heat input (via an electrical heater) is needed to reach the target temperature due to the minimum temperature difference constraint. In this configuration, high-pressure CO₂ serves as the motive fluid, and atmospheric-pressure hydrogen as the suction fluid. The compressed mixture is cooled (C'2) and sent to the methanator, where H_2_ and CO_2_ are converted to methane. The CH_4_-rich gas is further cooled (C'3) for water removal and compressed/cooled (C'4) to match hybrid tank conditions. The SOEC stack, methanator, and balance of plant (BoP) models have been validated with experimental data^43–45^. Key simulation parameters and process descriptions for the SOEC-methanation-hybrid tank system, modeled in Aspen Plus, are provided in Supplementary Table A1 of the SI.

Furthermore, to compare the proposed design with conventional benchmark systems, conversion efficiency is used as a key performance indicator. It is defined as the ratio of the chemical energy content of the produced methane to the total electrical power input to the system, and is calculated as follows: Eq. (1).

1 $${{{{\rm{\eta }}}}}_{{conversion}}=\frac{{{LHV}}_{{CH}4}\cdot \,{\dot{m}}_{{CH}4}}{{{P}_{{aux}}+P}_{{SOEC}}}$$

where ${\dot{m}}_{{CH}4}$ is the mass flow rate of methane produced (kg/s), ${{LHV}}_{{CH}4}$ is the lower heating value of methane (J/kg), ${P}_{{SOEC}}$ is the electrical power consumed by the solid oxide electrolyzer (W), and$\,{P}_{{aux}}\;{{\rm{represents}}}$ the total auxiliary power consumption (W), including components, such as compressors and pumps.

### Advanced SOFC system design and modeling

Figure 4b and Supplementary Method S2 in the SI show the rSOC system operation in SOFC (10 kW) mode by using the same stack, activated when electricity prices are high or renewable generation is insufficient. The stack operates at an average voltage of 0.772 V under nominal conditions^40–42^. The selection of a 10-kW stack was based on the market availability of single-stack rather than stack modules. However, the outcomes can readily be adapted to systems of larger sizes. The system utilizes stored high-pressure fuel, which is expanded to 15 bar and reformed adiabatically at 8.2 bar^46^ to produce syngas. The reformer achieves a low methane conversion rate, enabling up to 90% internal methane reforming within the stack. It balances exothermic reactions, reduces cooling needs, and lowers air blower power consumption. On the cathode side, air supplies oxygen for electrochemical reactions while maintaining maximum stack temperature at 800 °C with a 100 °C temperature gradient across the stack. Hot cathode (depleted) air exchanges heat with fresh air through HEX1 before cooling to atmospheric temperature, ensuring modular design and simplified construction. The AOG flow, containing unconverted fuel, is partially recirculated upstream using two ejectors to improve global fuel utilization and to avoid external steam production. The first ejector blends high-pressure fuel with AOG flow to precondition the reformer feed, optimizing temperature and steam-to-carbon (S/C) ratio. The second ejector mixes high-pressure syngas with AOG flow to precondition the stack feed, eliminating the need for additional heating and AOG re-pressurization. The remaining AOG undergoes oxy-combustion using oxygen from the SOEC-produced O_2_ buffer, ensuring complete fuel utilization. The resulting stream is cooled, and CO_2_ is separated and stored in the hybrid tank. The advanced dual-ejector setup improves thermal efficiency by removing two pre-heaters, simplifying system architecture.

To evaluate the advanced aspects of the proposed SOFC system design, a comparative simulation study was conducted against three reference configurations: two conventional industrial designs (No AOG and Warm AOG) and a less commonly applied design (Hot AOG recirculation). The No AOG configuration, which does not include AOG recirculation, relies on an external water supply and requires an additional heater to generate steam. This design is widely used in industrial SOFC systems due to its simplicity; however, it requires significant external water and leads to higher fuel losses to the burner. To mitigate these limitations, the Warm AOG design recirculates cooled AOG using a typical low-temperature compressor (250-300 °C) to overcome the pressure drops. This configuration eliminates the need for an external water supply but still requires heat exchange units and a compressor. The third configuration, Hot AOG recirculation, compresses the AOG flow directly without prior cooling, which reduces the need for an external water supply. However, this approach requires a compressor capable of operating at very high temperatures, limiting its commercial adoption.

For the proposed system and the three reference configurations, the SOFC stack, reformer, and BoP models have been experimentally validated^17,43–45^, with key parameters detailed in Supplementary Table A2 of the SI. Similarly, to compare the proposed design with conventional benchmark systems, the SOFC system’s electrical efficiency has been considered, which is defined as the ratio of the net electrical power output to the chemical energy input provided by the methane fuel (see Eq. 2).

2 $${{{{\rm{\eta }}}}}_{{electrical}}=\frac{{P}_{{SOFC}}-{P}_{{aux}}}{{{LHV}}_{{CH}4}\cdot \,{\dot{m}}_{{CH}4}}$$

where ${\dot{m}}_{{CH}4}$ is the mass flow rate of methane produced (kg/s), ${{LHV}}_{{CH}4}$ is the lower heating value of methane (J/kg), ${P}_{{SOFC}}$ is the electrical power produced by the solid oxide fuel cell (W), and *P*_aux_ represents the total auxiliary power consumption (W), including components, such as compressors and pumps.

### rSOC system heat cascade analysis

For both SOFC and SOEC modes, heat integration is considered by conducting a heat cascade analysis. It is important to clarify that SOEC stacks typically operate near the thermoneutral potential and therefore do not generate heat within the stack during electrolysis. Nevertheless, at the system level, there is still net waste heat available in the medium- and low-temperature range due to the presence of the exothermic methanation reaction and cooling of outlet streams from the stack. However, the high-temperature heat source is still required to superheat the steam to the necessary SOEC stack inlet temperature. rSOC technology uniquely enables continuous heat co-generation in both SOEC and SOFC modes, offering steam production opportunities for industrial processes and district heating, or electricity generation through a RC. This study explores the integration of an RC to improve overall system efficiency and compares performance across configurations. While the modeled system is small and assumes the use of a microturbine, extensive feasibility studies support its application^47,48^. Specific modeling details of the RC, including design considerations, are outlined in existing research^49,50^_._

### Energy system integration

Integrating the rSOC technology within energy systems alongside other technologies necessitates detailed modeling of each component and the overall system. This study explores the implementation of rSOC within two distinct electricity mixes: the present, characterized by the price and the carbon intensity, and the future one, entirely based on renewable energy sources. The future electricity generation and consumption profiles are based on the previous studies^32,51^, while current profiles have been obtained from our industrial contacts, and spot market prices were used for the year 2019. The generic methodology for categorizing yearly data into typical days can be found in Supplementary Method S5, Supplementary Figs. A4 and A5 of the SI, demonstrating its applicability across the various profiles.

**Fig. 5:**
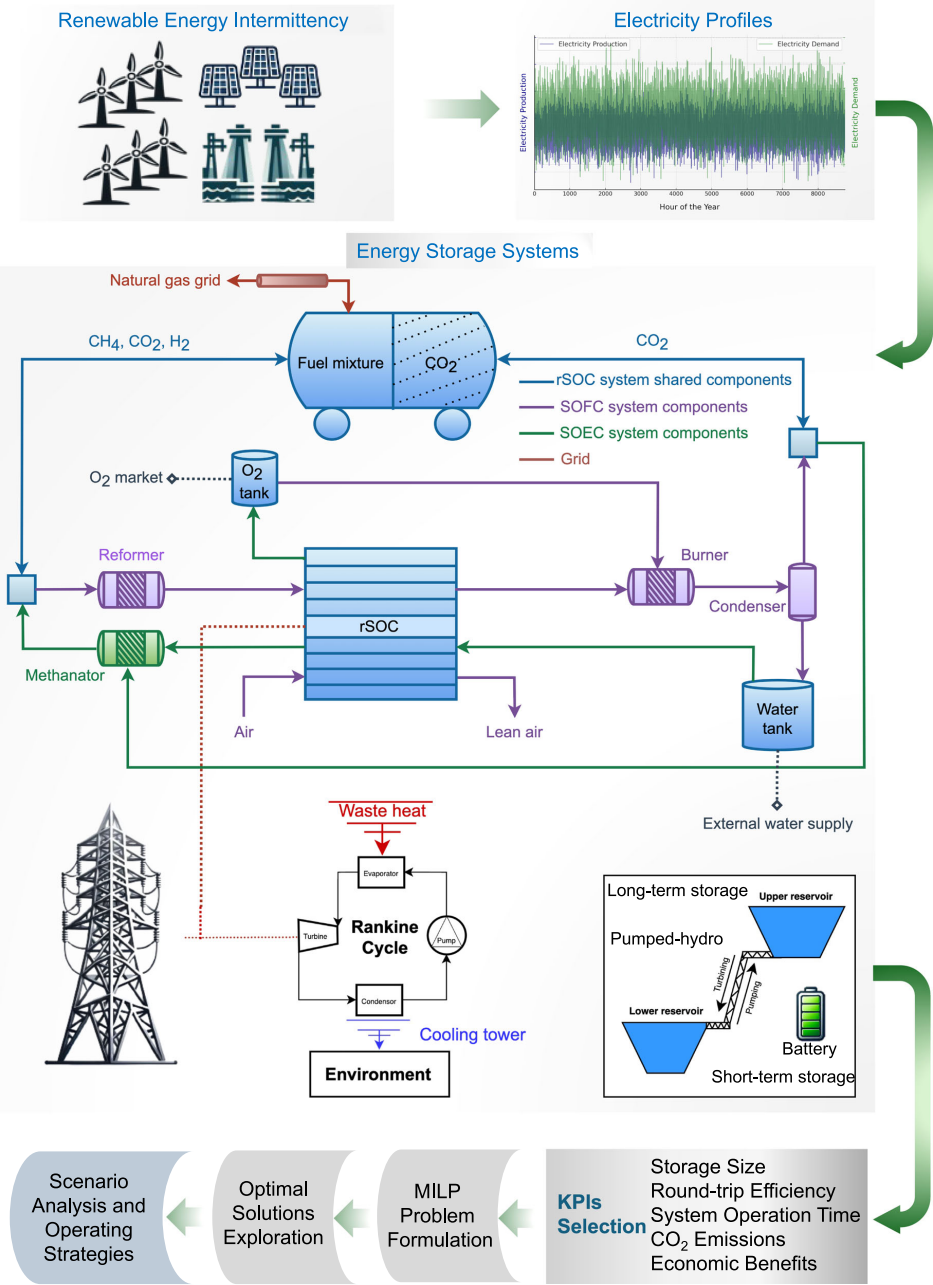
Process integration and optimization framework. Optimization framework using OSMOSE - A tool for process integration and optimization. Note: reversible solid oxide cell (rSOC), solid oxide electrolzer cell (SOEC), solid oxide fuel cell (SOFC) turbine (TURB), separator (SEP), and compressor (COMP).

These electricity profiles serve as input to the optimization problem that is solved using OSMOSE, an in-house decision-making tool that can perform heat/resource integration and multi-period/multi-objective optimization. It uses process models, utility models, cost models, and environmental impact models to compute the relevant objective functions. Figure 5 provides an overview of the optimization framework, which incorporates several main components. The first component involves the K-Medoids clustering of hourly electric grid profiles for current and future energy systems into typical days. Due to the high variability of the profiles and the smoothing nature of the typical days clustering approach, a security factor of 10% was added for the minimum power requirements. The second component encompasses three energy storage systems (energy technologies), including the newly proposed rSOC system with the hybrid storage tank. The rSOC system is designed to optimize energy storage capabilities and waste heat valorization through an RC. The third component outlines the optimization framework, starting with the characterization of energy technologies based on their inputs, outputs, and modeling variables. The optimization aims to determine the optimal interaction between various technologies and the electricity mix, comparing the performance of the rSOC system against established energy storage solutions, such as batteries and pumped-hydro. To perform the process and heat integration, a mixed integer linear programming (MILP) problem has been formulated in A Mathematical Programming Language (AMPL) and solved using the CPLEX solver, minimizing an objective function, such as OPEX or environmental impact, while adhering to predefined constraints. Several key performance indicators, such as storage capacity, operating time, and round-trip efficiency, are retrieved and visualized. Detailed explanations of KPI selection can be found in Supplementary Method S0 of the SI.

The modeling of storage facilities requires careful consideration to the time sequence. The stored quantity (molecules, water or electricity) must be transferred to the next hour, where a portion of it becomes available for interaction with the system, either in charge or discharge mode. This modeling requires three distinct units: the main storage, the charging, and the discharging units. The main storage level at time t $\left({{Level}}_{{main}}\left[t\right]\right)$ is defined in Equation (3), with relevant parameters compiled in Table 1. This equation refers to the mass or energy conservation of a stored quantity of the previous time step $\left({{Level}}_{{main}}\left[t\right]-1\right)$), where the incoming (${\dot{F}}_{{in}}$) and outgoing (${\dot{F}}_{{out}}$) flows are added and subtracted, respectively. The round-trip efficiency is calculated as the product of the charging and discharging efficiencies. As expected, the battery surpasses the other two storage technologies in this aspect. The parameter σ is derived from the self-discharge rate (SDR) as the efficiency to effectively transfer the stored energy quantity to the next hour. For this study, an SDR value of 0 is assumed for both rSOC and pumped-hydro storage, while a value of 3.6% per month is selected for the battery, representing an average value from various sources for relatively large batteries^52,53^. Pumped-hydro storage shows good round-trip efficiency for long-term storage, but it requires large storage volume due to low value of potential energy (referred as volumetric energy density in Table 1).

3 $${{Level}}_{{main}}\left[t\right]=\sigma \cdot {{Level}}_{{main}}\left[t-1\right]+{\eta }_{{sto},\,{charg}}\cdot {\dot{F}}_{{in}}\left[t\right]-{\eta }_{{sto},\,{discharg}}\cdot {\dot{F}}_{{out}}$$

**Table 1:** Storage and cost^17^ parameters for rSOC^1^, pumped-hydro and battery^65–67^

	Battery	Pumped-hydro	rSOC system
Round-trip efficiency (${{{{\rm{h}}}}}_{{RT}},\,\%$)	89	81	60 (this study)
Self-discharge rate (SDR, %/month)	3.6	0	0
Sigma (σ, %/h)	99.995	1	1
Volumetric energy density (${E}_{{dens},{vol}}$, kWh/m^3^)	450	1.4 ^a^	760 ^b^
Investment cost (CAPEX)	0.1 k€/kWh	-	10 k€/kW + 5 k€/m^3^
Estimated lifetime (future), years	15	-	10 (rSOC), 15 (tank)

^a^Potential energy for 1 m^3^ of water with assumed 500 m height for a dam. ^b^Volumetric energy density of stored CH_4_ at 75 bar (more details can be found in Supplementary Fig. A3 of the SI).

### Energy system model assumptions

To improve the reliability and relevance of the rSOC system, several assumptions were made that consider industrial constraints. The SOFC system is designed to meet its S/C ratio above 1.5 through the water contained in the AOG flow, avoiding the use of external water sources. This adds another constraint on the AOG recirculation ratio. A high AOG recirculation ratio reduces the consumption of fresh fuel, thereby increasing the overall fuel utilization. Further, to prevent the degradation of the stack, the AOG flow should have at least 10% unconverted fuel by considering stack manufacture specifications^2,17,21,44^.

Another key assumption is related to the SOEC system. Air is traditionally used as a sweep fluid to expel the oxygen to mitigate safety risks, and pure oxygen removal does not markedly affect system performance or lifetime. Further, collecting and transporting oxygen can also complicate its practical use. Despite these challenges, oxygen has substantial demand in industries, such as healthcare, metallurgy, chemical processing, and oxy-combustion. Selling high-purity oxygen could significantly improve the economic feasibility of rSOC systems^54,55^. Further, utilizing this by-product promotes environmental sustainability by optimizing material use and reducing waste. Ongoing advancements in safety protocols and technologies are likely to further enable the efficient removal of oxygen, potentially eliminating the need for additional sweep gases where a separation step is needed to recover O_2_^56,57^. Thus, in this study, for operating cost (OPEX) as the objective function, two scenarios are considered: excluding revenue from selling O_2_ and including revenue from selling O_2_. Furthermore, two operational strategies are explored: flexible rSOC operation, where the system autonomously decides when to activate the system, and fixed rSOC operation, which maintains continuous system operation throughout the year. Finally, oxygen is priced conservatively at 4 €/kg^58^, reflecting its market demand.

This study incorporates several other crucial assumptions. The simulation model uses no costs related to water usage or purification, thanks to the water recirculation feature of the rSOC system. The conversion of high-quality waste heat into electricity is accounted for in the system’s overall electrical efficiency, and potential revenue from low-quality heat is not included in the analysis.

When considering the integration of battery storage alongside the proposed rSOC system into the future energy system to determine the optimal strategy for energy storage, several constraints related to battery usage are included, addressing the risks associated with high state of charge (SoC)^59^. Prolonged high SoC levels have been linked to reversible and irreversible damage to batteries and accelerated calendar ageing^60^. Consequently, the model stipulates that batteries should not exceed 90% SoC for more than 48 consecutive hours and should not maintain more than 60% SoC for longer than one month. These precautions are designed to keep the long-term SoC within a safer range of 30–60% of maximum capacity^61^.

### rSOC critical phases - mode switching

Critical phases in rSOC systems generally include SOFC, SOEC, and hot-standby modes, as well as startup and shutdown. It is important to note that startup and shutdown durations for rSOC can vary between 15 and 24 h. Reliable data on these phases is scarce, making model validation challenging. Moreover, startup procedures can differ based on customer requirements and operational contexts, introducing further uncertainty with limited guidance on outcomes. This can potentially obscure the core focus of this study. Therefore, for clarity and to maintain the focus on the most impactful aspects of rSOC operation, this study assumes continuous operation of the rSOC system, excluding the startup and shutdown phases, which require more precise thermal management strategies^62,63^.

Furthermore, this study does not include potential degradation (temperature swing impacts) of the stack. Research indicates that mode switching does not significantly affect stack degradation^11,37^, and may even result in reduced degradation rates. Temperature fluctuations are more closely associated with stack conditions and BoP process control. A comprehensive analysis of these concepts is beyond the scope of this study. However, the model incorporates a reaction time between mode switches, which is critical and can significantly influence the results. During this period, ${\tau }_{{crit}},\,$ the rSOC system is unavailable for 15 minutes^38,64^. This inclusion accurately reflects the operational dynamics of the rSOC system, enhancing the realism of the model.

4 $${\varepsilon }_{{rel},\,{OPEX}}=	{N}_{{switch}}*{\tau }_{{crit}}*{P}_{{SOFC}}*\left({C}_{{OPEX},\,{elec},\,{sell}}-{C}_{{OPEX},\,{elec},\,{buy}}\right) \\ 	+\left(\frac{{N}_{{switch}}}{2}\right)*{\tau }_{{crit}}*\dot{{m}_{O2}}*{\rho }_{O2}$$

5 $${\varepsilon }_{{{rel},{co}}_{2}}={N}_{{switch}}*{\tau }_{{crit}}*{P}_{{SOFC}}*\left({E}_{{CO}2,{elec},\,{sell}}-\,{E}_{{CO}2,{elec},\,{buy}}\right)$$

In this analysis, the availability of the rSOC system is not considered directly in the optimization step; rather, it is calculated a posteriori on the optimized results. To define an upper limit on the effect of mode switching, the same critical times are considered for all mode switches. Eqs. (4) and (5) provide a conservative estimate of the penalties on the objective functions (${\varepsilon }_{{rel},}$). In these equations, ${N}_{{switch}}$ the number of mode switches, ${P}_{{SOFC}}$ is the maximum power output, $\left({C}_{{OPEX},{elec},{sell}}-\,{C}_{{OPEX},{elec},{buy}}\right)$ is the average difference in OPEX and $\left({E}_{{CO}2,{elec},{sell}}-\,{E}_{{CO}2,{elec},{buy}}\right)$ is the average difference in CO_2_ emissions. The second term of Eq. (2) accounts for O_2_ revenue losses, assuming that only half of the critical period affects the SOEC mode or O_2_ production. ${m}_{O2}$ is the nominal mass flow rate of O_2_, and ${\rho }_{O2}\,$ is its market price.

### Key performance indicators and cost metrics calculations

Key performance indicators (KPIs) are used to evaluate and compare the optimization results. In this study, both environmental and economic metrics are used and described below. While the environmental impact is relatively straightforward, expressed in t CO_2_-eq/year, the financial aspect needs clarification on the assumptions and other calculations. The economic performance of each storage technology is evaluated using the LCOS, calculated from the optimized storage capacity and annual energy throughput, together with technology-specific investment costs and lifetimes provided in Table 1. CAPEX include the conversion system and, for the rSOC technology, the associated storage tank. Investments are annualized, and fixed annual maintenance costs equal to 2% of initial CAPEX. For the hybrid storage strategy, the battery is sized to cover short-term power demand, assuming a storage duration of 10 h at maximum power, while the rSOC system is sized to the minimum rated power required to balance long-term energy flows, providing an upper-bound estimate of the hybrid LCOS. A detailed description of the CAPEX formulation, annualization, replacement costs, and LCOS calculation is provided in Supplementary Method S4 of the SI.

## Supplementary information

Supplementary information

Transparent Peer Review file

## Source data

Source data

## Data Availability

The paper and its Supplementary Information (SI) contain process explanation, results, figures and tables that display all the data and information that was used, created and obtained for this study. Moreover, source data are provided with this paper. The datasets generated during and/or analyzed during the current study are available from the corresponding author on request. Source data are provided with this paper.
