# Supplementary material for: Optimizing reversible solid oxide cell technology for long-term energy storage in Europe’s decarbonized frameworks
Source: Nat Commun. 2026 Jun 25;17:8071. doi: 10.1038/s41467-026-74229-z (PMC13454186; doi:10.1038/s41467-026-74229-z)
Supplement: Supplementary file 1 [file 41467_2026_74229_MOESM1_ESM.pdf]

Supplementary Information (SI)

Optimizing Reversible Solid Oxide Cell Technology for Long-Term Energy Storage in Europe's Decarbonized Frameworks

Xinyi Wei[1,2,*,#], Arthur Waeber[1,#], Shivom Sharma[1], Jan Van herle[2,*], Francois Maréchal[1,*]

[1]Industrial Process and Energy Systems Engineering-École Polytechnique Fédérale de Lausanne (EPFL), Sion, CH-1950, Valais, Switzerland

[2]Group of Energy Materials-École Polytechnique Fédérale de Lausanne (EPFL), Sion, CH-1950, Valais, Switzerland

*Corresponding author E-mail: xinyiwei.epfl@gmail.com; jan.vanherle@epfl.ch; francois.marechal@epfl.ch

[#] These authors contributed equally.

Supplementary Note A: Supplementary Methodology

Supplementary Method S0: Supportive Literature and Key Aspects

The state-of-the-art has already been presented in the Introduction section of our paper. For interested readers, this section in the SI presents some additional studies on rSOC system. Subsequently, this section covers performance criteria and several key aspects of our study.

Despite being in the early stages of commercialization, rSOC technology has demonstrated substantial potential for managing renewable energy sources, particularly in energy systems with high renewable share. For rSOC, high efficiency, fuel flexibility and heat production underscore their attractiveness for future energy system management. The ability of technology to switch between modes, namely electrolyzers and fuel cells, supports energy systems with a high renewable share, making it a promising solution for clean and sustainable energy systems[1]. Mogensen et al.[2] also emphasize rSOC's potential in the transportation sector and grid connections, particularly for seasonal storage, thus positioning it as a critical component of future energy infrastructures.

Giulio et al.[3] describe a grid-connected rSOC system within a power-to-X plant that primarily generates hydrogen during periods of low electricity prices. This hydrogen is then used to produce electricity during peak demand, achieving an annual round-trip efficiency between 55 and 70%. By utilizing wind power, the system also significantly reduces CO_2 emissions compared to traditional energy systems. Similarly, Wu et al.[4] highlight the capabilities of an experimental rSOC system that uses CO_2 for long-term energy storage, achieving round-trip efficiency of about 86% under high-pressure operations. This study underscores the robustness of the system in both power-to-gas and gas-to-power conversions.

Further exploring the economic aspects, Evan et al.[5] assess the techno-economic viability of rSOC systems, computing round-trip efficiencies of 53-54% and competitive costs relative to contemporary battery technology. They advocate for a variable volume floating piston tank to increase energy density. Jensen et al.[6] extend this discussion by combining rSOC with underground storage of CO_2 and CH_4, achieving an efficiency of over 70% and presenting a cost-effective alternative to conventional pumped-hydro storage.

Moreover, Wendel et al.[7] and Butera et al.[8] contribute to optimizing rSOC operation. Wendel et al. establish that operating at an average current density of 0.695 A/cm^2 leads to promising energy storage performance with optimal efficiency observed above 70% fuel utilization. Butera et al. explore storing electricity as synthetic natural gas using pressurized rSOC, attaining efficiency up to 79% under high-pressure operating conditions and noting that waste heat recovery can further enhance the performance of the system by an additional 5-10%. Lastly, Zhang et al.[9] analyze the economic and environmental advantages of integrating hydrogen into natural gas pipelines via rSOC technology, demonstrating significant potential benefits for energy systems and pipeline stability.

In this study, we considered various criteria to evaluate the performance of rSOC system. The optimization of individual systems remains underrepresented in the existing literature. Initially, individual system efficiency has been computed, which is crucial for optimal process design. Some studies focus on high-pressure rSOC operations, which offer potential efficiency gains, but commercialization remains a significant challenge. Even at the laboratory scale, high-

pressure rSOC systems are underexplored due to technical and economic hurdles. Until high-pressure operation is feasible at a large scale, atmospheric pressure rSOC systems continue to play a critical role in technology commercialization. It is still necessary to prioritize developing strategies that improve system efficiency under atmospheric conditions while exploring innovative solutions to accelerate the adoption of high-pressure rSOC in commercial settings.

Round-trip efficiency is another important criterion for evaluating rSOC technology, as it directly influences its competitiveness compared to other energy storage technologies. Waste heat availability is one of the notable advantages of the rSOC system, as high-temperature operation enables the simultaneous generation of both electricity (or fuel) and high-quality heat. This co-generation capability enhances overall system efficiency by allowing the recovery and use of waste heat, a factor that other storage technologies often lack. Further exploration of its round-trip efficiency and waste heat management strategies is essential for demonstrating its superiority over competing storage technologies in real-world applications.

The type of e-fuel and storage size are critical for evaluating rSOC systems, as they serve two primary purposes. First, they standardize energy storage measurements, offering guidance to industrial professionals to estimate the required space for storing the electricity as fuel. Second, by considering the physical footprint and storage efficiency, these factors enable accurate comparisons with other energy storage technologies, such as batteries and pumped hydro. The type of e-fuel influences storage conditions due to varying densities and energy contents, while storage size determines the scalability and applicability of the technology. For example, hydrogen storage typically requires larger volumes compared to other gases due to its lower volumetric energy density. This understanding clarifies the spatial and technical requirements for energy storage and allows stakeholders to make informed choices in planning and deploying storage solutions. Therefore, a thorough analysis of the type of e-fuel and storage size is essential for fair comparisons with other technologies, considering energy density and spatial dimensions in practical applications.

Integrating rSOC with renewable energy sources is important for shaping the future energy landscape. rSOC is envisioned primarily for long-term renewable energy storage applications in the future, but it can still be utilized in the current energy market. The system is able to store electricity during periods of low cost and/or low carbon intensity, then produce the electricity during periods when electricity prices and carbon emissions are higher. Additionally, it co-produces O_2, which is a potentially marketable by-product. The immediate economic benefits are currently limited due to the low cost of fossil-fuel-generated electricity. However, looking ahead to the year 2050, when the goal is net-zero emissions, the availability of renewable electricity will increase in various countries. Therefore, integrating long-term energy storage into futuristic energy systems becomes indispensable. rSOC can manage variability in renewable electricity production by storing excess electricity and generating the electricity when needed. The rSOC integration into the energy system not only aids decarbonization efforts but also enhances energy security and grid stability.

It is notable that only a few studies have covered all the aforementioned criteria necessary for understanding the potential application of rSOC in energy systems. This study incorporates key aspects, as follows:

- Operating an rSOC system at high pressure can enhance round-trip efficiency by minimizing the pressure difference needed for fuel storage, which typically requires compressors and turbines. In literature, the round-trip efficiency computation is not

always realistic, due to the challenges in deploying commercially viable high-pressure stacks. This study optimizes the use of storage pressure by operating the stack at atmospheric pressure, simplifying the system while addressing the practical challenges and market conditions that impact the feasibility of high-pressure operations.

- A simple and compact design is essential when integrating rSOC into the energy system. In the case of SOFC mode, this study focuses on an efficient system design that converts heat into chemical energy, improves fuel utilization, minimizes the number and total area of heat exchangers, and reduces the size of the water purification unit.
- Instead of storing methane and CO_2 separately, this study uses a novel hybrid storage tank design that simultaneously stores both gases, a concept patented by our research group. The objective is to identify optimal ratios for both fuel and CO_2. Given the current state of CO_2 grid development, only natural gas and electricity grids are considered.
- Although integrating rSOC into the present energy system might not offer significant economic benefits, assessing the environmental impact and verifying economic assumptions is crucial. The study explores the implementation of rSOC technology in the current situation, having a small share of renewable energy, to determine the potential benefits.
- The market potential of rSOC primarily lies in future renewable energy storage. This study uses renewable electricity generation profiles across Europe for the year 2050 to develop guidelines for rSOC applicability in each country. If deemed suitable, the study provides recommendations on the optimal storage sizes.
- This study has also integrated batteries and pumped-hydro storage, applying them to the year 2050 renewable electricity profiles across various countries. This analysis aims to identify the benefits of hybrid energy storage technologies and provide guidelines for potential market opportunities.

Supplementary Method S1: SOEC system design

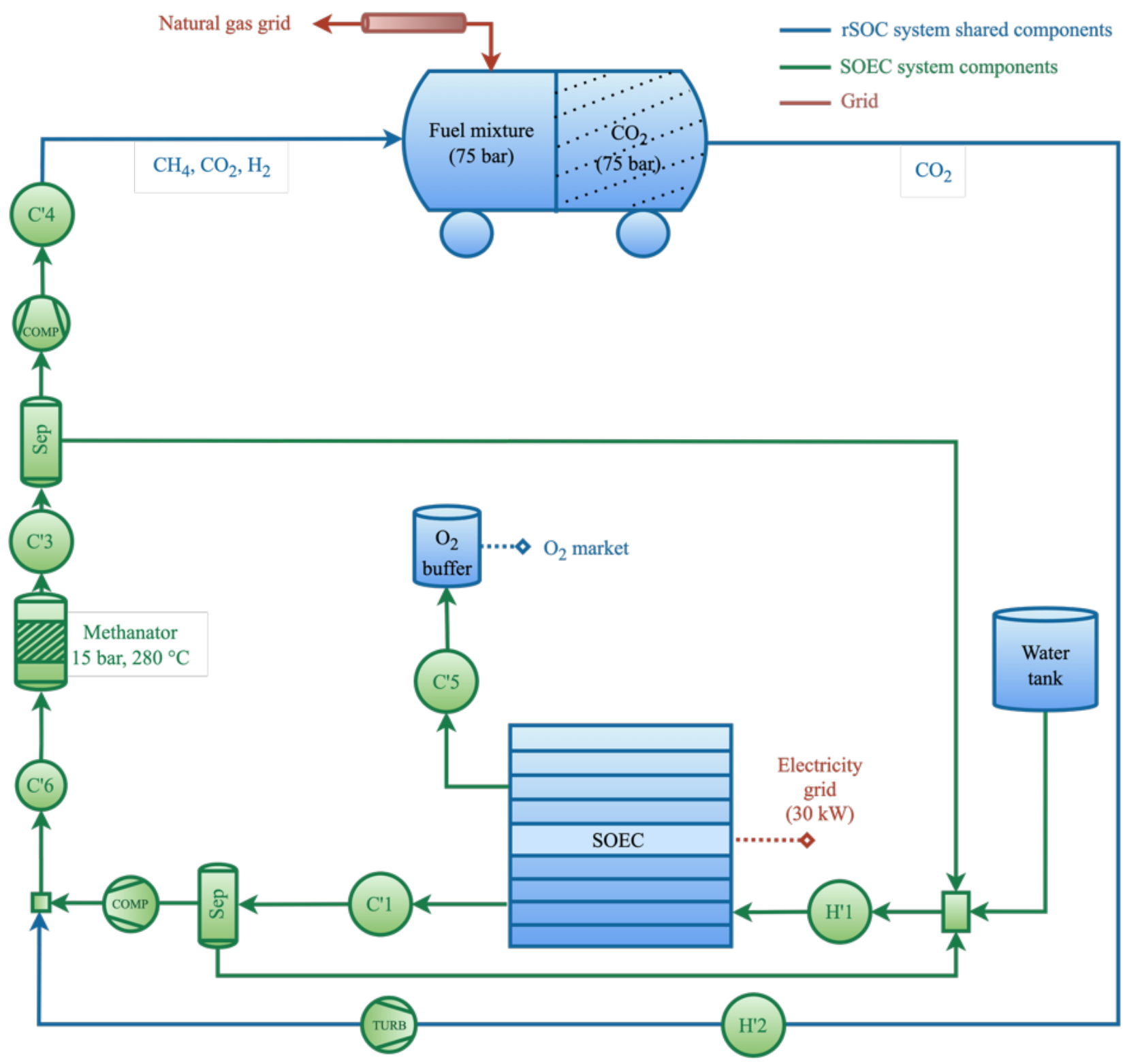

Supplementary Figure A1: Traditional SOE-Methanator Hybrid Tank System. Solid oxide electrolyzer-methanator-hybrid tank system for methane production and storage – traditional design

Supplementary Table A1: Key parameters used in the modelling of SOEC-methanator-hybrid tank system

Parameter	Value
Stack power capacity, kW	30
Stack operating voltage (nominal condition), V	1.32
Stack current density, A/m^2	4000
SOEC operating temperature, °C	750
SOEC fuel conversion rate	0.9
Electrical heater efficiency	0.95
Methanator operating temperature, °C	280
Methanator operating pressure, bar	15
Compression or expansion ratio	< 5
Mechanical efficiency of compressor	0.85
Isentropic efficiency of compressor	0.8
Mechanical efficiency of turbine	0.85
Isentropic efficiency of turbine	0.85
Hybrid tank pressure, bar	75

Supplementary Method S2: SOFC system design

Supplementary Table A2: Key parameters used in the modelling of SOFC systems

Parameter	Value
Hybrid tank pressure, bar	75
Reformer operating pressure, bar	8.2
Reformer inlet temperature, °C	603
Reformer outlet temperature, °C	520
Methane conversion rate in reformer	0.1
Internal reforming ratio in stack	0.9
Steam to carbon ratio before the stack	> 1.5
Stack capacity, kW	10
Stack pressure drop, mbar	< 100
Stack operating voltage (nominal condition), V	0.772
Stack area specific resistance (nominal condition), mΩ·cm2	350-420
Stack inlet temperature, °C	720
Stack outlet temperature, °C	800
Unconverted fuel mole fraction	> 0.1
Burner operation temperature, °C	900
Warm AOG blower operating temperature, °C	120
Warm AOG blower mechanical efficiency	0.85
Warm AOG blower isentropic efficiency	0.8
Warm AOG blower pressure increased, bar	0.25
Hot AOG blower operating temperature, °C	800
Hot AOG blower mechanical efficiency	0.65
Hot AOG blower isentropic efficiency	0.6
Hot AOG blower pressure increased, bar	0.25
Compressor mechanical efficiency	0.85
Compressor isentropic efficiency	0.8
Turbine mechanical efficiency	0.85
Turbine isentropic efficiency	0.85

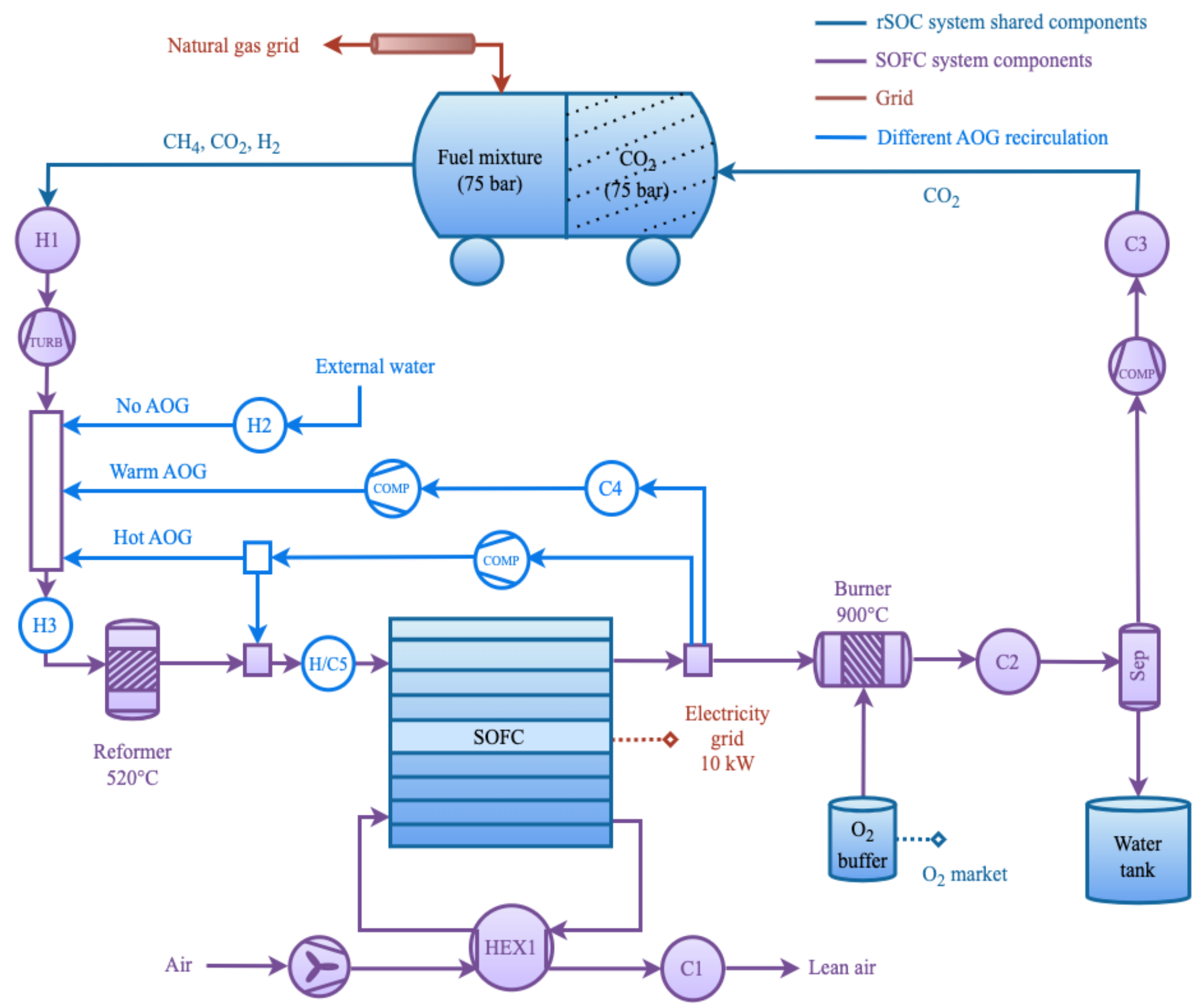

Supplementary Figure A2: Traditional SOFC-Hybrid Tank System. SOFC systems using external water and compressors for AOG recirculation – traditional designs

Supplementary Method S3: Hybrid storage tank volume

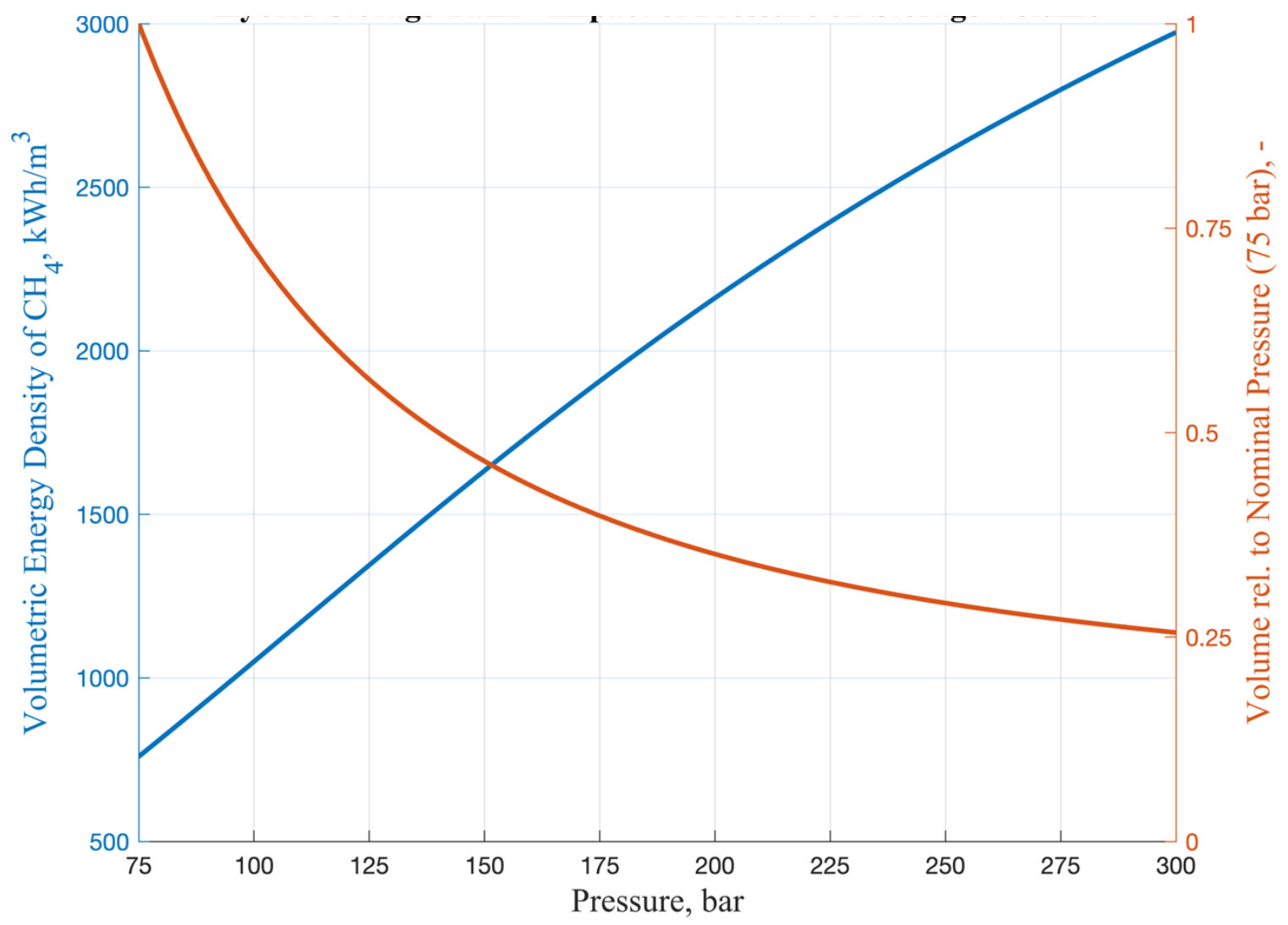

Supplementary Figure A3: Fuel Energy Density and Relative Tank Volume vs Pressure. Volumetric energy density of CH_4 at 25°C as a function of pressure, and relative volume of the hybrid storage tank (i.e., volume at a given pressure/volume at 75 bar)

Supplementary Method S4: Economic analysis

Based on the storage size (S) and the annual energy throughput (E) obtained from the optimization results, along with the unit investment cost (C_{inv}) and the component lifetime (n) from Table 1 in Methods, we can calculate the total investment cost (CAPEX), the annualized investment cost ($\text{CAPEX}_{\text{ann}}$), and the Levelized Cost of Storage (LCOS) for each storage technology. Note that for the rSOC storage strategy, both CAEPX of the rSOC system and the storage tanks simply summed up.

The project lifetime (n_{project}) is 20 years, and the interest rate is $i = 3\%$. Here, S_u represents the sizing of a storage component in [kW] or [kWh], $C_{\text{inv},u}$ is the corresponding normalized investment cost in [€/kW] or [€/kWh], $f_{\text{rep, u}}$ is the fraction of a full unit required to complete replacements over the project lifetime, and $t_{\text{rep, u}}$ is the time at which each replacement occurs [years], hence the lifetime (n) of the unit (u).

$$CAPEX_u\,[€] = S_u \cdot C_{inv,u} + \frac{f_{rep,u} \cdot S_u \cdot C_{inv,u}}{(1+i)^{t_{rep,u}}},\; f_{rep,u} = \frac{(n_{project} - n_u)}{n_u} \quad [S4.1]$$

for u in {Battery, rSOC, storage tank}

$$CAPEX_{ann,u}\,[€/yr] = CAPEX_u\,[€] \cdot \frac{i \cdot (1+i)^{n_{project}}}{(1+i)^{n_{project}} - 1}\,[1/yr] \quad [S4.2]|$$

$$C_{maint,u}\,[€/yr] = 2\,[\%] \cdot CAPEX_u \quad [S4.3]$$

$$\text{LCOS}\,[€/kWh] = (CAPEX_{ann,u}\,[€/yr] + C_{maint,u}\,[€/yr])\,/\,E\,[kWh/yr] \quad [S4.4]$$

For hybrid storage strategy, an estimation based on the optimized results can be computed using a storage duration of 10 hours at maximum power (P_{max}) for the battery and the minimum rated power ($P_{\text{rated, min}}$) for the rSOC in Table B9. This estimation assumes that the power requirements are fulfilled by the battery (short-term) system and that the energy balance is fulfilled by the rSOC (long-term) storage system, resulting in an upper bound of the levelized cost of storage for hybrid strategy.

$$CAPEX_{hybird}\,[€] = CAPEX_{Batt} + CAPEX_{rSOC} + CAPEX_{tank} \quad [S4.5]$$

Using Equation [S4.1] with $S_{Batt} = P_{max}$ [kW] · 10 [h], $S_{rSOC} = P_{\text{rated, min}}$ [kW] and $S_{tank} = S_{\text{tank, min}}$ [kWh]

Finally, using Equation [S4.2] for annualizing, and [S4.3] for computing the maintenance costs:

$$\text{LCOS}_{\text{Hybrid}}\,[€/kWh] = (CAPEX_{ann,\ hybrid}\,[€/yr] + C_{maint,\ hybrid}\,[€/yr])\,/\,E_{rSOC}\,[kWh/yr]$$
[S4.6]

Supplementary Method S5: Typical days clustering

It is essential to accurately characterize the yearly electricity profiles that accurately represent the trends while maintaining computational efficiency. This study explores the implementation of rSOC within two distinct electricity mixes: the present, which mainly contains fossil carbon, and the future, which will have a big share of renewable energy. The future electricity generation and consumption profiles are based on previous studies, while current profiles have been obtained from our industrial contacts. The electricity profiles under study are distinguished by varying characteristics. In the current energy mix, the price and CO_2 intensity profiles are crucial for the optimal integration of rSOC technology, focusing on the operating cost and environmental emissions as the key performance indicators (KPIs). Conversely, the future energy mix focuses on storage solutions that align with intermittent renewable energy production and fluctuating demand, thus prioritizing energy generation and consumption data for analysis.

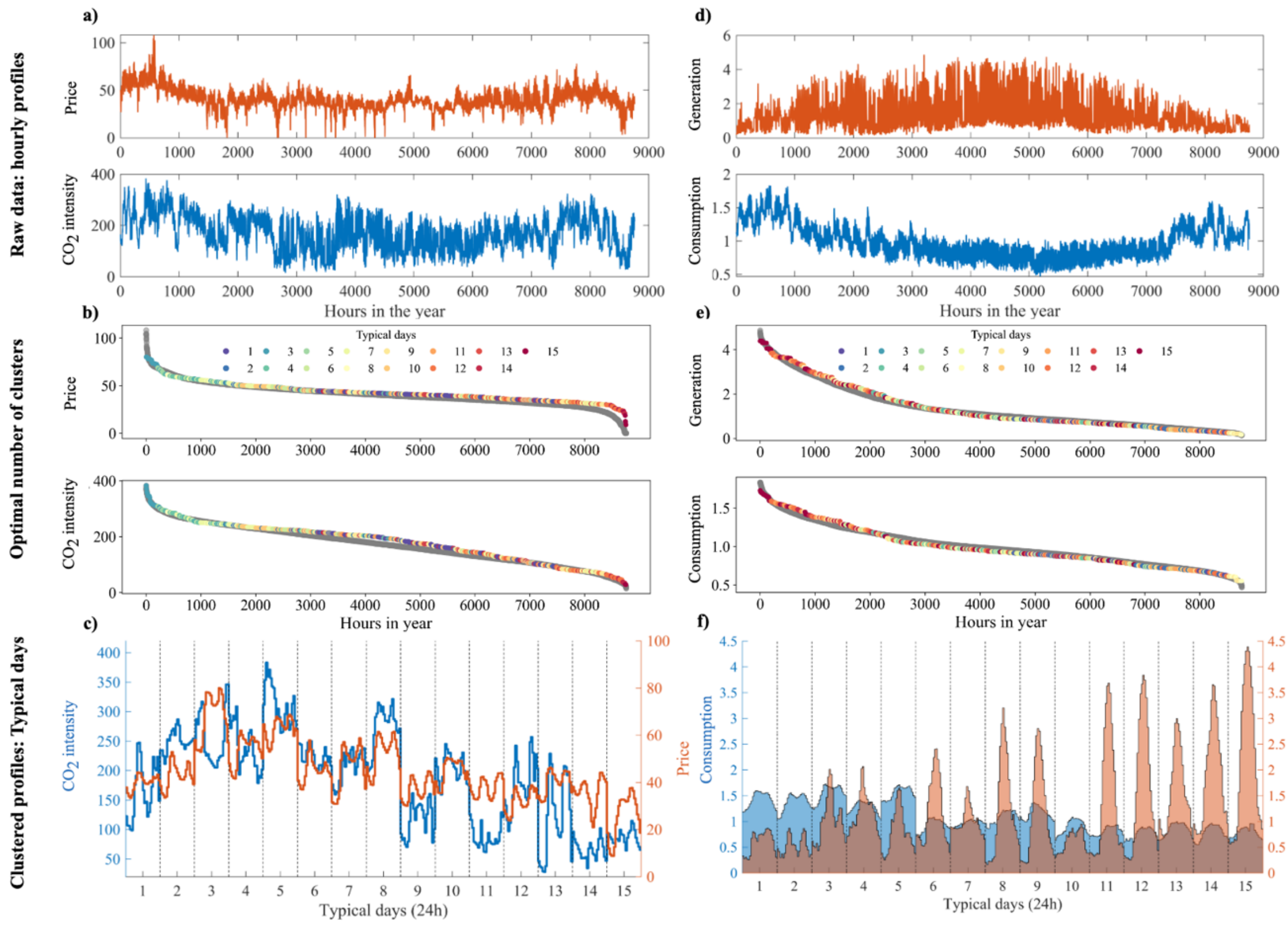

Supplementary Figure A4: Typical Day Clustering Methodology for Swiss Electricity Profiles. Generic methodology for categorizing yearly data into typical days for both current and future electricity profiles for Switzerland. Original profiles with hourly resolution for (a) current and (d) future. Performance of clustering for (b) current and (e) future profiles. Obtained typical days profiles for (c) current and (f) future.

Supplementary Figure A4 illustrates the clustering procedure for Switzerland's energy profiles for the current year and the year 2050. Each time sequence is matched to its nearest typical representation by utilizing raw profile data, significantly reducing the data points needed for optimization. The emergence of significantly different typical days is expected. For instance, by 2050, Switzerland is anticipated to rely heavily on solar energy, resulting in electricity generation that is highly time-dependent, both daily and seasonally. Simultaneously, a substantial portion of future demand will be driven by heating needs, leading to higher electricity consumption during winter.

As a result, the clustering identifies three main categories: (a) Winter days with low PV generation and high demand; (b) Mid-season days with moderate PV generation and relatively high demand; (c) Summer days with high PV generation (partially curtailed) and lower demand.

These typical days were computed using the KMedoids clustering, a robust unsupervised learning technique favoured for its resilience to outliers and external variables. This method is immune to biases and ensures more accurate profile handling. The optimal number of clusters has been determined by minimizing the mean absolute percentage error (MAPE), as shown in Equation 1. This metric evaluates clustering efficacy by calculating the relative distances of points to their cluster, as detailed in the equation below. The clustering performance of each screened number of clusters can be found in Supplementary Figure A5 for Switzerland's current profile.

$$MAPE = \frac{1}{n}\sum_{i=1}^{n}\left|\frac{C_i^{actual} - C_i^{predicted}}{C_i^{actual}}\right| \times 100$$

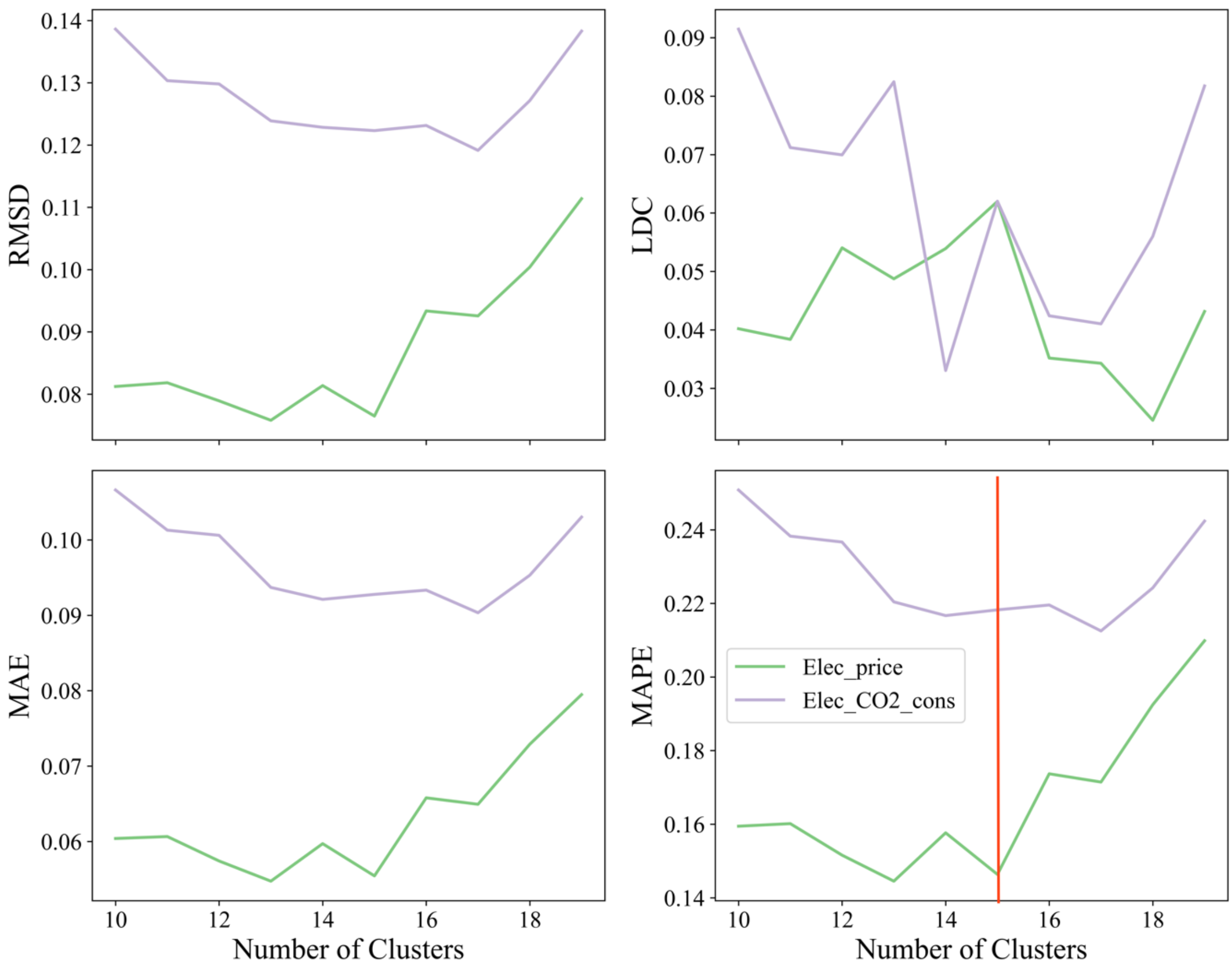

Supplementary Figure A5: Cluster Performance Metrics for Typical Day Selection. Performance metrics as a function of the number of clusters for determining the optimal number of clusters. Example for the current profile of Switzerland.

Supplementary Note B: Results

Supplementary Results S1: SOEC system analysis

Supplementary Table B1 presents the primary material and energy flows for the proposed SOEC system alongside a traditional SOEC system. The significant difference between the two designs lies in integrating the SOEC and methanation process. Both designs' electrolyzer and methanator components share the same flow parameters: stack electricity input of 30 kW, water flow of 0.139 mol/s, and CO_2 flow of 0.03 mol/s. Both designs produce 0.059 mol/s of oxygen and 0.031 mol/s of methane-rich fuel.

In the proposed SOEC system (Main manuscript Figure 4a), an ejector utilizes high-pressure CO_2 from the hybrid tank to increase the pressure of the hydrogen stream from the electrolyzer. After that, the mixed flow is compressed to 15 bar and then heated to 280 ^{0}C. The traditional design (Figure A1) compresses hydrogen to 15 bar, while CO_2 from the hybrid storage tank is heated and expanded from 75 bar to 15 bar. Finally, the mixed flow is cooled before the methanator.

As shown in Supplementary Table B1, the electricity required (consumed by compressors – generated by turbines) in the proposed SOEC system is significantly lower (0.597 kW) compared to the traditional design (1.905 kW). This difference results in a notable efficiency advantage for the proposed design, achieving 77.1% conversion efficiency, which is more than 3% compared to the traditional design. The use of the ejector in the proposed SOEC system also eliminates one heat exchanger.

Detailed information on heat flows can be found in Supplementary Table B2, whereas Table B1 presents the heat cascade outcome. In Supplementary Table B2, steam production (H'1) has been represented by three duties (H'1-1 water heating, H'1-2 evaporation, H'1-3 superheating). Further, the cooling of the hydrogen stream from the electrolyzer (C'1) has been represented by two duties (C'1-1, C'1-2), due to water condensation. Similarly, the methane stream from the methanator (C'3) has been represented by two duties (C'3-1, C'3-2).

As the electrolyzer operates in thermoneutral mode, a small amount of hot utility is required after heat cascade among various hot and cold streams for both the proposed and traditional SOEC systems, provided by an electrical heater consuming 0.363 kW of electricity. The reduced compression requirement in the proposed SOEC system leads to a lower demand for cold utility, which stands at 3.318 kW compared to 4.11 kW in the traditional design. Moreover, this study includes an analysis of waste heat valorization through RC integration. Both systems are capable of generating 1.91 kW of electricity from the waste heat available between 500 and 100 °C. The waste heat conversion into electricity enhances the overall system efficiency, reaching 82.1 and 78.6% for the proposed and traditional designs, respectively. The hot and cold composite curves present minimum energy requirements of a process, whereas grand composite curves are used for utility selection and to evaluate heat recovery potential. Finally, integrated composite curves analyze the integration of a unit (e.g., RC) with the complete system (e.g., SOEC). In the case of RC integration, integrated composite curves also present the electricity production. Supplementary Figure B2(a) presents the integrated composite curve for the SOEC system with and without RC integration. This figure also shows heat generation by the methanation reaction, heat required for the water evaporation, and high-temperature heat supplied by the electric heater.

Supplementary Table B1: Details of materials and energy flows for the proposed and traditional SOEC system designs

	**Proposed SOEC**	**Traditional SOEC**
**Process modelling outcome**		
Stack power input, kW	30	30
Water input, mol/s	0.139	0.139
CO_2 input, mol/s	0.030	0.030
O_2 produced, mol/s	0.059	0.059
Fuel produced, mol/s	0.031	0.031
Water output from stack, mol/s	0.021	0.021
Water output from methanator, mol/s	0.058	0.058
Electrical heater electricity consumption, kW	0.363	0.363
Electricity required (comp. in – turb. Out), kW	0.597	1.905
System conversion efficiency, %	77.1	73.9
**Heat cascade analysis**		
Minimum number of HEX	18	20
Total HEX area, m^2	0.465	0.431
Hot utility, kW	0.363	0.363
Cold utility, kW	3.318	4.11
**Rankine cycle (RC) integration**		
Additional electricity generated by RC, kW	1.914	1.913
System conversion efficiency with RC, %	82.1	78.6

Supplementary Table B2: Heat flows with temperatures for the proposed and traditional SOEC systems

**Proposed SOEC**	**Temperature In (°C)**	**Temperature Out (°C)**	**Duty (kW)**
H’1-1	25	95	0.902
H1’-2	95	102	5.840
H’1-3	102	750	3.414
C’5	750	25	-1.290
C’1-1	750	50	-3.294
C’1-2	50	25	-0.618
H’2	74	280	0.982
Methanator	280.1	280	-5.203
C’3-1	280	178	-0.370
C’3-2	178	25	-3.126
C’4	175	25	-0.207
**Traditional SOEC**	**Temperature In (°C)**	**Temperature Out (°C)**	**Duty (kW)**
H’1-1	25	95	0.902
H’1-2	95	102	5.840
H’1-3	102	750	3.414
C’5	750	25	-1.290
C’1-1	750	50	-3.294
C’1-2	50	25	-0.618
H’2	25	140	0.382
C’6	319	280	-0.192
Methanator	280.1	280	-5.203
C’3-1	280	178	-0.370
C’3-3	178	25	-3.126
C’4	175	25	-0.207

Supplementary Results S2: SOFC system analysis

Supplementary Table B3 presents material and energy flows for the proposed SOFC design alongside three conventional SOFC designs. Although this study does not optimize the operating conditions of the proposed SOFC system, the reported values are close to optimal. The elimination of two heaters, one before the external reformer and another between the external reformer and the stack, is one of the key design goals. This requirement inherently sets a relatively high AOG recirculation ratio. For the proposed and traditional SOFC designs, detailed information on heat flows (temperatures and duties) is presented in Table B4. All SOFC designs use three-stage expansion of fuel and three-stage compression of CO_2. The fuel is heated (duty represented by H1-1, H1-2, H1-3 in Table B4) before each expansion stage, whereas compressed CO_2 is cooled (duty represented by C3-1, C3-2, C3-3 in Table B4) after each compression stage. Further, in Table B4, steam production (H2) has been represented by three duties (H2-1 water heating, H2-2 evaporation, H2-3 superheating). The cooling of combustion products after burner (C2) has been represented by two duties (C2-1, C2-2), due to water condensation.

For the proposed SOFC system, a fresh fuel requirement of 0.02 mol/s is computed, whereas external water flow is set at 0 mol/s, benefiting from AOG recirculation using ejectors. To ensure a fair comparison, the Warm AOG and Hot AOG designs maintain the same fuel flow rate, whereas, for the No AOG design, a slightly higher fuel requirement of 0.021 mol/s is necessary to prevent rapid stack degradation. The No AOG design requires an external water flow of 0.039 mol/s to maintain the minimum S/C ratio. The inlet temperature for the adiabatic external reformer varies across different cases, directly correlating with the recirculation rate. A higher recirculation rate allows a lower inlet temperature because the necessary heat for methane reforming can be supplied with a small temperature difference across the external reformer. Consequently, for the Warm AOG design, the reformer inlet temperature is only 542°C. In contrast, the proposed SOFC design requires an inlet temperature of 603°C, while the Hot AOG and No AOG designs require 617 and 645°C, respectively.

As detailed in Supplementary Table B3, the power output from the stack is about 10 kW for all designs. For the Warm AOG and Hot AOG designs, the AOG blower requires power, whereas in the proposed SOFC design, the AOG blower is not required, reducing the internal power consumption. A notable point is the power consumption/generation by compressors/turbines, which connect the rSOC system to the hybrid storage tank. The proposed SOFC system exhibits a higher net power consumption of 0.337 kW, compared to 0.196 to 0.237 kW for other designs. This increase is reasonable as fewer turbines are used in the proposed SOFC system, resulting in reduced power recovery. However, this is offset by the heat benefits and the power savings by avoiding the AOG blower, as reflected in the electrical efficiency. The electrical efficiency of the proposed SOFC system stands at 63.3%. Although the No AOG design shows slightly higher efficiency at 64.8%, it potentially shows poorer performance in heat availability. Meanwhile, the Warm AOG and Hot AOG design record electrical efficiencies of 61.7 and 54.5%, respectively, lower than that of the proposed SOFC system. This comparative analysis underscores the proposed SOFC system's advantage in terms of electrical efficiency, thermal management and mechanical design.

The proposed SOFC system showcases marked advantages in thermal management, which has been assessed via heat cascade analysis in Supplementary Table B1. The total area of heat exchangers measures 0.142 m^2, which is about half of the Hot AOG designs and one-third of the No AOG and Warm AOG designs. This reduction in heat exchanger area is due to eliminating two heaters before and after the reformer and an effective heat cascade strategy, which necessitates only nine heat exchangers. This streamlined approach simplifies the plant layout, conserving space and reducing extensive piping, lowering the system cost compared to the other three designs. Further, the direct mixing of streams in the proposed design minimizes the cold utility, requiring only 5.28 kW, compared to 7.33, 7.10, and 7.87 kW for No AOG, Warm AOG and Hot AOG designs, respectively. Finally, waste heat valorization has been quantitatively assessed. After including the electricity generated by RC integration, the proposed SOFC system exhibited the highest electricity efficiency (72.6%). Notably, the No AOG design, which previously demonstrated the highest efficiency, has a lower efficiency of 71.3%. Moreover, the efficiencies of Warm AOG and Hot AOG designs are observed at 71 and 65.9%, respectively. Supplementary Figure B2(b) presents the integrated composite curve for the SOFC system with and without RC integration. It can be visualized that all streams, except H1 (in Supplementary Table B4) must cool down for the proposed SOFC system.

Supplementary Table B3: Details of materials and electricity flows for No AOG, Warm AOG, Hot AOG and proposed SOFC designs

	**No AOG**	**Warm AOG**	**Hot AOG**	**Proposed SOFC**
**Process modelling outcome**				
Fuel input, mol/s	0.021	0.020	0.020	0.020
External water input, mol/s	0.039	0.000	0.000	0.000
Reformer inlet temperature, C	646	542	617	603
Reformer operating temperature, C	520	520	520	520
Total recirculation flow rate, mol/s	0.000	0.346	0.287	0.308
Recirculation flow rate to reformer, mol/s	0.000	0.346	0.053	0.067
Recirculation flow rate to stack, mol/s	0.000	0.000	0.228	0.235
Fresh air flow rate, mol/s	1.010	0.896	1.040	1.015
Lean air flow rate, mol/s	0.975	0.861	1.006	0.980
Stack power output, kW	10.763	9.983	10.031	10.013
AOG blower power consumption, kW	0.000	0.381	1.494	0.000
Air blower power consumption, kW	0.212	0.188	0.218	0.213
Net (comp. – turb.) power required, kW	0.232	0.237	0.196	0.337
SOFC system net power output, kW	10.319	9.215	8.122	9.463
Electrical efficiency, %	64.8	61.7	54.4	63.3
O_2 from oxygen buffer, mol/s	0.005	0.003	0.003	0.003
Water flow to water tank, mol/s	0.080	0.038	0.038	0.038
CO_2 supply to hybrid tank, mol/s	0.019	0.019	0.019	0.019
**Heat cascade analysis**				
Minimum number of HEX	21	18	16	9
Total area of HEX, m^2	0.425	0.407	0.262	0.142
Cold utility, kW	7.335	7.099	7.873	5.28
**Rankine cycle (RC) integration**				

Additional electricity generated by RC, kW	1.026	1.394	1.726	1.386
System electrical efficiency with RC, %	71.3	71.0	65.9	72.6

Supplementary Table B4: Heat flows with temperatures for No AOG, Warm AOG, Hot AOG and the proposed SOFC designs

**No AOG**	**Temperature In (°C)**	**Temperature Out (°C)**	**Duty (kW)**
H1-1	25	115	0.073
H1-2	25	115	0.071
H1-3	25	115	0.071
H2-1	25	100	0.224
H2-2	100	106	0.177
H2-3	106	150	1.478
H3	104	646	1.388
H5	520	720	0.593
C1	96	25	-2.019
C2-1	900	95	-5.114
C2-2	95	25	-3.821
C3-1	150	100	-0.040
C3-2	244	100	-0.118
C3-3	243	25	-0.298
**Warm AOG**	**Temperature In (°C)**	**Temperature Out (°C)**	**Duty (kW)**
H1-1	25	115	0.068
H1-2	25	115	0.067
H1-3	25	115	0.067
H3	138	542	5.885
H5	520	720	3.245
C4	800	120	-9.579
C1	92	25	`-1.692
C2-1	900	95	-2.883
C2-2	95	25	-1.836
C3-1	150	100	-0.038
C3-2	244	100	-0.114
C3-3	243	25	-0.289
**Hot AOG**	**Temperature In (°C)**	**Temperature Out (°C)**	**Duty (kW)**
H1-1	25	115	0.068
H1-2	25	115	0.067
H1-3	25	115	0.067
C5	778	720	-0.809
C1	97	25	-2.106
C2-1	900	95	-2.883

C2-2	95	25	-1.836
C3-1	150	100	-0.038
C3-2	244	100	-0.114
C3-3	243	25	-0.289
**Proposed SOFC**	**Temperature In (°C)**	**Temperature Out (°C)**	**Duty (kW)**
H1	25	120	0.072
C1	96	25	-2.032
C2-1	900	95	-2.883
C2-2	95	25	-1.836
C3-1	150	100	-0.038
C3-2	244	100	-0.114
C3-3	243	25	-0.289

Figure B1 visualises the performance of various SOFC designs using six key performance indicators (KPIs), namely, minimum external water flow rate, maximum electrical efficiency, minimum total area of HEX, minimum number of HEX units, minimum cold utility requirements and maximum electrical efficiency with RC integration. These KPIs were selected to reflect the core design priorities of this study, which focuses on system simplification, water self-sufficiency, and thermal integration to enable long-term energy storage via rSOC. While broader challenges such as CO_2 emissions, mode-switching control, and non-steady-state operation are indeed important, they are general concerns inherent to all rSOC systems and not specific to the improved design proposed here. Therefore, they were not included in this comparison, which aims to highlight the specific advancements and trade-offs of the presented configuration. Addressing those broader challenges requires dedicated investigation beyond the scope of this study. The proposed SOFC system, represented by a blue colour line, achieves "Rank 1" in all categories except for electrical efficiency.

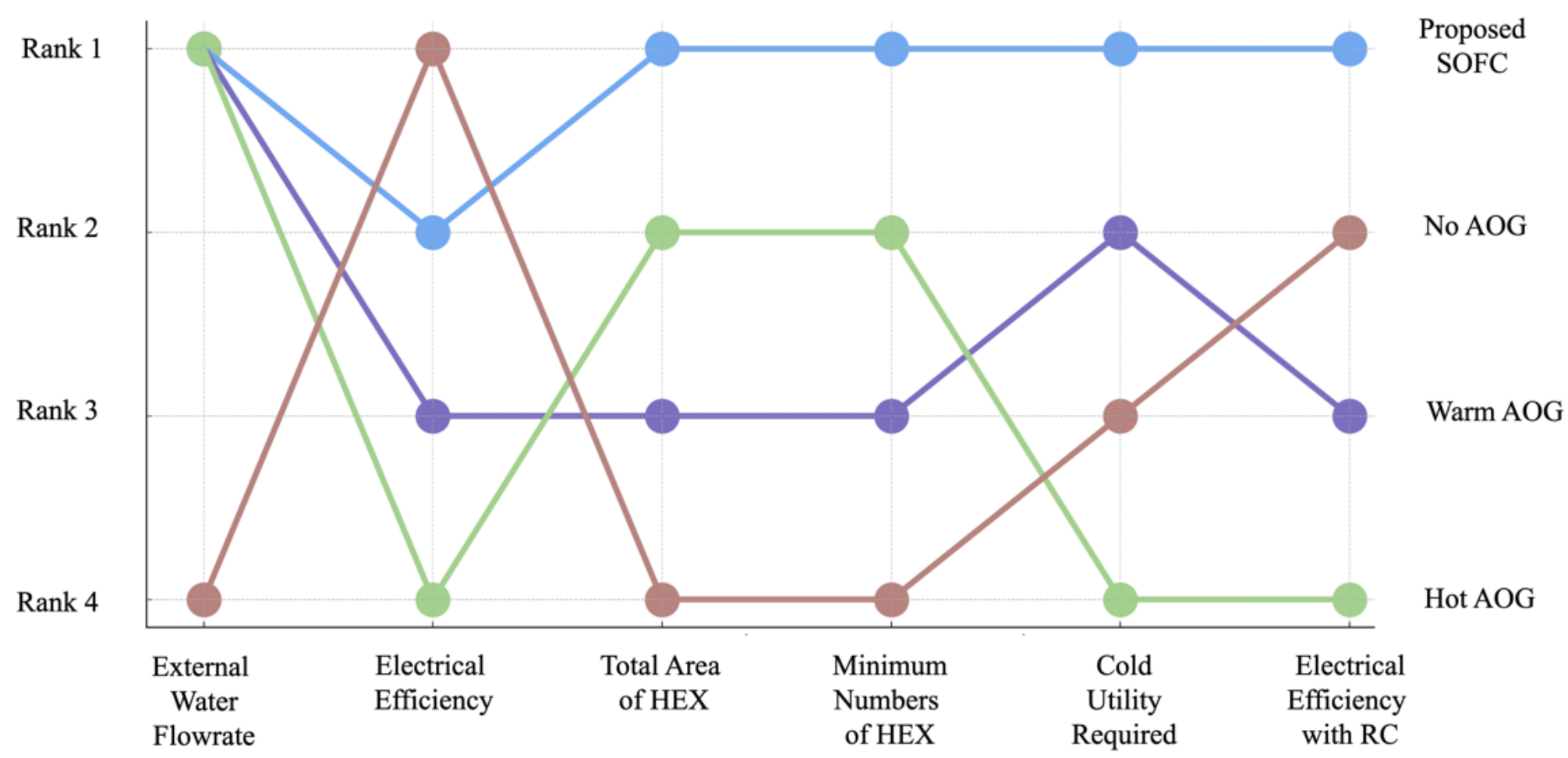

Supplementary Figure B1: **SOFC Design Performance Comparison.** Performance comparison for SOFC designs based on different criteria

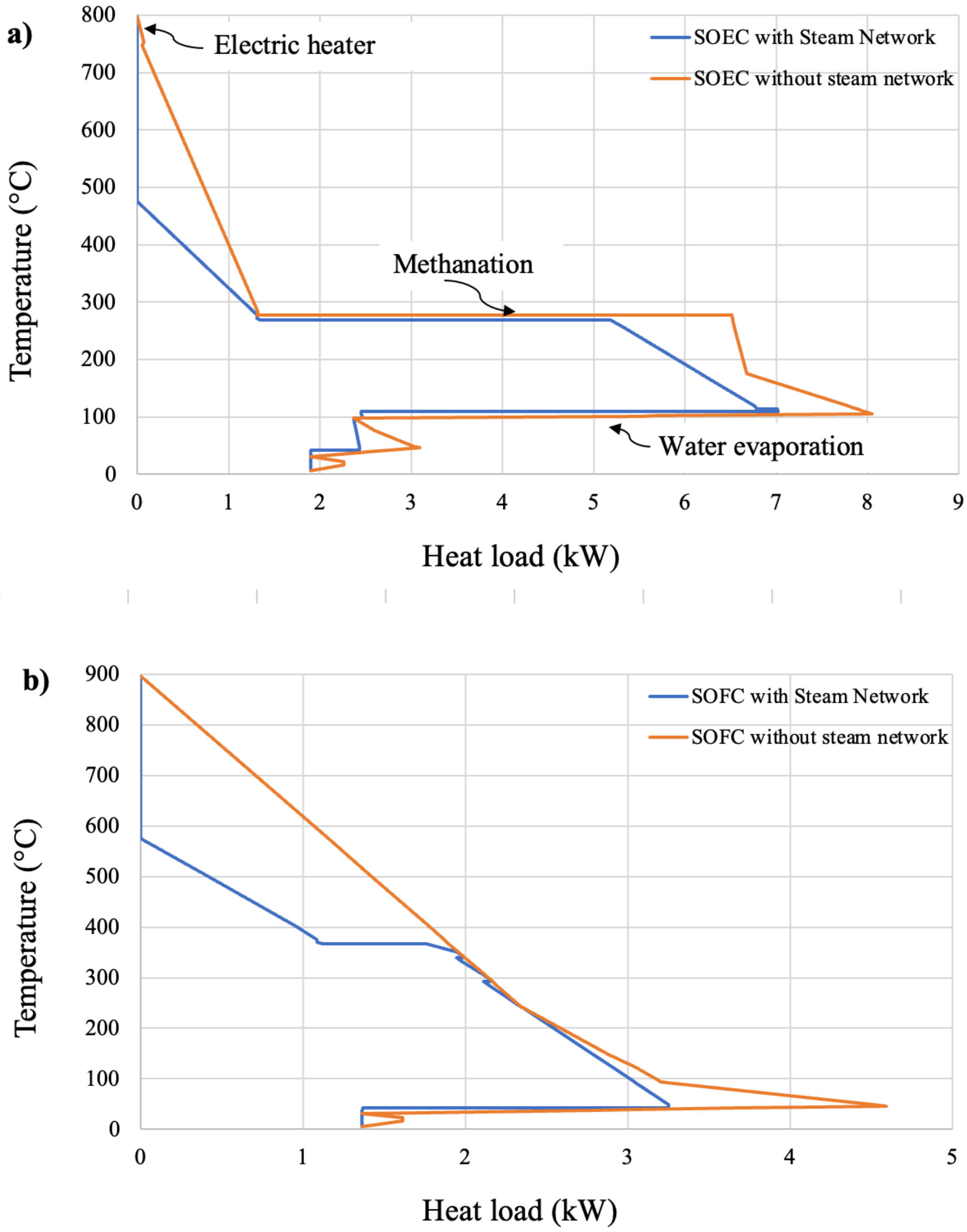

Supplementary Figure B2: **Composite Curves of SOEC and SOFC Systems.** Integrated composite curves of (a) SOEC, and (b) SOFC system

Supplementary Table B5: Details of Pareto solutions shown in Figure 1I

	**A**	**B**	**C**	**D**	**E**	**F**	**G**	**H**	**I**	**J**
OPEX, M€/yr	-0.439	-0.438	-0.424	-0.409	-0.397	-0.383	-0.365	-0.335	-0.317	-0.292
CO_2 emissions, t CO_2-eq/yr	4.33	-14.38	-15.42	-16.35	-17.05	-17.7	-18.38	-19.2	-19.51	-19.66
OPEX + Carbon tax, M€/yr	-0.439	-0.438	-0.424	-0.409	-0.397	-0.383	-0.365	-0.335	-0.317	-0.292
rSOC operational time, h	8760	8760	8490	8204	7948	7661	7293	6699	6339	5839
SOFC/SOEC operational time, -	1.58	1.58	1.59	1.59	1.59	1.58	1.59	1.58	1.58	1.59
Number of mode switch, 1/yr	946	721	827	955	1072	1121	1317	1673	1708	1977
Electricity balance, MWh/yr	-201	-201	-195	-187	-182	-176	-167	-154	-145	-133
O_2 balance, t/yr	110	110	107	103	100	96	92	84	80	73
Storage tank size, m^3	184	248	257	262	254	250	240	227	218	207
Difference (selling – buying) in electricity price, €/MWh	14.74	12.12	12.09	12.48	12.82	13.19	13.58	13.87	14.46	14.93
Difference (selling – buying) in CO_2 intensity, kg CO_2-eq/MWh	75.48	120.61	124.51	128.39	131.84	135.56	140.26	147.88	152.46	158.93
Median operating time SOFC, h	7	7	3	3	2	2	2	2	3	2
Median operating SOEC, h	7	6	5	7	7	7	6	4	4	4

Supplementary Table B6: Results for 21 European countries using rSOC for an average electricity demand of 100 kW. Hybrid tank size was calculated using a volumetric energy density of 760 kWh/m^3 (CH_4 at 75 bar)

Country	Storage capacity, MWh	Storage size, m^3	Total energy output, MWh	Total energy input, MWh	Curtailment, MWh	Number of switches, 1/yr	Discharging duration, h	Charging duration, h	Operating time, h	Maximum power supply, kW	Normalized storage capacity, kWh/kW	Normalized storage size, m^3/kW
AT	62.29	81.97	133.06	224.82	78.89	1045	3389	4453	7842	132.30	470.85	0.62
BE	51.52	67.79	264.08	446.19	307.45	1109	5274	2867	8141	154.18	334.18	0.44
BG	14.33	18.85	96.13	162.42	107.10	960	2914	3868	6782	101.47	141.21	0.19
CH	78.36	103.11	197.57	333.82	186.15	1183	4309	3374	7683	115.32	679.51	0.89
CZ	21.28	28.00	143.42	242.33	172.26	1472	3390	3911	7301	143.68	148.09	0.19
DE	17.39	22.88	127.91	216.12	111.65	922	3584	3062	6646	166.89	104.20	0.14
EE	10.28	13.53	87.69	121.59	253.38	1958	2986	2647	5633	190.77	53.89	0.07
ES	26.03	34.25	80.02	135.20	63.77	2122	3739	2702	6441	61.03	426.50	0.56
FI	22.51	29.62	87.51	147.85	75.89	2208	3347	4322	7669	119.89	187.78	0.25
FR	22.27	29.31	112.02	189.26	75.47	956	3734	3878	7612	113.69	195.93	0.26
GB	16.83	22.14	123.33	208.38	126.67	1709	3725	3875	7600	119.31	141.06	0.19
HU	12.62	16.61	177.59	300.05	253.83	2534	3747	3448	7195	147.00	85.86	0.11
IE	34.19	44.98	151.13	255.35	327.79	498	3115	4015	7130	81.23	420.86	0.55
IT	22.07	29.03	82.72	139.77	55.07	846	2996	4145	7141	127.40	173.21	0.23
LT	8.42	11.07	60.77	102.68	331.49	880	2720	1972	4692	54.84	153.48	0.20
LV	21.61	28.43	165.66	279.89	64.56	1186	4730	3516	8246	109.98	196.47	0.26
NL	31.88	41.95	290.67	491.12	128.01	589	4177	4274	8451	113.38	281.23	0.37
PL	21.26	27.97	200.93	287.40	0.00	702	4669	4091	8760	127.54	166.68	0.22
RO	16.29	21.43	74.29	125.52	177.49	1312	2692	3286	5978	95.96	169.75	0.22
SE	10.44	13.73	34.27	57.91	49.43	1842	2727	3705	6432	47.26	220.84	0.29
SI	60.04	79.01	286.69	459.84	1.88	995	5502	3213	8715	119.57	502.16	0.66

Supplementary Table B7: Results for 21 European countries using the battery for an average electricity demand of 100 kW. Battery size was calculated using a volumetric energy density of 450 kWh/m^3.

Country	Storage capacity, MWh	Storage size, m^3	Total energy output, MWh	Total energy input, MWh	Curtailment, MWh	Number of switches, 1/yr	Discharging duration, h	Charging duration, h	Operating time, h	Maximum power supply, kW	Normalized storage capacity, kWh/kW	Normalized storage size, m^3/kW
AT	154.53	343.39	193.33	240.02	123.96	892	3863	4779	8642	132.30	1168.00	2.60
BE	107.72	239.37	359.57	417.52	431.61	952	5640	3090	8730	154.18	698.66	1.55
BG	16.77	37.27	184.27	210.65	147.01	1005	3718	4502	8220	101.47	165.30	0.37
CH	200.96	446.59	266.96	325.87	263.49	1050	4669	3775	8444	115.32	1742.68	3.87
CZ	39.27	87.27	219.88	254.02	237.03	1365	4104	4248	8352	143.68	273.32	0.61
DE	27.52	61.16	138.64	158.49	180.01	1355	3661	3163	6824	166.89	164.90	0.37
EE	16.97	37.71	173.33	196.75	263.86	2631	3541	4154	7695	190.77	88.96	0.20
ES	33.53	74.50	117.92	136.70	100.18	724	4393	4153	8546	61.03	549.31	1.22
FI	33.70	74.89	127.51	147.03	116.71	1136	3687	4273	7960	119.89	281.10	0.62
FR	34.07	75.72	179.34	205.81	126.25	1063	4135	4466	8601	113.69	299.73	0.67
GB	21.61	48.03	209.07	239.75	181.04	1692	4437	3959	8396	119.31	181.15	0.40
HU	14.48	32.18	244.40	278.42	342.28	1957	4367	3504	7871	147.00	98.51	0.22
IE	67.84	150.77	224.18	265.47	390.73	1257	4697	3853	8550	81.23	835.18	1.86
IT	37.54	83.42	150.09	174.35	87.85	738	3462	5087	8549	127.40	294.67	0.65
LT	17.80	39.56	89.53	103.87	359.05	2884	3340	4385	7725	54.84	324.64	0.72
LV	29.30	65.10	202.97	233.83	147.94	1245	4994	3671	8665	109.98	266.37	0.59
NL	45.68	101.52	321.43	368.35	281.54	736	4809	3348	8157	113.38	402.94	0.90
PL	41.04	91.21	210.47	244.38	52.56	753	4694	4066	8760	127.54	321.81	0.72
RO	26.43	58.73	116.37	136.78	208.32	2561	3138	4201	7339	95.96	275.41	0.61
SE	26.58	59.06	57.16	66.63	63.60	2047	3228	3585	6813	47.26	562.31	1.25
SI	125.87	279.72	304.34	360.43	118.94	1043	5593	2825	8418	119.57	1052.72	2.34

Supplementary Table B8: Economic results for 21 European countries using the rSOC system, the conventional battery and the hybrid system.

Country	rSOC full-charge time, h	rSOC full-discharge time, h	rSOC minimum rated power, kW	Power-based cost, k€/kW		Energy-based cost, k€/kWh		LCOS rSOC only, k€/kWh	LCOS battery only, k€/kWh	LCOS hybrid strategy, k€/kWh		
				rSOC	Battery	rSOC	Battery			rSOC cost	Battery cost	Total cost,
AT	5496	3264	19.1	24.5	141.6	57.2	133.3	2.08	8.98	0.67	0.12	0.79
BE	6251	2509	20.5	23.2	84.7	76.3	133.3	1.15	3.36	0.31	0.07	0.38
BG	3916	4844	3.0	21.3	20.0	166.2	133.3	1.90	1.02	0.19	0.13	0.32
CH	5918	2842	27.6	26.5	211.2	42.8	133.3	1.33	8.45	0.60	0.07	0.67
CZ	7835	925	23.0	21.4	33.1	159.0	133.3	1.81	2.01	0.42	0.12	0.54
DE	7728	1032	16.9	21.0	20.0	221.6	133.3	2.30	2.23	0.36	0.16	0.52
EE	8582	178	57.8	20.5	10.8	418.7	133.3	3.74	1.10	1.30	0.27	1.57
ES	4762	3998	6.5	24.1	66.6	62.0	133.3	1.56	3.19	0.43	0.09	0.53
FI	5768	2992	7.5	21.8	34.1	127.6	133.3	2.52	2.97	0.38	0.17	0.55
FR	7205	1555	14.3	21.9	36.3	122.7	133.3	1.87	2.13	0.41	0.13	0.53
GB	7162	1598	10.5	21.3	22.0	166.4	133.3	1.74	1.16	0.27	0.12	0.39
HU	1908	6852	2.2	20.8	11.9	266.7	133.3	1.45	0.67	0.09	0.10	0.19
IE	5573	3187	10.7	24.0	101.2	62.7	133.3	1.10	3.40	0.33	0.07	0.39
IT	8054	706	31.3	21.6	35.7	137.5	133.3	2.81	2.81	0.92	0.19	1.11
LT	6822	1938	4.3	21.5	39.3	153.8	133.3	1.63	2.23	0.25	0.11	0.36
LV	4523	4237	5.1	21.9	32.3	122.4	133.3	1.23	1.62	0.17	0.08	0.25
NL	4732	4028	7.9	22.7	48.8	88.7	133.3	0.75	1.60	0.15	0.05	0.19
PL	8098	662	32.1	21.6	39.0	142.4	133.3	1.16	2.19	0.38	0.08	0.46
RO	7259	1501	10.9	21.6	33.4	140.1	133.3	2.35	2.55	0.46	0.16	0.62
SE	7704	1056	9.9	22.1	68.2	110.1	133.3	2.58	5.22	0.79	0.17	0.96
SI	3851	4909	12.2	24.8	127.6	54.3	133.3	0.88	4.64	0.26	0.05	0.31

Supplementary Table B9: Sensitivity analysis on the rSOC CAPEX for 21 European countries and its impact on the hybrid strategy LCOS.

Country	Hybrid LCOS Breakdown, €/kWh				Hybrid LCOS Total, €/kWh	
	LCOS Battery	LCOS Storage Tank	LCOS rSOC (10k €/kW)	LCOS rSOC (1k €/kW)	LCOS Total (10k €/kW)	LCOS total (1k €/kW)
AT	0.12	0.41	0.26	0.03	0.79	0.56
BE	0.07	0.17	0.14	0.01	0.38	0.25
BG	0.13	0.13	0.06	0.01	0.32	0.27
CH	0.07	0.35	0.25	0.03	0.67	0.44
CZ	0.12	0.13	0.29	0.03	0.54	0.28
DE	0.16	0.12	0.24	0.02	0.52	0.31
EE	0.27	0.11	1.19	0.12	1.57	0.50
ES	0.09	0.28	0.15	0.01	0.53	0.39
FI	0.17	0.22	0.16	0.02	0.55	0.41
FR	0.13	0.18	0.23	0.02	0.53	0.33
GB	0.12	0.12	0.15	0.02	0.39	0.25
HU	0.10	0.07	0.02	0.00	0.19	0.17
IE	0.07	0.20	0.13	0.01	0.39	0.28
IT	0.19	0.24	0.68	0.07	1.11	0.49
LT	0.11	0.12	0.13	0.01	0.36	0.24
LV	0.08	0.11	0.06	0.01	0.25	0.20
NL	0.05	0.10	0.05	0.00	0.19	0.16
PL	0.08	0.09	0.29	0.03	0.46	0.20
RO	0.16	0.20	0.26	0.03	0.62	0.38
SE	0.17	0.27	0.52	0.05	0.96	0.49
SI	0.05	0.18	0.08	0.01	0.31	0.24

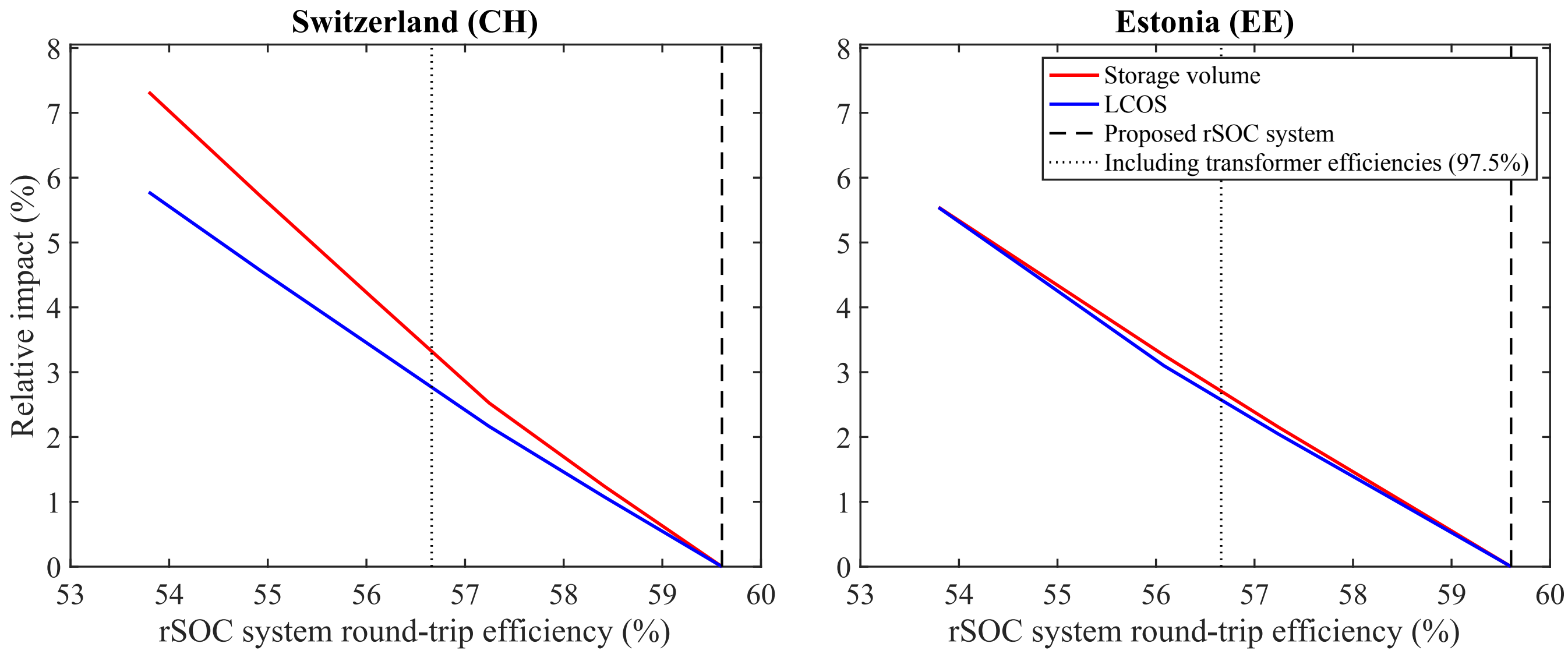

Supplementary Figure B3: **Impact of rSOC Efficiency on Storage Volume and LCOS.** Relative impact of the rSOC system round-trip efficiency (%), including transformer efficiencies, on the storage volume and LCOS: percentage increase compared to the baseline case for the two extreme countries Switzerland and Estonia. Baseline case is shown as dashed line, while dotted line represents a transformer efficiency of 97.5% (in each direction).

Supplementary Table B10: Normalized pumped-hydro storage sizes for six countries

Country	Switzerland	Austria	Spain	Sweden	France	Italy
**Norm. storage, m^3/kW**	775	515	331	249	210	197

Supplementary References

[1] M. Lamagna, D. Groppi, and B. Nastasi, “Reversible solid oxide cells applications to the building sector,” International Journal of Hydrogen Energy, vol. 48, pp. 27033–27058, Aug. 2023.
[2] M. B. Mogensen, M. Chen, H. L. Frandsen, C. Graves, J. B. Hansen, K. V. Hansen, A. Hauch, T. Jacobsen, S. H. Jensen, T. L. Skafte, and X. Sun, “Reversible solid-oxide cells for clean and sustainable energy,” Clean Energy, vol. 3, pp. 175–201, Nov. 2019.
[3] G. Buffo, D. Ferrero, M. Santarelli, and A. Lanzini, “Energy and environmental analysis of a flexible Power-to-X plant based on Reversible Solid Oxide Cells (rSOCs) for an urban district,” Journal of Energy Storage, vol. 29, p. 101314, June 2020.
[4] A. Wu, M. Xiong, Y. Zhang, S. Hanson, J. Wang, W. Guan, and S. C. Singhal, “CO2 utilization by reversible solid oxide cells towards carbon neutralization for long-term energy storage,” Chemical Engineering Journal, vol. 466, p. 143275, June 2023.
[5] E. Reznicek and R. J. Braun, “Techno-economic and off-design analysis of stand-alone, distributed-scale reversible solid oxide cell energy storage systems,” Energy Conversion and Management, vol. 175, pp. 263–277, Nov. 2018.
[6] S. H. Jensen, C. Graves, M. Mogensen, C. Wendel, R. Braun, G. Hughes, Z. Gao, and S. A. Barnett, “Large-scale electricity storage utilizing reversible solid oxide cells combined with underground storage of CO 2 and CH 4,” Energy & Environmental Science, vol. 8, no. 8, pp. 2471–2479, 2015.
[7] C. Wendel, P. Kazempoor, and R. Braun, “Novel electrical energy storage system based on reversible solid oxide cells: System design and operating conditions,” Journal of Power Sources, vol. 276, pp. 133–144, Feb. 2015.
[9] G. Butera, S. H. Jensen, and L. R. Clausen, “A novel system for large-scale storage of electricity as synthetic natural gas using reversible pressurized solid oxide cells,” Energy, vol. 166, pp. 738–754, Jan. 2019.
[9] Z. Zhang, J. Zhou, Z. Zong, Q. Chen, P. Zhang, and K. Wu, “Development and modelling of a novel electricity-hydrogen energy system based on reversible solid oxide cells and power to gas technology,” International Journal of Hydrogen Energy, vol. 44, pp. 28305–28315, Oct. 2019.
