# Supplementary material for: Optimizing reversible solid oxide cell technology for long-term energy storage in Europe’s decarbonized frameworks
Source: Nat Commun. 2026 Jun 25;17:8071. doi: 10.1038/s41467-026-74229-z (PMC13454186; doi:10.1038/s41467-026-74229-z)
Supplement: Supplementary file 2 [file 41467_2026_74229_MOESM2_ESM.pdf]

nature portfolio

Peer Review File

Optimizing Reversible Solid Oxide Cell Technology for Long-Term Energy Storage in Europe's Decarbonized Frameworks

Corresponding Author: Dr Xinyi Wei

This file contains all reviewer reports in order by version, followed by all author rebuttals in order by version.

Version 0:

Reviewer comments:

Reviewer #1

(Remarks to the Author)
The manuscript is addressing a cutting-edge topic of using the reversible solid oxide cell (rSOC) technology for long duration energy storage. The two most important findings are that a hybrid approach is likely to be the most economical for most of the analyzed countries, and that rSOC for energy storage is a viable technology solution already today. These findings are noteworthy and important to disseminate. The constraints and operational conditions implemented on modeling the rSOC system are reasonable and in line with technical capabilities.

However, minor adjustments are recommended before publication.

1) The authors have chosen to refer to storage costs with the unit of k€/kW_max,supply, but a more fitting unit for energy storage capacity may be k€/kWh.

2) Another unit chosen is m3 for storage capacity. While this unit addresses the volume required, this reviewer would recommend expanding the discussion. First of all, the m3-number is less relevant as you would be unlikely to store the energy in one huge container (unless underground caverns are used). Something more tangible would be storing the energy in shipping containers, but those have a height of ~2.6 m, so the footprint would increase significantly compared to what one might think only considering the m3 number. As an example, 123 m3 covered by shipping containers would require a footprint of almost 2000 m2. Granted, several containers could be stacked on top of each other. Secondly, while the energy density is defined in the SI, it should be included, if even as a brief note, in the main manuscript. As the authors are aware, the energy density changes quite significantly for different gases, temperatures, and pressures, and similar for Li-ion battery technologies/generations. For instance, it would be interesting to consider and discuss how the conclusions would change if the CH4 was stored at 200-250 bar, instead of 75 bar.

For these reasons, this reviewer recommends changing to kWh as the unit for energy capacity, which is more generic. A section on space requirements with the m3-unit could be added then. Similar for m3/kW in Fig 2 - using kWh/kW or simply hours, is a good, tangible unit for storage capacity needed. If the authors want to maintain the m3-unit, this reviewer suggests adding more discussion around these issues.

3) While well explained in the Methods and/or SI, the main manuscript lacks a couple of definitions and details, which this reviewer thinks should be provided in the main manuscript to deliver a full and cohesive story. Examples include the initial description of a rSOC and the terms "no-anode off-gas (AOG)" and "hot-AOG".

4) In the analysis of demand, did you account for odd years with e.g. very cold winters? Or are the annual profiles identical? If you don't account for rare weather circumstances, you may be underestimating your storage requirements.

5) It is stated that the rSOC has zero self-discharge, but in the profiles "hot standby" is used. Presumably this would require discharging the battery slightly. Has this been accounted for?

6) The authors label some of the countries as needing "short-term storage behaviour" rather than long-term, but the required energy storage to cover the full year is still 100s of hours. By any current definition, that is long duration.

More detailed comments are included in the attached annotated manuscript.

Reviewer #2

(Remarks to the Author)
This paper demonstrates an rSOC system design optimized based on atmospheric pressure operation, ejector, and a hybrid methane-CO_2 tank, with the aim of enabling compatibility with existing energy infrastructures. In addition, the authors provide energy storage strategy guidelines to individual European countries. The following are my comments. I hope that the comments will contribute to improving the manuscript.

- Major purpose of the proposed system appears to be the application of ejectors and the hybrid tank. However, several previous studies have already reported anode gas recirculation using ejectors. Moreover, the performance improvement resulting from the hybrid tank configuration is not clearly highlighted in this work. The authors should clarify the novel contributions of the ejector compared to prior literatures and explain the performance differences between systems with and without the hybrid tank.

- System modification seems critical in this study, but all relevant schematics and layouts are presented only in the supplementary figures rather than in the main text. Why?
- In Figure 1, on what basis were Solutions A-J selected?
- The key advantage seems to be the reduction of two heat exchangers via ejector integration. However, what are the potential trade-offs or technical issues that may arise from this simplification?
- Is the integration of ejectors and hybrid tanks feasible in practical systems? Are there any experimental precedents?
- The relationship between the proposed system design and the long-term and short-term energy storage strategies remains unclear. The two seem to be largely independent-could the authors clarify this?
- In Figure B1, are the six KPIs selected based on a standardized framework? How about fuel utilization and CO_2 emissions? I think that six KPIs appear to emphasize the benefits of ejector integration, yet do not reflect its limitations-such as parasitic energy losses, control challenges during mode switching, or operational instability under non-steady-state conditions.
- On page 13, line 272, Spain is reported to have a wind energy share of 0.8, yet it is classified as dark green in Figure 2f. What is the quantitative basis for the color classification?
- In Figure 2f, what is the correlation between the number of mode switches (SN)/normalized storage volume (V) and the trend as the long-/short-term storage characteristics?
- On page 13, line 281, the manuscript states that rSOC and batteries can be co-located with renewable sources for distributed storage, but pumped-hydro storage is widely known to be site-constrained. Is a direct comparison between rSOC and pumped hydro appropriate?
- The thermal energy input required to maintain 750°C operation in rSOC is not clearly accounted for in the cost analysis. Would this omission significantly affect the viability or cost-competitiveness of the proposed system?
- In Table B1 of the SI, why does the total HEX area increase in the proposed SOEC design?
- The visual roughness in Figures 2c and 2e, as well as the comparative storage trends shown in Figures 2 and 3, would be more interpretable with the inclusion of quantitative metrics or statistical summaries.
- In Table B2, why does the temperature rise downstream of C"2?
- Please specify the data sources for Figures 2 and 3 more clearly.

Reviewer #3

(Remarks to the Author)
In this paper, authors discuss the indispensable roles of both long-term and short-term energy storage solutions, and share some perspectives on Europe's future energy framework. Based on the respective advantages and disadvantages of rSOC technology and battery system, the authors propose a hybrid energy storage strategy, which looks feasible for now. Different with regular research articles, this manuscript adopts a broader perspective: focusing on the future energy framework, which can offer meaningful guidance for numerous research scholars and government sectors. Although this study has good innovation and practical potential, there are some issues need to be resolved.
1. This is curious: the main text only includes four figures, yet the authors reference "Figure 6". Where are Figure 4 and Figure 5?
2. As we know, the energy costs are influenced by many factors, such as differences between seasons, climates, and regional environments. Therefore, have the cost data provided in the paper considered these factors? Are the data sources reliable?
3. The authors primarily discuss Europe's energy framework in the paper. What about other continents? could the research results and conclusions be applied to them? It is recommended that the authors conduct an appropriate analysis of energy frameworks in other regions as well, the Asian region for example.
4. The text labels in some figures (e.g., Figure 6) are too small to be clearly visible. Please revise this issue.

Reviewer #4

(Remarks to the Author)
The submitted manuscipt involves an interesting investigation on using rSOC as storage system, with clustered annual

modelling of hourly operation for optimization under different conditions. The analysis expands to assess the competition and/or integration with other storage technologies. The topic is of great current interest. The study appears well-supported from the methodological point of view in the optimization part, and results are consistent. On the opposite, the proposed process modelling of the rSOC system does not appear sufficiently accurate nor the simplifications are acknowledged. Also, the narrative shall be improved. Overall, the authors try to combine process simulation (of which, only efficiencies are mentioned in the main text) and system optimization (more extensively explored) and leave out many details of the core technology. At the same time, the authors try to fit an excessive amount of information in a short document, with many descriptive areas that do not sufficiently convey strong messages. The overall message (hybrid storage systems are effective) is clear. But several intermediate steps seem inconclusive. The neglect of capex in favour of focusing on opex is also a critical aspect when extending suitability assessment and comparison with other technologies.

In the following, punctual comments are provided.

Abstract
- The authors mention (line 22) a sizing of "0.26 to 4.47 m3/kW_avg,demand", which is unclear: m3 of what? what is "avg,demand"?
- The sentence (line 23) "The rSOC technology offers a storage cost of 25-35 k€/kWmax, supply in certain regions, whereas the battery system shows storage costs can be lower than 25 k€/kWmax,supply in others". In general, storage cannot be defined merely in terms of kW, since the energy capacity (kWh) is also relevant. In batteries, energy and power capacities are not independent. In rSOC, they are. The value for rSOC investment cost of 25-35 k€/kW seems immensely high, given that SOFC are listed in the range of 2-5 k€/kWmax.

Introduction
- The first sentence of the Introduction may be misleading: climate change is mostly driven by CO2 emissions, which are not technically a "pollutant" but a "climate altering gas".
- Lines 38-41, it may be beneficial to define short- and long-term.
- Line 47, "flat geographical areas" are mentioned and their definition is not clear.
- Line 49, other projects have also addressed and tested rSOC technology, please extend your review.
- Line 58, the presence of a methane-CO2 streams is mentioned for the first time, which was not introduced (the reader may think of co-electrolysis or methanation, with many doubts and no discussion).

Results
- An image of the system scheme under analysis would be appreciated at the beginning of the Results section. Or at least referring to the image in the Methods section.
- The need for and the integration of a Rankine Cycle is not clear.
- The definition of "conversion efficiency" at line 77-78 is unclear (e.g., is that electricity-to-LHV?).
- In Figures A1 and A2 in SI, electricity seems to be transferred from the grid to the rSOC stack. How is power electronics considered? This deserves some discussion.
- Line 81, it is not clear what the "cold utility demand" refers to.
- Information on the electrochemical assumptions is essential (thermoneutral voltage? ASR taken from where? 0D or 1D modelling?)
- The rationale behind the (reasonable) choice of the ratio 1:3 for SOFC and SOEC nominal power is not expressed.
- The SI unit for "metric tonne" is "t".
- Lines 127-130: it is not clear whether the thresholds at 130 and 200 gCO2/MWhe are input values or results.
- Fonts in Figure 1 are too small.
- Line 140, incorporating O2 sales is interesting, but the use of a pure O2 stream or the separation of O2 from the enriched air are not straightfoward and need discussion. The sentence in line 147 is weak.
- Line 143 talks about "maximising economic returns", but the results are presented as "min CO2" optimization.
- Line 162 mentions "CO2 increase". In general, the benchmark of the overall analysis is not clear.
- Lines 160-174 are strictly descriptive, there is no clear message.
- Lines 205-220, the need for this long section is not evident.
- Lines 227-228: what is an "average electricity demand of 1 kW"? Is that the peak or the constant power output? If average, what is the actual profile and/or the max deviation?
- Lines 232-234: is the ratio computed on solar+wind or including all RES (including geothermal and hydro)? what about biomass? referred to which year?
- The "hybrid tank for fuel and CO2 storage" is never really presented. How does it work? How does it separate the fluids?

Methods
- Line 428, "thermoneutral" is common in the SOEC field, rather than "thermal-neutral".
- Line 429, the presence of pure O2 vs sweep air vs sweep O2 is not discussed and this is a shortcoming in supporting the resulting values.
- Line 431-433 (same present also in SI), the sentence "This system utilizes an ejector to reduce CO2 pressure instead of the traditional turbine, with high-pressure CO2 from the hybrid tank acting as the motive gas and atmospheric-pressure hydrogen as the flare gas." is not clear: an ejector is typically used to increase pressure, not reduce.- Capex of rSOC at 10 k€/kW seems excessively high, especially for future scenarios.
- The use of electricity for the entire preheating (incl. evaporation) of water before the SOEC stack makes it hard to believe the high conversion efficiency obtained, withouth thermal recovery from cathode outlet.
- The valorization of waste heat from the rSOC to drive a Rankine Cycle is hard to believe, since available temperature of the streams after thermal integration for the rSOC itself is below 300°C. Needs more info and support.

- What is sigma in Eq. 1 (line 121)?
- Line 533, 10% unconverted fuel means 90% utilization factor, which is extremely high. How is that possible?
- Overall, it seems that the rSOC modelling is veyr simple / approximate. If that cannot be improved, make sure to highlight the assumptions and the limitations.

Supplementary Information
- The submitted support material is almost like a second article. The section "novelties" is unusual there, since what discussed in the main document should be sufficient. This document should provide additional info, such as technological details, complete input and output data, not elements that are essential to understand the main text. As observed above, there are also repetions from the Methods section, which is undesirable.
- Figure A4 in SI, it is curious that clustering leads to typical days with either low demand and high generation or high demand and low generation and no comparable demand and generation case. Please discuss.
- Clustering method (k-means, k-medoids, ierarchical, k-milp, ...) is not described. List of time-varying quantities included in clustering is not presented.

Version 1:

Reviewer comments:

Reviewer #1

(Remarks to the Author)
Thank you to the authors for addressing the comments put forth earlier. Changes are acceptable by this reviewer.

However, this reviewer does have one major and one minor comment left.

This reviewer is in agreement with Reviewer #4 regarding the high estimated cost of the RSOC system. While the cost may be true today for a relatively small demonstration system, like the 50/150 kW system used as an example, it will be far lower in the future. I am familiar with a group who have performed a very detailed (unpublished) economic study of this topic, taking economy of scale and a reasonable learning rate into account, and the estimated Euro/kW and Euro/kWh is far lower. I understand that the authors do not wish to focus on absolute cost values as this is dependent on many parameters, while the efficiency is more intrinsic to the technology. However, since the absolute cost estimates are mentioned in the abstract when discussing Europe's 2050 energy landscape, I would suggest to try to consider economy of scale of all components and a learning rate for the SOC technology, to extend the cost range lower, if that happens to be the finding. This is very important. With some luck, policy makers, investors and technology developers may base their decisions, in part, on publications such as this one, and the projected costs may influence funding of rSOC systems as opposed to other energy storage systems.

One other minor comment:

On page 25-26, line 598-599, it is mentioned that heat can be generated in both SOEC and SOFC mode, and while this is true, stacks in SOEC mode are almost always operated at the thermoneutral potential (as also recommended by the chosen stack vendor in this study). So stacks in SOEC mode would not be generating heat. However, if the authors are thinking about the entire system, then it could be true that heat can be generated in charge mode (SOEC), since the methane synthesis is exothermic. If so, this should be rephrased as such. Also, on page 7, line 150-151, the authors write that SOEC operation requires external heat to maintain thermoneutrality. This seems like a contradiction to the section above. Assuming you operate at thermoneutral voltage, there would be no need for external heat for the SOEC alone. This is possibly just a question of rephrasing these two statements to avoid confusion or misunderstandings.

Reviewer #2

(Remarks to the Author)
The authors' revised manuscript sufficiently addresses many of my previous concerns. However, some issues remain, and I believe that additional revisions would make the work more accessible and beneficial to a broader readership

1. Figures 4 and 5 are still difficult to interpret. The image quality should be further improved and presented in a more visually accessible manner.
2. It appears that Figures 2c and 2e were derived from Figures 2b and 2d, respectively, but the method of derivation is not clearly explained. Additional clarification is required.
3. In Figure 2b, the energy production graph shows a pronounced central peak across most of the 15 regions. Why does this occur, and why is a similar trend not observed in Figure 2d?
4. Since Figures 2 and 3 represent the major contribution of this manuscript, it would strengthen the novelty of the work if the authors could clearly distinguish which datasets were obtained from references and which were generated/simulated through their own analysis.

Reviewer #3

(Remarks to the Author)
The author's revisions to the manuscript are appropriate and adequately address the reviewer's comments. Thus, the revised manuscript can be accepted.

Version 2:

Reviewer comments:

Reviewer #1

(Remarks to the Author)
Thank you to the authors for addressing this reviewers comment on cost, and the minor comments on clear language around thermal management.

One minor comment, which I believe is just an oversigt; Figure 3e, I believe the symbols for 1 k€/kW and 10 k€/kW should be switched, so that a lower rSOC CAPEX leads to a lower overall LCOS.

Beyond that, this reviewer has no further comments.

Reviewer #2

(Remarks to the Author)
The authors have clearly addressed the key concerns, and the revised manuscript is now strengthened in terms of clarity. Therefore, I recommend acceptance for publication in this journal.

Reviewer #5

(Remarks to the Author)
I have read the paper and find the presented results to be of great interest and relevance. However, further work could be done to better clarify and detail certain modelling assumptions, plant layouts, and results of the process analysis.
The authors have addressed all concerns raised by the original Reviewer #4. However, I believe some of the points have not been fully resolved. Below you will find point-by-point responses to the comments that, in my view, did not adequately address the original concerns of Reviewer #4.
In addition, two new comments of my own are included at the end.

General

#1 – Regarding the simplistic process modelling, I concur with the original Reviewer #4. Much of the process modelling appears to draw from previous works, although some of the cited references in this regard do not seem directly relevant to the topic: these should be reviewed carefully. In any case, whenever new system features are introduced, as appears to be the case here, the process description should always be clear to the reader and all assumptions should be made explicit for reproducibility purposes. See the detailed suggestions in the subsequent comments.
The authors compare their novel rSOC concept against a benchmark/conventional rSOC concept, both in the main text and in the supplementary material. The authors should present the layout scheme of the reference concept, so that the introduced novelties are made clearer to the reader.
The CAPEX comment has been resolved. However, it is not clear to me what the reference capacity for the rSOC CAPEX is. Does the value of 10 k€/kW refer to the SOFC or to the SOEC capacity? Given the 3:1 power ratio between the two modes, this distinction has a significant impact on the result.

#2 and #3 - I agree that the main strength of the paper is the system optimization, which indeed deserves more attention. However, it is also true that the optimization results rely heavily on some of the process modelling outcomes, so it is appropriate to dedicate some space to this in the main paper.
That said, in the current version the presentation is, in my opinion, overly descriptive and does not convey strong, clear messages. The text contains numerous numbers relating to component power consumption, heat exchanger areas, and so on, which are difficult to digest without a reference point or broader context. In the first results section, I would recommend focusing more on relative improvements with respect to the benchmark (e.g. percentage increases or decreases), explaining the main drivers behind these improvements, how they impact system efficiency, and how they relate to the distinguishing features of the novel system concept.
Finally, while I understand that the two parts of the paper are connected, this link is not entirely clear to me. Specifically, how is the information on rSOC performance used in the optimization part? Is the roundtrip efficiency used as a key input for the optimization? And given the inherently flexible nature of the rSOC system, how are part-load performances accounted for? I would ask the authors to better clarify the exchange of information between the two parts of the paper. If part-load performances have not been estimated, the reasons for this should be discussed, along with the potential impact on the results of the analysis.

Introduction

#9 – Not addressed completely. A brief mention of other relevant projects, as suggested by the original Reviewer #4, would be expected.

Results

#13 – For better clarity and reading flow, a short description of what conversion efficiency encompasses should be added in the main results section when first introducing the related results.

#14 – The raised point has been addressed in the SI. However, I do not agree on the “relatively minor impact on overall system efficiency” point, as a loss of 5% in one direction of the conversion is magnified when computing roundtrip loss (up to 9-10% impact). Therefore, due to the relevance of this, this should be made more explicit. For example, this information can be added when describing the conversion efficiency (see comment #13). In the SI, it can be made more explicit when detailing SOFC/SOEC mode results in the text, and in Tables B1 and B2, by adding proper subscripts (such as kW_DC). More importantly, the impact of adding power electronics conversion should be discussed when presenting the results, by performing additional calculations, and compare it with main results, which do not include this relevant loss. To avoid excessive additional work at this stage of the revision, this can be done for two extreme cases (two locations of choice).

#15 – Addressed, but the purpose of the cooling water should be specified.

#16 – I could not find any mention of the description of the 0D model, neither in the main paper nor the SI. The authors only briefly mention the Aspen Plus model for the system. However, no details are given about how the rSOC cell/stack is modelled. The authors say in the response to this comment that they developed the rSOC model for this study, so some description of how it is done is required in this study. The authors are encouraged to expand on this and provide more electrochemical data, such as the ASR raised by original Reviewer #4. Within the detailed results of the SI, notably, there is no instance of operating voltage in both modes, which is a key parameter to understand performances. A figure in the SI showing validation of polarization curves model against experimental data could be of aid.

#17 – As suggested in comment #16, nominal operating voltages for both modes should be provided.

#20 – Fonts are still too small, in my opinion.

#23 – The benchmark is still not clear to me. What does “no further use of electricity” mean?. I strongly suggest the authors to declare clearly the benchmark when they introduce the analysis. In particular, the benchmark is still not clear for results in Figure 1b and 1c.

#24 – Addressed, although I have one suggestion to improve clarity: rather than focusing solely on absolute values, the authors can emphasize this by adding % of OPEX increase vs. CO_2 emissions decrease. In their discussion of case G vs. J, for example, decreasing 7% CO_2 emissions require 20% OPEX increase.

#26 – This should be specified in the main text as well.

#27 – A brief introduction to the concept of the hybrid tank should be provided before presenting the related results, for improved clarity and flow. For further details, readers can still be referred to the Methods section.

Methods

#31 – It is still very difficult to me to understand the process, especially for the SOEC system. The layout is too simplistic and does not show neither all flows nor all heat interactions. I can understand that some of the steam feed is produced via heat recovery from methanation, but I could not find any description of this in the text. The authors only write in Methods section: “heating water to 750°C through sequential heating, evaporation, and superheating (H'1) before entering the cathode side of the stack”. And information about this is even scarcer in the SI. For example, what happens to water from 25°C to 750°C? Tables B2 for example mentions some streams that are not present in the presented layout (for example, what is H’3?).
I strongly suggest the authors to provide more detailed process layouts and descriptions. I see that there are many heat exchangers and the process integration is complex, but at least the readers should be able to understand the following key points:
1- What are the main heat transfer interactions within the process?
2- If electric heaters are used to complete evaporation and superheating in SOEC mode, to what extent?
3- What is the amount of heat that is left available for downstream processes? At what temperature and pressure?

#32 – Partly related to my answer to comment #31: I see that TQ composite curves have been added, but they are really confusing and unclear to me. Authors should add more descriptions to the plots, highlighting major streams, contributions of utilities and other components if present (e.g., in the plot, where is the contribution of the the methanator?)

#34 – This value does indeed appear too high. It should be clarified whether it refers to the global utilization factor or to the per-pass utilization within the stack — if the latter, the concern stands. Furthermore, references 12, 31, 43, 44 do not appear to be directly linked to SolydEra. If specifications were provided by SolydEra, this should be stated explicitly in the text

#35 – I think there is something off with some the cited references (see also comment #34). The authors cite Ref. 25 when talking about modelling and validation of the SOEC BoP model, but Ref. 25 is titled “Effect of oxygen partial pressure on the electrochemical impedance of $La_{0.8}Sr_{0.2}MnO_{3-\delta}/Zr_{0.92}Y_{0.08}O_2$ porous composite anodes in solid oxide electrolysis cell” and seems related to experiments at cell level. This and other references should be checked carefully.

Additional comments

#39 - I think that the symbols in Figure 13 the (triangle and diamond) are inverted for 1k and 10k rSOC Capex.

#40 – In the last version of the paper, the authors have introduced the concept of levelized cost of storage (LCOS) as an important KPI to better capture the potential benefits of using rSOC systems for long term storage. They provide details about how it is computed in the supplementary information, both for single technology and hybrid use of battery and rSOC. The authors should briefly describe how it is computed also in the main text, right before introducing the results. This must be done especially for the hybrid case, where some assumptions about rSOC and battery capacities are made.
Moreover, I noticed that they include only CAPEX. Shouldn't it include also operation and maintenance costs? Usually these costs are in the range of 2-3% of the total CAPEX per year, lower than the annual fraction of CAPEX, but I would not say negligible.

Version 3:

Reviewer comments:

Reviewer #5

(Remarks to the Author)
I thank the authors for carefully and thoroughly addressing all comments raised by myself and Reviewer #4.

One minor remark remains, which I believe to be an unintentional typo: on page 30 of the manuscript, the numerator of Equation 2 should read P_SOFC – P_AUX.

Beyond this, I have no further comments and recommend acceptance for publication in this journal.

Open Access This Peer Review File is licensed under a Creative Commons Attribution 4.0 International License, which permits use, sharing, adaptation, distribution and reproduction in any medium or format, as long as you give appropriate credit to the original author(s) and the source, provide a link to the Creative Commons license, and indicate if changes were made.
In cases where reviewers are anonymous, credit should be given to 'Anonymous Referee' and the source.
The images or other third party material in this Peer Review File are included in the article's Creative Commons license, unless indicated otherwise in a credit line to the material. If material is not included in the article's Creative Commons license and your intended use is not permitted by statutory regulation or exceeds the permitted use, you will need to obtain permission directly from the copyright holder.
To view a copy of this license, visit https://creativecommons.org/licenses/by/4.0/

Optimizing Reversible Solid Oxide Cell Technology for Long-Term Energy Storage in Europe's Decarbonized Frameworks

Xinyi Wei, Arthur Waeber, Shivom Sharma, Jan Van herle, Francois Marechal
Corresponding authors: xinyi.wei@epfl.ch; jan.vanherle@epfl.ch; francois.marechal@epfl.ch

Dear Editor and Reviewers,

We would like to sincerely thank you for taking the time to review our manuscript. Your insightful and constructive feedback has been invaluable in improving the quality of our work and enhancing its relevance to the Nature Communications readership.

In response to your comments, we have carefully revised the manuscript and addressed each point raised by the Editor and Reviewers. Regarding the Editor's suggestion on the title, we fully agree that the proposed version is more straightforward. We have implemented the change directly in the revised manuscript, and thus it is not reiterated in this document. For Reviewer 1, we have also included comments from the annotated PDF, though many overlap with those provided via email. We deeply appreciate all the reviewers' feedback, which helped us significantly strengthen the manuscript.

While the key findings and conclusions remain unchanged, supported by the positive feedback from all reviewers, the revisions have further improved the clarity, rigor, and potential impact of the study. All page and line references in this document correspond to the revised manuscript.

Once again, we are grateful for the opportunity to improve our work and hope that the revised version meets your expectations. Thank you for your thoughtful review and consideration.

Sincerely Yours,

Xinyi Wei, Arthur Waeber, Shivom Sharma, Jan Van Herle, Francois Marechal

Short Highlights to Reviewer 1:

Thank you very much for your thorough review and insightful suggestions. Your comments covered all key aspects of the manuscript, from the introduction and methodology to the presentation of results and have been fully addressed in the revised version.

We would like to especially thank you for highlighting the importance of selecting consistent units for storage and cost. We fully agree with your recommendation and have revised the manuscript accordingly to reflect standardized units throughout.

All detailed responses are provided below.

Thank you again for your expert feedback and valuable support.

The following Table addresses the 1st Revision comments from Reviewer 1.

**Overall comments**		
**No.**	**Comments**	**Corrections and Responses**
1	The authors have chosen to refer to storage costs with the unit of k€/kW_max,supply, but a more fitting unit for energy storage capacity may be k€/kWh.	Thank you for your valuable comment regarding the selected unit of energy storage.<br><br>Initially, our intention was to consider the perspectives of all stakeholders, including stack manufacturers and industrial partners. However, during this revision, we recognized that adopting a standard energy storage unit would better serve the broader audience. Therefore, we have updated all economic-related units to €/kWh, including the Levelized Cost of Storage (LCOS). This metric captures round-trip efficiency, maximum energy capacity, number of storage cycles, and power requirements in a single economic value. We believe this change, inspired by your comment, will enhance the relevance and clarity of the manuscript for readers focused on energy storage systems.<br><br>While all numerical values have been revised accordingly, the core message and methodology remain consistent with the original submission. These updates improve the communication of our findings without affecting their substance.<br><br>More information on the main changes related to the economic units can be found in the abstract (page 2, lines 24-26), the main text (pages 17-18, lines 390-402), and Supplementary Information Table B9.<br><br>Other changes to units not directly related to the economic analysis appear

		on page 9 (Figures 1b and 1d), page 13 (line 327), page 14 (line 350), and pages 15-16 (lines 355-363).
2	Another unit chosen is m^3 for storage capacity. While this unit addresses the volume required, this reviewer would recommend expanding the discussion. First of all, the m^3-number is less relevant as you would be unlikely to store the energy in one huge container (unless underground caverns are used). Something more tangible would be storing the energy in shipping containers, but those have a height of ~2.6 m, so the footprint would increase significantly compared to what one might think only considering the m^3 number. As an example, 123 m^3 covered by shipping containers would require a footprint of almost 2000 m^2. Granted, several containers could be stacked on top of each other.<br><br>Secondly, while the energy density is defined in the SI, it should be included, if even as a brief note, in the main manuscript. As the authors are aware, the energy density changes quite significantly for different gases, temperatures, and pressures, and similar for Li-ion battery technologies/generations. For instance, it would be interesting to consider and discuss how the conclusions would change if the CH4 was stored at 200-250 bar, instead of 75 bar.<br><br>For these reasons, this reviewer recommends changing to kWh as	Similar to our response to Comment 1, we believe that converting all units to a standardized format is an important and valuable improvement. Using a unit based on storage capacity (kWh), rather than storage volume (m^3), ensures consistency throughout the manuscript and avoids confusion caused by dependencies on temperature, pressure, or other external parameters. This approach allows the results to reflect only the characteristics of the storage technology itself. Accordingly, in this revision, we have presented all results using kWh as the main unit.<br><br>That said, we appreciate your suggestion to provide volume-related insights to help readers, particularly industrial partners, better visualize the physical space requirements of energy storage. To address this, we have included a rough estimation of the number of containers needed. However, we decided not to include footprint area (in m^2), since storage tanks could be stacked vertically, and estimating total area would require additional assumptions. By providing storage volume instead, readers can adapt the spatial layout based on their specific site conditions.<br><br>Regarding energy density, we have clarified this in the main text (Method section, Table 1, page 28), where we use 75 bar as the baseline storage pressure. As this is just above the critical pressure of CO_2, we ensure that whenever storage volume is discussed, the associated pressure is also clearly stated, for example, on page 16, lines 364–365, and page 18, line 400.<br><br>We also included a discussion on how changing the storage pressure influences volume requirements (see page 12, lines 281–283). In addition, a figure has been added to the Supplementary Information (Method S3) to illustrate the volumetric energy density of CH_4 and how it varies with storage pressure.<br><br>All major updates related to unit changes can be found in the following locations: page 9, Figure 1; page 13, line 327; page 14, line 350; page 15, Figure 2; pages 15-16, lines 355-363; page 17, Figure 3; and pages 17-18, lines 390-402.

	the unit for energy capacity, which is more generic. A section on space requirements with the m3-unit could be added then. Similar for m^3/kW in Fig 2 - using kWh/kW or simply hours, is a good, tangible unit for storage capacity needed. If the authors want to maintain the m3-unit, this reviewer suggests adding more discussion around these issues.	
3	While well explained in the Methods and/or SI, the main manuscript lacks a couple of definitions and details, which this reviewer thinks should be provided in the main manuscript to deliver a full and cohesive story.<br><br>Examples include the initial description of a rSOC and the terms "no-anode off-gas (AOG)" and "hot-AOG".	Thank you for the suggestion regarding the need for clearer process-related descriptions.<br><br>In this revision, we have moved some key content from the Supplementary Information into the main manuscript to provide more detail on the process design, assumptions, and relevant results. These additions can be found on pages 5-6, lines 88-168, with particular emphasis on the clarifications added on page 6, lines 127-129.
4	In the analysis of demand, did you account for odd years with e.g. very cold winters? Or are the annual profiles identical? If you don't account for rare weather circumstances, you may be underestimating your storage requirements.	Thank you for your insightful comment.<br><br>The considered (demand and supply) profiles are based on projections of a fully decarbonized energy system by 2050, which inherently carry a high degree of uncertainty, although they originate from a reliable source (see page 13, lines 317-318).<br><br>We did not include outlier years but instead relied on the provided hourly profiles. While total energy capacity is unlikely to be significantly impacted by rare weather events, we acknowledge that power capacity requirements could be. To address this, we introduced a security factor of 10% in the rSOC system scaling to slightly oversize the system and ensure sufficient power delivery under most conditions. This adjustment is also reflected in the cost estimation of the storage systems.<br><br>Please refer to the Methods section, page 26, lines 621-623.
5	It is stated that the rSOC has zero self-discharge, but in the profiles "hot standby" is used. Presumably	Thank you for your comment.<br><br>In hot standby mode, no electrochemical reaction occurs, though a small

	this would require discharging the battery slightly. Has this been accounted for?	amount of energy is required to maintain the operating temperature. When switching between modes, such as from SOFC to hot standby, or vice versa, the system does require some response time. Based on consultation with the stack manufacturer, this transition typically takes around 15 minutes. This delay can impact overall performance and has therefore been included in our results, as introduced on page 8, lines 195-198, and further detailed in the Methods section titled "rSOC critical phases – mode switching" (page 30).<br><br>We fully agree that the most robust approach would be to integrate dynamic (transient) rSOC performance directly into the system optimization framework. However, from our understanding, this would require extensive study and experimental validation and is thus considered beyond the scope of this work. We have acknowledged this as a direction for future research (see page 21, lines 512-513).
6	The authors label some of the countries as needing "short-term storage behaviour" rather than long-term, but the required energy storage to cover the full year is still 100s of hours. By any current definition, that is long duration.	Thank you for your comment.<br><br>We agree that 100 hours of storage falls within the medium to long-term storage category. Therefore, in this revision, we have updated the terminology used to classify storage durations to avoid confusion. The revised term "short-term-oriented" now refers to storage needs where long-duration storage solutions do not clearly outperform an oversized conventional battery system.<br><br>Additionally, we have adopted 100 hours (approximately 4 days) as the new threshold to differentiate between short-term-oriented (light color) and long-term-oriented (dark color) systems in our visualizations.<br><br>These changes have been applied to Figures 2 and 3, as well as the corresponding discussions in the Results section (see page 16, lines 366-370; pages 17-18, lines 392-398). These conclusions stem directly from the modeling assumptions of conventional battery systems, detailed in the Methods section (lines 690-697).
**Comments provided in the manuscript's .pdf file**		
1	This is a power capacity cost, not a storage capacity cost?	Thank you for your comment.<br><br>In the revised manuscript, we have used a levelized cost of storage that includes energy storage (in batteries or molecules) and conversion of stored energy into electricity.<br><br>As with your previous suggestions, we also have updated all cost-related

		units in this revision to enhance clarity and ensure broader relevance for readers across various sectors.<br><br>More details on the cost unit changes can be found in the abstract (page 2, lines 24-26), the main text (pages 17-19, lines 390-402), and Supplementary Information Table B9.
2	Please expand this explanation to a more accurate description of what an rSOC is, e.g. "An rSOC system can be designed to store excess renewable electricity by converting it to an energy carrier, such as hydrogen or other fuels, that can be easily stored, and can later provide […]"	Thank you for your suggestion.<br><br>In the revised manuscript, we have modified the discussion on rSOC system. Please see the updated version on page 3, lines 40-52.
3	Please add energy storage capacity of the mentioned system.	Thank you for your comment.<br><br>The plant mentioned in the study (i.e., 25 kW fuel cell/75 kW electrolyzer in the Netherlands) represents an important first step toward continuous rSOC operation, achieving more than 500 hours of runtime. While the project does not include a storage tank and only considers buffer tank, it still serves as a valuable milestone toward large-scale energy storage applications. Although the rSOC system in this case is not directly integrated with long-term storage, the demonstration highlights critical progress in system durability and operation.<br><br>This has been clarified in detail on page 3, lines 56-61.
4	Please rephrase, difficult to understand.	We have revised the sentence for improved clarity. Please refer to page 3, lines 53-54.
5	This is still a lot. How practical is this to deal with in a real system? What would the RTE drop to if a more manageable amount was used, e.g. <10? HEX are often custom components and generally costly. They also add complexity.	Thank you for your question regarding the number of heat exchangers. We appreciate this thoughtful point.<br><br>In the SOEC mode, optimal heat recovery can reduce the electricity consumption by the electric heater placed at the upstream of stack. Hence, we employed a heat exchanger network synthesis to explore optimal heat recovery, which can then enhance the round-trip efficiency of the rSOC system.<br><br>We consider efficiency to be a critical metric, as it reflects the fundamental performance limit of the technology itself, what we see as a final

		bottleneck. While we acknowledge that pushing for maximum efficiency may result in a higher number of heat exchangers, we have also conducted additional analysis showing that many of these heat exchangers can be shared between the SOFC and SOEC modes.<br><br>Nonetheless, our focus here is on identifying the theoretical efficiency potential. We agree that cost, control complexity, and system manufacturability are important considerations, particularly for large-scale deployment, but we view these as the next steps once the performance potential is clearly defined. That is why we studied optimal heat exchanger network design in this study: to emphasize the long-term promise of rSOC technology and to inform the broader energy systems community of its full potential.<br><br>We have clarified this point in the manuscript, please see page 7, lines 150–157.
6	Please define this. I'm guessing you are talking about no recirculation of the fuel outlet gas stream compared to recirculating the gas stream hot. Please confirm. Also, what temperature are you referring to when you say "hot"? 700-800 °C pumps are not generally available (only a few suppliers at most), to my knowledge.<br>Also, "anode" is ill-defined, especially when discussing an rSOC.	Thank you for the comment. This is related to your earlier comment (Comment 3 – Overall Comments section).<br><br>We have added a clarification in the manuscript regarding the differences among no, warm, and hot anode off-gas (AOG) recirculation options.<br><br>In our setup, "hot" AOG refers to the gas downstream of the SOFC stack, which can be at a very high temperature. Several commercial suppliers do support such configurations. One of the key goals of our analysis was to assess which AOG recirculation strategy delivers the best system performance. While hot AOG recirculation offers certain advantages, it introduces significant challenges, particularly regarding compressor design.<br><br>Our results show that compressor efficiency drops substantially at high temperatures. This inefficiency outweighs the benefits gained from hot recirculation, leading us to conclude that this option may not be viable from an efficiency perspective. However, we acknowledge that with the development of high-temperature compressors, hot AOG recirculation could still reduce pipeline complexity and offer practical integration advantages.<br><br>This explanation has been added to the revised manuscript. Please see page 6, lines 127–129.
7	What is the energy storage capacity?	Thank you for the comment.

		The energy storage capacity is a direct outcome of the optimization process (that includes electricity price and carbon intensity profiles) and is not treated as a fixed input parameter.<br><br>This has been clarified in the revised manuscript on page 8, line 174.
8	It says annual profiles here, in plural. Is that in fact what you used? If only using one average annual profile, you would not account for a particularly harsh winter occurring, let's say, every 5 years or so.	Thank you for your comment. In the revised manuscript, we have reformulated the sentence for clarity.<br><br>We use two types of hourly profiles in each framework: cost and carbon intensity for the current energy system, and production and demand for the future energy scenario. The K-medoids clustering method is applied to derive typical days. We acknowledge that this method may slightly underestimate the minimum power requirement; therefore, a 10% security factor has been added, as also mentioned in response to Comment 4 (see Overall Comments section).<br><br>Please see page 8, lines 187-189, and the Methods section, page 26, lines 610–612 and 620–623.
9	Can you put this in context? E.g. add here what the average yearly CO2 emission is per capita in Switzerland, or something like that.	Thank you for the interesting suggestion.<br><br>We have contextualized this number using per capita CO_2 emissions in Switzerland.<br><br>Please see page 8, lines 191–192 of the revised manuscript.
10	What is assumed for hot standby technically? Presumably the system will discharge slowly while in hot standby.	Thank you for the comment. This comment is related to Comment 5 in the Overall Comment section, where we provide a clear explanation.<br><br>In the revised manuscript, the corresponding changes can be found on page 8 (lines 195–198) and are further detailed in the Methods section titled "rSOC critical phases – mode switching" (page 29). Additionally, future work addressing this point is discussed on page 21, lines 512–513.
11	I think it would be more generally applicable to use the unit of kWh. You could store the energy in H2 or other chemicals, or in CH4 at a different pressure and/or temperature. This would change the volume significantly.	Thank you for the suggestion.<br><br>This suggestion aligns with your previous remark in the Overall Comment section, which we addressed by updating all relevant units to be based on kWh. In addition, we included a discussion on the impact of pressure in both the main text and the Supplementary Information to improve clarity and consistency.<br><br>The manuscript has been revised accordingly, with updates can be found

		on page 9 (Figures 1b and 1d), page 12 (lines 271–274), page 13 (line 327), pages 15–16 (lines 355–363 and Figure 2), and pages 17–18 (lines 390–402 and Figure 3). Further discussion and supporting data can be found in the Supplementary Information, Method S3.
12	Again, I would suggest the unit of kWh instead of m3. In fact, for Fig 2f, m3/kW (or kWh/kW) is simply the number of hours required (at full discharge), so that could also be used as the unit here.<br><br>I calculate a range here of 240-1845 h, with the extremes being Estonia and Switzerland, respectively. Those are very large numbers. Are you accounting for energy transmission between countries or are they considered isolated?<br><br>Regarding the colors in Fig 2f. I think labeling any of these countries as needing short-term or hybrid storage is misleading. The minimum required storage duration is 240 h, which is definitely long-term storage. However, I do agree that a hybrid approach with multiple storage solutions is probably beneficial.	Thank you for your insightful comment. We have updated all optimization results to be expressed in terms of kWh (capacity) instead of volume, as detailed in Comment 2 in the Overall Comment section.<br><br>Initially, Figure 2 presented storage capacity normalized by the average electricity demand, which cannot be directly interpreted as storage duration in the way you calculated. In this revision, we chose to normalize by maximum electricity supply instead, allowing for a direct comparison with the results shown in Figure 3.<br><br>Each country has its own input data and optimization results derived from the framework. However, we agree with your point that incorporating energy exchange between countries would be a valuable extension. Such integration is strongly influenced by country-specific energy policies across Europe and falls outside the current study's scope. Nevertheless, we acknowledge its relevance and have now included this point as future work on pages 21-22, lines 513–516.<br><br>Regarding the color labeling and the classification of country profiles, we have revised the color standard and updated the corresponding explanation, as addressed in Comment 6 of the Overall Comment section.<br><br>These changes have been reflected in Figures 2 and 3, as well as in the related discussion on page 16, lines 366–370, and pages 17–18, lines 392–398. Methodological details can be found in the Methods-Energy System Integration section, page 26-28.
13	These storage volumes are quite large, and I think it warrants some discussion. E.g. if you had an industrial plant or data center requiring 2 MW, that would call for a storage volume of 44m x 44m x 2.6m. That's ~65 40' shipping containers! One option around this would be to store the CH4 at 200-250 bar or as LNG, which would reduce the volume to a much more	Thank you for your comment. This is aligned with the comment you raised in the Overall Comments section.<br><br>To address this point, we have included a rough estimation of the number of containers required. By providing the storage volume, readers and industrial stakeholders can adapt the spatial layout based on their specific site conditions and constraints.<br><br>Regarding CH4 energy density, we have clarified this in the main text (Method section, Table 1, page 28), where a storage pressure of 75 bar is used as the baseline. As this pressure is just inside the supercritical region

	managable 1.5-5 m3. At 75 bar, you almost have a similar energy density as that of a high-end Li-ion battery.	of CO_2, we have ensured that whenever storage volume is discussed, the corresponding pressure is explicitly stated, for example, on page 16, lines 364–365, and page 18, lines 400–402.<br><br>We also discussed how variations in storage pressure affect volume requirements (see page 12, lines 281–283). Additionally, a figure has been added to the Supplementary Information (Method S3) to illustrate the volumetric energy density of CH_4 and how it changes with storage pressure.
14	Is gray color a normalized storage size of 2 or does it indicate that the countries have not been analyzed? If the former is the case, I suggest removing grey as a color option and only using red and blue.	Thank you for your comment.<br><br>In the initial Figures 3(a) and (b), we acknowledge some inconsistency with the colors for non-considered countries. In the revised manuscript, we have changed the color of non-considered countries to light yellow to avoid confusion.

Short Highlights to Reviewer 2:

Thank you very much for your thorough review and insightful suggestions. Your comments addressed all key aspects of the manuscript, from the introduction and methodology to the results presentation and the Supplementary Information and have been fully addressed in the revised version.

We especially want to express our appreciation for your focus on the process modelling section. We are grateful for the value and credit you placed on this part of the work. Further, we fully agree that process modelling is the foundation before moving to system-level optimization. Initially, we were concerned that this might not be commonly recognized by a broader audience, which led us to move much of the modelling content to the Supplementary Information. However, based on your comments, we have now found a better balance and ensured continuity and clarity in the main text.

All detailed responses are provided below.

Thank you again for your expert feedback and valuable support.

The following Table addresses 1st Revision comments from Reviewer 2.

No.	Comments	Corrections and Responses
1	Major purpose of the proposed system appears to be the application of ejectors and the hybrid tank.<br><br>However, several previous studies have already reported anode gas recirculation using ejectors.<br><br>Moreover, the performance improvement resulting from the hybrid tank configuration is not clearly highlighted in this work.<br><br>The authors should clarify the novel contributions of the ejector compared to prior literatures and explain the performance differences between systems with	Thank you for your insightful feedback and constructive comments.<br><br>Indeed, several studies and patents have reported the use of ejectors, particularly in traditional SOFC systems. However, our design introduces the use of two ejectors within the SOFC system, which we believe represents a notable innovation. A patent application covering this concept has been submitted and is currently under evaluation. We have now clarified this point more explicitly in the revised manuscript. Please refer to page 5, lines 91–95, and pages 5-6, lines 108–116.<br><br>Regarding the hybrid tank design, the concept has been patented, and it is now in the process of being adopted by an industrial partner. The novelty of this design is now clearly explained in the Methods section (see lines 533–542).<br><br>More broadly, we appreciate your suggestion to better highlight the system-level innovations. We agree that some key system-related details, including novel features, were previously located in the Supplementary Information. In this revision, we have relocated selected content to the main text in a more concise form, see for instance pages 5-6, lines 109–

	and without the hybrid tank.	116, and page 6, lines 135–141.
2	System modification seems critical in this study, but all relevant schematics and layouts are presented only in the supplementary figures rather than in the main text. Why?	Thank you for your question, and we appreciate the value you placed on the system modeling component of our study.<br><br>Initially, we assumed that readers might be more interested in the energy system optimization and its associated results, so most of the process modeling details and outcomes were placed in the Supplementary Information. However, we fully agree that the process modeling forms a critical foundation for our study. Even though energy system researchers may not require all technical details, it is still essential to present the key methods, assumptions, and outcomes clearly in the manuscript to ensure transparency and completeness.<br><br>Therefore, in the revised manuscript, we have re-integrated important process modeling content to strengthen the overall study and enhance its relevance and impact. Major updates can be found on pages 5-6, lines 108–116; page 6, lines 135–141; page 23, lines 533–542; as well as in Figure 4.
3	In Figure 1, on what basis were Solutions A-J selected?	Thank you for the question.<br><br>Solutions A and J represent the two extreme cases of the single-objective optimization: Solution A corresponds to the configuration with the lowest OPEX, while Solution J achieves the lowest CO_2 emissions. All other solutions, between A and J, represent trade-offs between these two objectives.<br><br>We have clarified this in the revised manuscript on page 11, lines 243–255.
4	The key advantage seems to be the reduction of two heat exchangers via ejector integration.<br><br>However, what are the potential trade-offs or technical issues that may arise from this simplification?	Thank you for the question.<br><br>We have listed several key advantages of the proposed rSOC system design (e.g., high round-trip efficiency, improved heat management, etc.). We have also acknowledged several practical challenges that could add complexity to the system, such as pressure control, customized component design, and operational stability.<br><br>We consider efficiency to be a critical metric, as it reflects the intrinsic performance limit of the technology, what we see as the final bottleneck. While we agree that optimizing for maximum efficiency might lead to lower TRL designs and introduce trade-offs in terms of cost, control complexity, and manufacturability, we view these as important follow-up

		steps once the system's performance potential is well established. That is why we chose to present the optimal efficiency design in this study, to highlight the long-term potential of rSOC and inform the energy systems community of its full capabilities.<br><br>We have revised the manuscript, to better our intent, limitations, and potential improvement areas. Please see page 7, lines 159–168.
5	Is the integration of ejectors and hybrid tanks feasible in practical systems? Are there any experimental precedents?	Thank you for your comment.<br><br>Several companies and research institutions have already explored the application of ejectors, which we have cited in the manuscript. For the hybrid tank design, we have also cited a patent from our research group, and it is now in the process of being adopted by an industrial partner in Switzerland. Please see page 23, lines 533-543 for details.<br><br>This raises an important question about the sequence between experimental validation and system-level design: should system innovation follow established experimental results, or can a novel system concept serve as a guiding vision for future experiments and manufacturing? From our perspective, the two are strongly interconnected, each informing and advancing the other. While extensive experimental validation is not yet available, some elements of the concept have been demonstrated previously. Our intention is to share this new system-level concept with the broader energy systems community, as we believe it offers promising improvements that may inspire future research directions and technical development.
6	The relationship between the proposed system design and the long-term and short-term energy storage strategies remains unclear.<br><br>The two seem to be largely independent-could the authors clarify this?	Thank you for the valuable feedback.<br><br>We agree that the connection between these two components was not clearly articulated in the initial manuscript. From our perspective, the development process forms a continuous loop: it begins with a proposed novel system design, which is then evaluated through system-level modeling. Within this model, some key components are already supported by experimental validation. Once the system model demonstrates optimal performance, we scale it up to the energy system level, where it can inform and guide storage strategy discussions to the broader energy community. If the community finds the concept promising, it can, in turn, motivate further targeted experimental validation. This feedback loop can continue iteratively, with each step reinforcing the others, ultimately leading to a robust and impactful design.

		In the revised manuscript, we clarified this connection to emphasize that each step, from concept to modeling to energy system integration and optimization, is essential and tightly linked to ensure a meaningful outcome.<br><br>These changes can be found on page 7, lines 159–168.
7	In Figure B1, are the six KPIs selected based on a standardized framework? How about CO_2 emissions? I think that six KPIs appear to emphasize the benefits of ejector integration, yet do not reflect its limitations-such as parasitic energy losses, control challenges during mode switching, or operational instability under non-steady-state conditions.	Thank you for your insightful comment.<br><br>Indeed, in the Supplementary Information, we presented six KPIs to assess how the proposed system design compares with a more conventional setup. Our focus was specifically on evaluating the performance improvements driven by our design choices, rather than listing all general KPIs typically associated with rSOC systems.<br><br>To clarify this scope, we have now added a short explanation before Table B3 in the SI (SI document: page 18).<br><br>That said, we fully agree that integrating rSOC systems into the broader energy landscape involves many more steps before reaching large-scale deployment. In particular, challenges such as rSOC response to dynamic electricity profiles and control strategies in transient operation require further investigation. These are complex topics that cannot be fully addressed in a single section, and we believe they deserve dedicated studies.<br><br>We have acknowledged this limitation and included these topics in the Future Work section (see pages 21–22, lines 512–519).
8	On page 13, line 272, Spain is reported to have a wind energy share of 0.8, yet it is classified as dark green in Figure 2f. What is the quantitative basis for the color classification?	Thank you for this valuable comment.<br><br>We fully agree that establishing the color classification on a clear quantitative basis is important. Initially, we relied on a combination of parameters, including the number of mode switches, normalized storage capacity, and general storage behavior.<br><br>However, to improve clarity and consistency, we have revised the terminology used for classifying storage durations. The term "short-term-oriented" now refers specifically to storage needs where long-duration storage solutions do not clearly offer a performance advantage over an oversized conventional battery system.<br><br>Furthermore, we have introduced a quantitative threshold of 100 hours (approximately four days) to distinguish between short-term-oriented

		(light color) and long-term-oriented (dark color) systems in all visualizations. This threshold provides a clearer basis for interpreting the results.<br><br>These updates have been implemented in Figures 2 and 3, along with the related discussions in the Results section (see page 16, lines 366–370; page 17–18, lines 390–398).
9	In Figure 2f, what is the correlation between the number of mode switches (SN)/ normalized storage volume (V) and the trend as the long-/short-term storage characteristics?	Thank you for your comment.<br><br>Before conducting our modeling work, we also expected that there might be a correlation between the number of mode switches and the suitability of short-term- or long-term-oriented storage. However, after analyzing the results, we found no clear or consistent relationship between mode switching frequency and storage volume or energy storage capacity, as it is a multi-parametric decision. In fact, systems with frequent mode switching not necessarily require smaller storage capacities. To ensure clarity and simplicity in our communication, we ultimately chose to classify the countries based solely on storage duration, as explained in your Comment 8.<br><br>Furthermore, in response to another reviewer's suggestion and to broaden the impact of our study, we updated all units to a standardized format. We believe this is an important improvement. By expressing storage in terms of energy capacity (kWh) rather than volume (m^3), we eliminate dependencies on external conditions such as pressure and temperature and present a more robust comparison that reflects the intrinsic characteristics of each storage technology. Accordingly, all results in the revised manuscript are reported using kWh as the primary unit.<br><br>All major updates related to unit changes can be found in the following locations: page 9, Figure 1; page 13, line 327; page 14, line 350; page 15, Figure 2; pages 15-16, lines 355-363; page 17, Figure 3; and pages 17-18, lines 390-402.
10	On page 13, line 281, the manuscript states that rSOC and batteries can be co-located with renewable sources for distributed storage but pumped-hydro storage is widely known to be site-constrained.	Thank you for your comment. We appreciate this thoughtful point.<br><br>In fact, we also hesitated initially about whether to include pumped hydro in the study or not. However, our intention was not to compare its storage capacity directly with other technologies. Instead, we aimed to highlight that, despite its well-known large storage volume requirements, pumped hydro should still be considered a valid option in countries with suitable natural conditions. We believe that if a country has the geographical potential to utilize this technology, it should not be ruled out based solely

	Is a direct comparison between rSOC and pumped hydro appropriate?	on volume considerations. For this reason, we included only five representative countries where such natural suitability exists.<br><br>In the revised manuscript, we have clarified this point more clearly to avoid confusion, please see page 19, lines 432–443.
11	The thermal energy input required to maintain 750°C operation in rSOC is not clearly accounted for in the cost analysis.<br><br>Would this omission significantly affect the viability or cost-competitiveness of the proposed system?	Thank you for your comment.<br><br>Indeed, we operated the SOEC in thermoneutral mode, which requires an external heat supply. To address this, we optimized a heat exchanger network to determine how much of the high-temperature heat demand could be met using available waste heat within the system. The remaining heat requirement, after waste heat recovery, is supplied by an electric heater. Conversely, heat is produced during SOFC operation.<br><br>This consideration is reflected in Tables A1 and B1 in the SI.
12	In Table B1 of the SI, why does the total HEX area increase in the proposed SOEC design?	Thank you for your observation.<br><br>We performed heat exchanger network synthesis, as explained on pages 25-26, lines 597–604 with supported cited reference. The objective functions aim to minimize both the number of heat exchangers and the utility usage. As a result, this may lead to a larger total heat exchanger area. This is because maximizing heat recovery requires smaller temperature differences between streams, which increases the required surface area.
13	The visual roughness in Figures 2c and 2e, as well as the comparative storage trends shown in Figures 2 and 3, would be more interpretable with the inclusion of quantitative metrics or statistical summaries.	Thank you for your comment.<br><br>Regarding these figures, we chose to refer to storage capacity rather than storage volume to ensure more interpretable results and to avoid potential bias due to volumetric energy density, as explained in our response to Comment 10.<br><br>We have also updated Tables B7 and B8 in the SI and added a new Table B9 to consolidate all relevant information related to the optimization results.<br><br>Finally, we increased the text size in the figures to improve visual clarity.
14	In Table B2, why does the temperature rise downstream of C"2?	Thanks for your careful checking.<br><br>In this revision, we have updated the typo, changing from C”2 to H”2.

15	Please specify the data sources for Figures 2 and 3 more clearly.	Thank you for your comment.<br><br>We now clearly cite the source of the data as Santecchia et al., “The critical role of electricity storage for a clean and renewable European economy”.<br><br>The citation has been added on page 13, lines 317–319.

Short Highlights to Reviewer 3:

Thank you very much for your thorough review and insightful suggestions. All of your comments have been fully addressed in the revised version.

We would especially like to thank you again for pointing out the incorrect figure reference, this was indeed a typographical error and has now been corrected. Additionally, we appreciate your suggestion to expand the study scope beyond Europe. As noted by the Editor, this is more appropriate to address in future work rather than in the current manuscript. We agree with this perspective, as the current study is already quite extensive. However, we truly value your suggestion and have already started gathering data with the aim of extending the analysis beyond Europe. This is an ongoing work.

We also hope that through this study, we can build more international connections and explore potential collaborations. One of the current challenges we face is data availability across regions, but we will continue working on this and aim to uncover further insights at a global scale.

All detailed responses are provided below.
Thank you again for your expert feedback and valuable support.

The following Table addresses 1st Revision comments from Reviewer 3.

1	This is curious: the main text only includes four figures, yet the authors reference "Figure 6". Where are Figure 4 and Figure 5?	We are grateful for your careful checking.<br><br>We have corrected this typo in the revised version.
2	As we know, the energy costs are influenced by many factors, such as differences between seasons, climates, and regional environments. Therefore, have the cost data provided in the paper considered these factors? Are the data sources reliable?	Thank you for your comment.<br><br>The cost data used are spot market prices at the national level for Switzerland in the year 2019, incorporating all relevant factors. Further, the provided profiles are reliable, as cited. However, we acknowledge that the analysis is based on a single year due to data availability.<br><br>We have stated the year of electricity (2019) that we considered, see page 26, lines 610–622.<br><br>In our study, the cost analysis focuses on comparing trends across countries rather than emphasizing absolute values. We believe this approach remains valid, as cost is inherently unstable and influenced by various factors such as national policies, government decisions, and manufacturing capabilities. Therefore, our discussion prioritizes the

		comparative trends rather than the precise monetary values.
3	The authors primarily discuss Europe's energy framework in the paper. What about other continents? Could the research results and conclusions be applied to them? It is recommended that the authors conduct an appropriate analysis of energy frameworks in other regions as well, the Asian region for example.	Thank you for the suggestion.<br><br>In the revision email, the Editor has encouraged us to focus more on Europe, which we have done in this study. Thus, we have included this suggestion into future work and limitation.<br><br>However, we agree that extending the analysis to other regions, such as Asia, would be highly valuable for future research. At present, we are limited by data availability and therefore focused on Europe, where consistent and reliable data is accessible. Nevertheless, we believe the key findings and overall trends remain relevant across regions. We hope that once this study is published, it will encourage broader collaboration and data sharing, contributing to a more comprehensive understanding of energy storage solutions globally.<br><br>Please see page 26, lines 516-520.
4	The text labels in some figures (e.g., Figure 6) are too small to be clearly visible. Please revise this issue.	Thank you for your comment.<br><br>We have increased the font size in most of the figures and improved their overall quality. All information, including text labels and results, should now be clearly visible to readers. Please see Figures 1, 2, and 3.

Short Highlights to Reviewer 4:

Thank you very much for your thorough review and insightful suggestions. Your comments addressed all key aspects of the manuscript, from the introduction and methodology to the results presentation and Supplementary Information and have been fully incorporated into the revised version.

We especially want to express our appreciation for your comprehensive and detailed feedback. It helped guide us through a careful revision of every section, prompting us to cross-check key details, add further references, and improve the overall clarity and transparency of the manuscript. We are truly grateful for your effort and contribution to strengthening this work.

All detailed responses are provided below.

Thank you again for your expert feedback and valuable support.

The following Table addresses 1st Revision comments from Reviewer 4.

1	The proposed process modelling of the rSOC system does not appear sufficiently accurate nor the simplifications are acknowledged.<br><br>The neglect of CAPEX in favour of focusing on OPEX is also a critical aspect when extending suitability assessment and comparison with other technologies.	Thank you for your comment regarding the modeling accuracy. We also appreciate your suggestion on including CAPEX.<br><br>For rSOC system, all key components, except ejector and hybrid tank, have been validated using experimental data, and their reliability has been demonstrated in a previous study, which we have cited (see page 25, lines 593–595).<br><br>While we initially focused on cost trend comparisons due to the inherent instability of monetary values (influenced by factors such as national policies, government decisions, and manufacturing capabilities), we fully agree that including CAPEX adds valuable perspective and can enhance the impact of the study. Therefore, we have now incorporated CAPEX considerations into the Discussion section, with a detailed explanation of the methodology used (see page 20, lines 464–472).
2	Overall, the authors try to combine process simulation (of which only efficiencies are mentioned in the main text) and system optimization (more extensively explored) and leave out many details of the core technology.	Thank you for your comment, and we appreciate the value you placed on the system modeling component of our work.<br><br>Initially, we assumed that readers might be more interested in the energy system optimization and its associated results, so most of the process modeling outcomes were placed in the Supplementary Information. However, we fully agree that the process modeling forms a critical foundation for the study. Even though energy systems researchers may not require all technical details on process modelling, it is still essential to

		present the key methods, assumptions, and outcomes clearly in the main manuscript to ensure transparency and completeness.<br><br>Therefore, in the revised manuscript, we have re-integrated important process modeling content to strengthen the overall study and enhance its relevance and impact. Major updates can be found on pages 5-6, lines 108–116; page 6, lines 135–141; page 23, lines 533–542; as well as in Figure 4.
3	At the same time, the authors try to fit an excessive amount of information in a short document, with many descriptive areas that do not sufficiently convey strong messages.	Thank you for your insightful feedback and constructive comment.<br><br>We have conducted extensive work, covering both process modeling and energy system integration, as we believe these two aspects are strongly interlinked.<br><br>In the system modeling part, our key novelty lies in the application of ejectors and the hybrid tank. While several studies and patents have explored the use of ejectors, particularly in SOFC systems, our design uniquely integrates two ejectors within the SOFC configuration. We believe this represents a meaningful innovation, which is currently under evaluation in a submitted patent application. This point has now been more explicitly clarified in the main manuscript (see page 5, lines 91–95, and page 6, lines 118–123). Regarding the hybrid tank design, it has been patented, and it is now in the process of being adopted by an industrial partner. The novelty of this concept is now clearly described in the Methods section (see lines 533–542).<br><br>From the energy system integration perspective, a key contribution of our study is providing country-specific storage capacity estimates for each technology considered. We believe these values can serve as useful references for both industrial practitioners and academic researchers.<br><br>More broadly, we appreciate your suggestion to better highlight the innovations. We agree that some key process-related details, including novel features, were previously presented only in the Supplementary Information. In this revision, selected content has been relocated to the main text in a more concise form, for example, see pages 5-6, lines 108–116, and page 6, lines 135–141.
**Abstract**		
4	The authors mention (line 22) a sizing of "0.26 to 4.47 m3/kW_avg,demand", which is	Thank you for the comment.<br><br>In line with similar feedback from other reviewers, we acknowledge that the units initially used were not straightforward and could cause

	unclear: m3 of what? what is "avg,demand"?	confusion. Therefore, during this revision, we made a concerted effort to convert all units to a standardized format. Specifically, we now use storage capacity (kWh) rather than storage volume (m^3), as this ensures consistency throughout the manuscript and avoids ambiguity due to dependencies on temperature, pressure, or other external factors. This approach allows the results to more accurately reflect the intrinsic characteristics of each storage technology.<br><br>All major updates related to unit changes can be found in the following locations: page 9, Figure 1; page 13, line 327; page 14, line 350; page 15, Figure 2; pages 15-16, lines 355-363; page 17, Figure 3; and pages 17-18, lines 390-402.
5	The sentence (line 23) "The rSOC technology offers a storage cost of 25-35 k€/kWmax, supply in certain regions, whereas the battery system shows storage costs can be lower than 25 k€/kWmax,supply in others". In general, storage cannot be defined merely in terms of kW, since the energy capacity (kWh) is also relevant. In batteries, energy and power capacities are not independent. In rSOC, they are.<br><br>The value for rSOC investment cost of 25-35 k€/kW seems immensely high, given that SOFC are listed in the range of 2-5 k€/kWmax.	Thank you for your valuable comment regarding the initially chosen unit.<br><br>Initially, our intention was to consider the perspectives of all stakeholders, including stack manufacturers and industrial partners. However, during this revision, we recognized that adopting a standard energy storage unit better serves the broader audience. Therefore, we have updated all economic-related units to €/kWh, including the Levelized Cost of Storage (LCOS). This metric captures round-trip efficiency, maximum energy capacity, number of storage cycles, and power requirements in a single economic value. We believe this change, inspired by your comment, will enhance the relevance and clarity of the manuscript for readers focused on energy storage systems.<br><br>While all numerical values have been revised accordingly, the core message and methodology remain consistent with the original submission. These updates improve the communication of our findings without affecting their substance.<br><br>More information on the main changes related to the economic units can be found in the abstract (page 2, lines 24-26), the main text (pages 17-18, lines 390-402), and Supplementary Information Table B9. Other changes to units not directly related to the economic analysis appear on page 9 (Figures 1b and 1d), page 13 (line 327), page 14 (line 350), and pages 15-16 (lines 355-363).<br><br>Regarding the second aspect concerning the high investment cost of rSOC, we acknowledge that the estimated system price—around 20 k€/kW—is indeed high. This value is derived from the data presented in Table 1, assuming an initial CAPEX of 10 k€/kW and a system lifetime of 10 years. While this may appear conservative for future projections, we chose to adopt a cautious approach, supported by cited references. See page 28,

		line 657.<br><br>Nevertheless, our cost analysis focuses primarily on cross-country comparisons rather than absolute cost values. We believe this approach remains valid, as cost values are inherently unstable and subject to variation due to national policies, government decisions, and manufacturing capabilities. Therefore, our discussion emphasizes comparative trends rather than exact monetary values.
**Introduction**		
6	The first sentence of the Introduction may be misleading: climate change is mostly driven by CO2 emissions, which are not technically a "pollutant" but a "climate altering gas".	Thank you for your observation.<br><br>In the revised manuscript, we have revised the sentence to avoid any potential misinterpretation. Please see the updated text on page 3, lines 33–34.
7	Lines 38-41, it may be beneficial to define short- and long-term.	Thank you for your suggestion.<br><br>In this revision, we have now clarified the definitions of short- and long-term storage and provided supporting references. Please see the updated explanation on page 3, lines 43–45.
8	Line 47, "flat geographical areas" are mentioned and their definition is not clear.	Based on your suggestion, we have revised the sentence for improved clarity. Please refer to page 3, lines 53-54.
9	Line 49, other projects have also addressed and tested rSOC technology, please extend your review.	Thank you for your comment.<br><br>The plant mentioned in the study (i.e., 25 kW fuel cell/75 kW electrolyzer in the Netherlands) represents an important first step toward continuous rSOC operation, achieving more than 500 hours of runtime. While the project does not include a storage tank and only considers buffer tank, it still serves as a valuable milestone toward large-scale energy storage applications. Although the rSOC system in this case is not directly integrated with long-term storage, the demonstration highlights critical progress in system durability and operation.<br><br>This has been clarified in detail on page 3, lines 56-61.
10	Line 58, the presence of a methane-CO2 streams is mentioned for the first time, which was not introduced (the reader	Thank you for your comment.<br><br>In the revised manuscript, we have revised the sentence for improved clarity. Please refer to page 4, lines 68-70.

	may think of co-electrolysis or methanation, with many doubts and no discussion).	
	**Results**	
11	An image of the system scheme under analysis would be appreciated at the beginning of the Results section. Or at least referring to the image in the Methods section.	Thank you for your suggestion.<br><br>We agree that including a rSOC system layout for both operating modes can greatly enhance reader understanding. Therefore, in the revised manuscript, we have added a new figure (previously in the Supplementary Information) to the Methods section, see Figure 4 on page 24.<br><br>This figure is also referenced in the Results section to ensure a clear link between system description and performance discussion.
12	The need for and the integration of a Rankine Cycle is not clear.	Thank you for your comment regarding the integration of Rankine cycle (RC). We appreciate this thoughtful point.<br><br>In this study, we employed an RC that can valorize the waste heat available from the rSOC system. This integration enhances the round-trip efficiency of the rSOC system. We consider efficiency to be a critical metric, as it reflects the fundamental performance limit of the technology itself, what we see as a crucial bottleneck. While we acknowledge that pushing for maximum efficiency may result in a complex system design.<br><br>We agree that cost, control complexity, and system manufacturability are important considerations, particularly for large-scale deployment, but we view these as the next steps once the performance potential is clearly defined. That is why we chose to present the optimal efficiency case (with RC integration) in this study: to emphasize the long-term promise of rSOC and to inform the broader energy systems community of its full potential.<br><br>We have clarified this point in the revised manuscript, please see page 5, lines 95-99; pages 6-7, lines 145–150.
13	The definition of "conversion efficiency" at line 77-78 is unclear (e.g., is that electricity-to-LHV?).	Thank you for pointing this out.<br><br>In the case of SOEC, the conversion efficiency is defined as the ratio of the chemical energy content of the produced methane to the total electrical power input to the system.<br><br>This definition has been included in the Supplementary Information. Please see page 4, Method S1: SOEC system design.

14	In Figures A1 and A2 in SI, electricity seems to be transferred from the grid to the rSOC stack. How is power electronics considered? This deserves some discussion.	Thank you for your valuable suggestion.<br><br>We agree that this is a critical consideration when integrating rSOC systems with the grid, as several studies highlight the need for precise control strategies to ensure stable and efficient operation. We find this to be a particularly interesting point and have included it as a potential direction for future work. This has now been added to the Supplementary Information, please see page 8.
15	Line 81, it is not clear what the "cold utility demand" refers to.	Thank you for your comment.<br><br>We used cold water as the cooling source (or cold utility). In the revised manuscript, we have clarified this point for improved clarity, please see page 5, line 107.
16	Information on the electrochemical assumptions is essential (thermoneutral voltage? ASR taken from where? 0D or 1D modelling?)	Thank you for your valuable comment.<br><br>The ASR value has been obtained from experiments based on IV curve measurements. This study has developed a 0D model of rSOC system that has been validated using experimental data, as cited. As mentioned in our response to Comment 1, model validation is not the primary focus of this study. However, we have briefly described this aspect in the Methods section, along with a citation to the relevant reference, see page 25, lines 593–595.
17	The rationale behind the (reasonable) choice of the ratio 1:3 for SOFC and SOEC nominal power is not expressed.	Thank you for your comment.<br><br>In SOEC mode, the same stack operates at approximately twice the voltage and 1.5 times the current compared to SOFC mode. As a result, the SOFC power output is roughly one-third of the SOEC power input.<br><br>This explanation has been included in the revised manuscript, please see page 6, lines 123–125.
18	The SI unit for "metric tonne" is "t".	Thank you for your careful checking.<br><br>We have updated it throughout the manuscript.
19	Lines 127-130: it is not clear whether the thresholds at 130 and 200 gCO2/MWhe are input values or results.	Thank you for your comment.<br><br>This represents one of the optimization outcomes of our current energy analysis of Switzerland. We have now clarified this more clearly in the revised manuscript, please see pages 8–9, lines 199–202.

20	Fonts in Figure 1 are too small.	Thank you for your observation.<br><br>We have increased the font sizes in most of the figures, including Figure 1, to improve their readability.
21	Line 140, incorporating O2 sales is interesting, but the use of a pure O2 stream or the separation of O2 from the enriched air are not straightfoward and need discussion. The sentence in line 147 is weak.	Thank you for your concern.<br><br>We acknowledge that collecting pure O_2 from SOEC is not a common practice. Although experimental testing has shown that producing O_2 as a byproduct does not significantly affect stack performance. We believe it is not widely applied primarily due to safety concerns. However, this barrier may be overcome if the scientific community recognizes the potential value of O_2 recovery. In the revised manuscript, we have provided a clearer explanation of this point.<br><br>Please see page 10, lines 225–232, and the Methods section on page 29, lines 673–681.
22	Line 143 talks about "maximising economic returns", but the results are presented as "min CO2" optimization.	Thank you for your comment.<br><br>Solutions A and J represent the two extreme cases of the single-objective optimization: Solution A corresponds to the configuration with the lowest OPEX, while Solution J achieves the lowest CO_2 emissions. All other solutions, between A and J, represent trade-offs between these two objectives.<br><br>In the revised manuscript, we have clarified this on page 8, lines 200-201; pages 10, lines 213-214, lines 237-240.
23	Line 162 mentions "CO2 increase". In general, the benchmark of the overall analysis is not clear.	Thank you for pointing this out.<br><br>The benchmark assumes no further use of electricity, which itself carries associated CO_2 emissions and a selling price. We have revised the wording to make this clearer.<br><br>Please see page 10, lines 237–240.
24	Lines 160-174 are strictly descriptive; there is no clear message.	Thank you for your comment.<br><br>In the revised manuscript, we have included a summary of key trade-offs. These results highlight the importance of identifying balanced operating strategies, as pursuing extreme outcomes, whether economic or environmental, may lead to significant compromises in the other objective. Such trade-offs require careful evaluation by the decision

		maker. Please see page 11, lines 255-259.
25	Lines 227-228: what is an "average electricity demand of 1 kW"? Is that the peak or the constant power output? If average, what is the actual profile and/or the max deviation?	Thank you for your comment.<br><br>The reference to an average electricity demand of 1 kW was originally introduced to facilitate the discussion around the implemented storage system and to provide scale-independent metrics. By using this reference point, we were able to size the storage systems based on a tangible average value applicable across different industrial contexts. The resulting figures could then be scaled-up or -down depending on the targeted level of centralization.<br><br>However, we acknowledge that this normalization may cause confusion, especially when discussing storage systems. As also noted in our response to Comment 5, we believe that converting all units to a standardized format is a crucial requirement. Using storage capacity rather than storage volume ensures consistency and eliminates potential misunderstandings related to external parameters such as temperature and pressure. This approach allows the results to reflect the intrinsic characteristics of the storage technologies.<br><br>Accordingly, we have updated all results in the revised manuscript to use kWh as the main unit. Major changes can be found in the following locations: page 9, Figure 1; page 13, line 327; page 14, line 350; page 15, Figure 2; pages 15-16, lines 355-363; page 17, Figure 3; and pages 17-18, lines 390-402.
26	Lines 232-234: is the ratio computed on solar+wind or including all RES (including geothermal and hydro)? What about biomass? referred to which year?	Thank you for your comment.<br><br>This computation is based on all renewable electricity sources, including solar, wind, and hydro. Biomass is not directly considered for electricity production, as it is assumed to play a critical role in other sectors in the future. These profiles and assumptions are based on the cited reference, see page 13, line 318.<br><br>The renewable generation profiles are computed for most EU countries within the context of a decarbonized energy framework for 2050.
27	The "hybrid tank for fuel and CO_2 storage" is never really presented. How does it work? How does it separate the fluids?	Thank you for your comment.<br><br>For the hybrid tank design, we have cited a patent from our research group. The concept is in the process of being adopted by an industrial partner in Switzerland. In the revised manuscript, we have included more details on the working of the hybrid tank. Please see page 23, lines 533-542 for

		details.<br><br>This raises an important question about the sequence between experimental validation and system-level design: should system innovation follow established experimental results, or can a novel system concept serve as a guiding vision for future experiments and manufacturing? From our perspective, the two are strongly interconnected, each informing and advancing the other. While extensive experimental validation is not yet available, some elements of the concept have been demonstrated previously. Our intention is to share this new system-level concept with the broader energy systems community, as we believe it offers promising improvements that may inspire future research directions and technical development.
**Methods**		
28	Line 428, "thermoneutral" is common in the SOEC field, rather than "thermal-neutral".	Thank you for pointing this out.<br><br>We have updated it. Please see page 24, line 553.
29	Line 429, the presence of pure O2 vs sweep air vs sweep O2 is not discussed and this is a shortcoming in supporting the resulting values.	Thank you for your comment.<br><br>This comment is similar to your Comment 21, where we have discussed various aspects of using sweep O2 for SOEC.
30	Line 431-433 (same present also in SI), the sentence "This system utilizes an ejector to reduce CO2 pressure instead of the traditional turbine, with high-pressure CO2 from the hybrid tank acting as the motive gas and atmospheric-pressure hydrogen as the flare gas." is not clear: an ejector is typically used to increase pressure, not reduce.<br><br>CAPEX of rSOC at 10 k€/kW seems excessively high, especially for future scenarios.	Thank you for pointing this out.<br><br>While the integration of ejectors into rSOC systems has been explored in several studies and patents, our design introduces the use of two ejectors within the SOFC system, which we believe represents a notable innovation. An ejector increases the pressure of a flare gas with the help of a motive gas, which loses the pressure. To clarify the role and function of the ejectors, we have included a more detailed explanation in the revised manuscript. Please see page 24, lines 555–559.<br><br>Regarding the second aspect concerning the high investment cost of rSOC, we acknowledge that the estimated system cost, around 20 k€/kW, is relatively high. This value is based on the data presented in Table 1, assuming an initial CAPEX of 10 k€/kW and a system lifetime of 10 years. While conservative for long-term projections, this assumption is supported by cited references and reflects our intention to adopt a cautious approach. Please see page 28, line 657.
31	The use of electricity for the entire preheating (incl. evaporation) of	Thank you for your observation.

	water before the SOEC stack makes it hard to believe the high conversion efficiency obtained, without thermal recovery from cathode outlet.	Please note that the electric heater only supplies a small amount of high-temperature heat for SOEC operation. In our rSOC system design, we have indeed included a heat exchanger network to optimize overall heat recovery. This is described in the Methods section (page 25, lines 597–598).
32	The valorization of waste heat from the rSOC to drive a Rankine cycle is hard to believe, since available temperature of the streams after thermal integration for the rSOC itself is below 300°C. Needs more info and support.	Thanks for the comment.<br><br>The rSOC system has high temperature waste heat to drive a Rankine cycle. To better illustrate the thermal integration potential of the rSOC system with Rankine cycle, we have included two integrated composite curves in the Supplementary Information, see Figure B2.
33	What is sigma in Eq. 1 (line 121)?	The sigma value (Table 1) represents the storage efficiency, specifically, how much stored energy is effectively transferred to the next hour. It is inversely related to the self-discharge rate on an hourly basis. We have now clarified this relationship with an additional explanatory line in the revised manuscript. Please see page 28, lines 647-649.
34	Line 533, 10% unconverted fuel means 90% utilization factor, which is extremely high. How is that possible?	Thank you for your comment.<br><br>The fuel utilization factor value is taken from specifications provided by the stack manufacturer, SolydEra. Please see pages 28-29, line 667-668.
35	Overall, it seems that the rSOC modelling is very simple / approximate. If that cannot be improved, make sure to highlight the assumptions and the limitations.	Thank you for your comment regarding the modeling accuracy.<br><br>The simulation models for all key components, except for the ejector and hybrid storage tank, have been validated using experimental data, and their reliability has been demonstrated in previous studies, which we have cited (page 25, lines 593–595).
		**Supplementary Information**
36	The submitted support material is almost like a second article. The section "novelties" is unusual there, since what discussed in the main document should be sufficient. This document should provide additional info, such as technological details, complete input and output data, not elements that are essential to understand the	Thank you for your valuable feedback on the content of Supplementary Information.<br><br>We divided the Supplementary Information into two sections, namely "Methodology" and "Results". As Nature Communication journal has limit on the number of words, we summarized more supportive literature (Method S0-1) and included details on key aspects of our study (Method S0-2) in the Supplementary Information. We thought this information could be valuable to interested readers. However, we fully agree with the

	main text. As observed above, there are also repetitions from the Methods section, which is undesirable.	Reviewer's point of view. Hence, in the revised Supplementary Information, we have modified and rearranged them in Method S0: Supportive Literature and Key Aspects. Further, we have placed a note at the beginning of Method S0, to avoid any potential misinterpretation.<br><br>During the revision, we made many more changes to the Supplementary Information, primarily by removing duplicated content related to system modeling and streamlining it to retain only the most essential and relevant information. Key parts previously in the Supplementary Information have been moved into the main manuscript to improve clarity and accessibility.<br><br>In addition, we have moved one figure from Supplementary Information to the Methods section of the revised manuscript. We believe these changes enhance the overall impact and readability for the reader.
37	Figure A4 in SI, it is curious that clustering leads to typical days with either low demand and high generation or high demand and low generation and no comparable demand and generation case. Please discuss.	Thank you for your observation.<br><br>Figure A4 (now Figure A5) in the Supplementary Information presents the typical day clustering results for Switzerland under both the current (price and CO_2 intensity) and future (production and demand) energy frameworks. The emergence of significantly different (in terms of demand and generation) typical days is expected. By 2050, Switzerland is anticipated to rely heavily on solar energy, resulting in electricity generation that is highly time-dependent, both daily and seasonally. Simultaneously, a substantial portion of future demand will be driven by heating needs, leading to higher electricity consumption during winter.<br><br>As a result, the clustering identifies three main categories: (a) Winter days with low PV generation and high demand; (b) Mid-season days with moderate PV generation and relatively high demand; (c) Summer days with high PV generation (partially curtailed) and lower demand.<br><br>We added this explanation in the SI under Figure A5.
38	Clustering method (k-means, k-medoids, hierarchical, k-milp, ...) is not described. List of time-varying quantities included in clustering is not presented.	Thank you for your comment.<br><br>The clustering method (k-Medoids) is described in the Methods section on page 26, lines 620–623. Further, more details are provided in the Supplementary Information below Figure A5.

Optimizing Reversible Solid Oxide Cell Technology for Long-Term Energy Storage in Europe's Decarbonized Frameworks

Xinyi Wei, Arthur Waeber, Shivom Sharma, Jan Van herle, Francois Marechal
Corresponding authors: xinyi.wei@epfl.ch; jan.vanherle@epfl.ch; francois.marechal@epfl.ch

Dear Editor and Reviewers,

We would like to sincerely thank you for taking the time to review our first revised manuscript and for providing additional minor suggestions from different perspectives. Your feedback has greatly improved the quality of our work and increased its clarity for the readers of Nature Communications.

In response to the reviewers' feedback, we have carefully revised the manuscript and addressed each comment appropriately. In this revision, we have added a sensitivity analysis for the cost assessment and further improved the clarity of text and figures to support better reader understanding.

Once again, we are grateful for the opportunity to refine our work, and we hope that the revised version meets your expectations. Thank you for your thoughtful review and consideration.

Sincerely Yours,

Xinyi Wei, Arthur Waeber, Shivom Sharma, Jan Van Herle, Francois Marechal

Short Highlights to Reviewer 1:

Thank you very much for your thorough review and insightful suggestions. We greatly appreciate your explanations regarding the cost aspects, as well as your careful examination of the manuscript not only from a reviewer's perspective but also from a reader's viewpoint. Your comments helped us identify potential sources of misunderstanding and improve our manuscript.

All detailed responses are provided below. Thank you again for your expert feedback and valuable support.

The following Table addresses the 2nd Revision comments from Reviewer 1.

No.	Comments	Corrections and Responses
1	While the cost may be true today for a relatively small demonstration system, like the 50/150 kW system used as an example, it will be far lower in the future. I am familiar with a group who have performed a very detailed (unpublished) economic study of this topic, taking economy of scale and a reasonable learning rate into account, and the estimated Euro/kW and Euro/kWh is far lower.<br>I understand that the authors do not wish to focus on absolute cost values as this is dependent on many parameters, while the efficiency is more intrinsic to the technology. However, since the absolute cost estimates are mentioned in the abstract when discussing Europe's 2050 energy landscape, I would suggest to try to consider economy of scale of all components and a learning rate for the SOC technology, to extend the cost range lower, if that happens to be the finding. This is very important. With some luck, policy	Thank you for this insightful and important comment.<br><br>We fully agree that the absolute cost values of rSOC systems are expected to decrease substantially in the future due to economies of scale, technological learning etc, particularly as the technology transitions from demonstration scale to commercial maturity.<br><br>Although our intention is not to predict precise future cost values, in this second revision, we have extended the cost analysis to explicitly explore the impact of potential rSOC cost reductions. Specifically, we performed a sensitivity analysis on rSOC capital expenditure, varying the assumed cost over a wide range from 1,000 to 10,000 €/kW. This range is intended to encompass both current demonstration-scale costs and plausible future cost levels that could be achieved.<br><br>The results of this analysis are presented in Figure 3(e) and discussed in the revised manuscript. They show that reductions in rSOC CAPEX can lead to substantial decreases in the LCOS across all considered countries, with differing magnitudes depending on national storage characteristics. This approach allows us to capture the potential impact of future cost reductions without relying on a specific learning rate or deployment trajectory, which would introduce additional assumptions and uncertainties. We believe this sensitivity-based framework provides a transparent and robust way to address future cost reductions, while remaining consistent with the system-level focus of the study.<br><br>Relevant changes and additions can be found in the revised manuscript on Page 19-20, lines 446-478, in Figure 3(e) and in Table B10 in the Supplementary Information.

	makers, investors and technology developers may base their decisions, in part, on publications such as this one, and the projected costs may influence funding of rSOC systems as opposed to other energy storage systems.	
2	On page 25-26, line 598-599, it is mentioned that heat can be generated in both SOEC and SOFC mode, and while this is true, stacks in SOEC mode are almost always operated at the thermoneutral potential (as also recommended by the chosen stack vendor in this study). So stacks in SOEC mode would not be generating heat. However, if the authors are thinking about the entire system, then it could be true that heat can be generated in charge mode (SOEC), since the methane synthesis is exothermic. If so, this should be rephrased as such.<br><br>Also, on page 7, line 150-151, the authors write that SOEC operation requires external heat to maintain thermoneutrality. This seems like a contradiction to the section above. Assuming you operate at thermoneutral voltage, there would be no need for external heat for the SOEC alone. This is possibly just a question of rephrasing these two statements to avoid confusion or misunderstandings.	Thank you for your careful reading and detailed suggestions. You are correct that our previous description could lead to misunderstandings.<br><br>When the SOEC operates under thermoneutral conditions, the stack itself does not generate additional heat. From a system-level perspective, although the SOEC outlet streams can be used to preheat the inlet streams, temperatures of input streams cannot be fully raised to the required level because of the minimum temperature-approach constraint. As a result, a small amount of external high-temperature heat is still required to reach the required temperatures of input streams. Nevertheless, when considering the entire SOEC system, including the downstream exothermic methanation reaction, there remains a net surplus of medium- and low-temperature waste heat that can be utilized elsewhere in the process.<br><br>We have revised the manuscript accordingly to clarify this point. Please see page 7, lines 150-154, and page 27, lines 646-650.

Short Highlights to Reviewer 2:

Thank you very much for your thorough review and insightful suggestions. In particular, we value your remarks on figures interpretation and on improving clarity from a reader's perspective, which helped us refine and clarify multiple parts of the manuscript to strengthen its overall readability and impact.

Detailed responses to each comment are provided below. Thank you again for your expert feedback and valuable support.

The following Table addresses 2nd Revision comments from Reviewer 2.

No.	Comments	Corrections and Responses
1	Figures 4 and 5 are still difficult to interpret. The image quality should be further improved and presented in a more visually accessible manner.	Thank you for your insightful feedback.<br><br>In this revision, we have adjusted the layout and increased the font size to improve visual accessibility.<br><br>Please see Figure 4 (page 25) and Figure 5 (page 29).
2	It appears that Figures 2c and 2e were derived from Figures 2b and 2d, respectively, but the method of derivation is not clearly explained. Additional clarification is required.	Thank you for your suggestion.<br><br>Figures 2(b) and (d) represent renewable energy production and demand profiles for Switzerland and Estonia, respectively, on typical days. We apply a statistical clustering approach on original annual time series data to extract a smaller number of representative/typical days. Please see page 14, lines 341-348.<br><br>Figures 2(c) and 2(e) are linked to the production–demand patterns shown in Figures 2(b) and 2(d) via activation of SOFC or SOEC mode. In the revised manuscript, we have clarified this relationship and provided an example to guide the reader on how to interpret these figures.<br><br>Please see page 14, lines 355–360.
3	In Figure 2b, the energy production graph shows a pronounced central peak across most of the 15 regions. Why does this occur, and why is a similar trend not observed in Figure 2d?	Thank you for your comment.<br><br>Figures 2(b) and 2(d) do not represent different regions or countries; instead, each of them represents the set of typical days generated for a single country (Figure 2b for Switzerland, Figure 2d for Estonia).<br><br>These typical-day profiles differ from the original annual time series

		obtained from the reference data. The full-year profile, with hourly resolution, is too large to be directly used in the optimization model. Therefore, we apply a statistical clustering approach to extract a smaller number of representative days. These typical days are then grouped (not sequenced chronologically) to approximate the characteristics of the full-year profile.<br><br>Importantly, neither the number nor the shape of the typical days is chosen manually; both are determined by the clustering algorithm. As a result, each country yields a different set of typical days. For example, Switzerland requires 15 typical days (Figure 2b), whereas Estonia requires 21 typical days (Figure 2d) to adequately represent their respective annual profiles. This procedure is explained in detail in the Methods section (page 28, lines 664-666) and the Supplementary Information (Figures A5 and A6 of the SI).<br><br>However, we agree that additional clarification in the main Results section improves readability. Therefore, we have added a short explanation on page 14, lines 341-348.
4	Since Figures 2 and 3 represent the major contribution of this manuscript, it would strengthen the novelty of the work if the authors could clearly distinguish which datasets were obtained from references and which were generated/simulated through their own analysis.	Thank you for your comment.<br><br>Figures 2 and 3 represent the generated results/simulated datasets. Figures 2(b) and 2(d) may have created some confusion as those represent renewable energy production and demand profiles for Switzerland and Estonia, respectively, on typical days. Please note that these profiles were obtained by applying a statistical clustering approach on original annual time series data from reference. In the revised manuscript, it has been clearly stated on page 14, lines 341-348.<br><br>No other sub-figures (in Figures 2 and 3) create any confusion.

Optimizing Reversible Solid Oxide Cell Technology for Long-Term Energy Storage in Europe's Decarbonized Frameworks

Xinyi Wei, Arthur Waeber, Shivom Sharma, Jan Van herle, Francois Marechal
Corresponding authors: xinyi.wei@epfl.ch; jan.vanherle@epfl.ch; francois.marechal@epfl.ch

Dear Editor and Reviewers,

We sincerely thank you for taking the time to review our manuscript.

In this revision, we have carefully addressed all the comments provided by the reviewers and have clearly indicated the corresponding changes with page and line numbers in the revised manuscript. We hope that this revision has strengthened the quality of our study.

Sincerely yours,

Xinyi Wei, Arthur Waeber, Shivom Sharma, Jan Van Herle, Francois Marechal

Short Highlights to Reviewer 1:

Thank you very much for your thorough review and insightful suggestions.

We have fixed the typo, and the detailed responses are provided below. Thank you again for your expert feedback and valuable support.

The following Table addresses the 3rd Revision comments from Reviewer 1.

No.	Comments	Corrections and Responses
1	Figure 3e, I believe the symbols for 1 k€/kW and 10 k€/kW should be switched, so that a lower rSOC CAPEX leads to a lower overall LCOS.	Thank you for your keen observation.<br><br>In the revised manuscript, we have corrected it.

Short Highlights to Reviewer 5:

Thank you very much for your thorough review and insightful suggestions. We are grateful that you have carefully reviewed the previous reviewer's comments and provided additional feedback and suggestions to help strengthen our study.

We particularly appreciate your comments regarding the description of the process design advancements in the main manuscript, as well as your questions on the clarification of the reference cases, along with other relevant points.

In this revision, we have carefully addressed all of your comments and indicated the corresponding changes, including page and line numbers, in the response document. Moreover, to facilitate your review, we have highlighted the previous reviewer comments in black and together with your comments in blue.

Thank you again for your valuable suggestions and for your careful review.

The following Table addresses the 1st Revision comments from Reviewer 5.

1	Much of the process modelling appears to draw from previous works, although some of the cited references in this regard do not seem directly relevant to the topic: these should be reviewed carefully.<br><br>In any case, whenever new system features are introduced, as appears to be the case here, the process description should always be clear to the reader, and all assumptions should be made explicit for reproducibility purposes. See the detailed suggestions in the subsequent comments.<br><br>The authors compare their novel rSOC concept against a benchmark / conventional rSOC concept, both in the main text and in the supplementary material. The authors should present the layout scheme of the reference concept, so that the	Thank you for your valuable comments and suggestions.<br><br>We fully agree that the reference case (or benchmark design) was not clearly stated in the main manuscript and that the connection with the SI was not sufficiently clear. In the initial version of the manuscript, we intentionally placed most of the process-related methodology and detailed process results in the SI, as we did not want to overly emphasize the process design advancement in the main text. However, we realized that this approach could make it difficult for readers to understand the reference case and the basis of comparison. During the previous revision stage, we therefore moved and summarized several process-related descriptions from the SI to the main manuscript while retaining the detailed information in the SI due to the concern on the text length limitations.<br><br>Based on your suggestion, in this revision, we further revised the manuscript to better highlight the novelty of the proposed system and to clearly describe the conventional reference designs used for comparison. Specifically, we have added a more detailed explanation of the proposed SOEC and SOFC system designs in the Method section, clarified the limitations of conventional configurations, and explained where and why the proposed improvements were introduced. In addition, the assumptions of conventional designs and their references are now explicitly described in the Methods section.

	introduced novelties are made clearer to the reader.<br><br>The CAPEX comment has been resolved. However, it is not clear to me what the reference capacity for the rSOC CAPEX is. Does the value of 10 k€/kW refer to the SOFC or to the SOEC capacity? Given the 3:1 power ratio between the two modes, this distinction has a significant impact on the result.	In the revised Results section, we also present a clearer comparison between the proposed and conventional designs, including explanations linking the observed improvements to the corresponding design modifications described in the Methods section. While the detailed modelling inputs remain in the SI Methods section and the complete mass and energy flow data are reported in the SI Results section, the main manuscript now contains sufficient information for readers to understand both the reference case and the novelty of the proposed system without relying solely on the SI.<br><br>The main revisions can be found at:<br>• SOEC results: pages 5-6, lines 107-122<br>• SOFC results: pages 6-7, lines 144-150 and lines 154-156<br>• Detailed description of the rSOC system design and conventional configurations: pages 27-29, lines 644-683 and pages 29-31, lines 695-739<br><br>Regarding the references, we carefully reviewed and updated the reference list. Several references were corrected, and additional relevant references were included. Please see page 28, References 40-42; page 29, References 43-45; Page 3-4, References 14-20 and References 21-22.<br><br>Finally, thank you for your comment regarding the (rSOC) reference cost. The legend labels have been revised to clarify that the CAPEX is defined per unit of produced power (kW) in SOFC mode. The labels have therefore been updated to kW_{FC} (fuel cell) to avoid ambiguity. Please see page 21, line 488 and Figure 3(e).
2	It is also true that the optimization results rely heavily on some of the process modelling outcomes, so it is appropriate to dedicate some space to this in the main paper.<br><br>That said, in the current version, the presentation is, in my opinion, overly descriptive and does not convey strong, clear messages. The text contains numerous numbers relating to component power consumption, heat exchanger areas, and so on, which are difficult to digest without a reference point or broader context.	Thank you for your thoughtful suggestions. We agree with you.<br><br>As stated in our reply to Comment 1, in this revision, the benchmark process for the rSOC system is now clearly defined in the Methods section. A detailed description of the benchmark system designs and the proposed system design have been added to the Methods section. Please refer to the sections "Advanced SOEC System Design and Modelling" and "Advanced SOFC System Design and Modelling" on pages 27-30.<br><br>In the Results section, we explicitly link the improvements of the proposed system, such as efficiency gains and reductions in utility consumption, to the corresponding process design modifications. Please see the Results section on pages 5-6 (lines 107-122) and pages 6-7 (lines 144-150 and 154-156).

	In the first results section, I would recommend focusing more on relative improvements with respect to the benchmark (e.g. percentage increases or decreases), explaining the main drivers behind these improvements, how they impact system efficiency, and how they relate to the distinguishing features of the novel system concept.<br><br>Finally, while I understand that the two parts of the paper are connected, this link is not entirely clear to me. Specifically, how is the information on rSOC performance used in the optimization part? Is roundtrip efficiency used as a key input for optimization? And given the inherently flexible nature of the rSOC system, how are part-load performances accounted for? I would ask the authors to better clarify the exchange of information between the two parts of the paper. If part-load performances have not been estimated, the reasons for this should be discussed, along with the potential impact on the results of the analysis.	Moreover, thank you for highlighting the potential confusion regarding the link between the modelling and optimization parts of the study. We agree that this connection should be made clearer. In the revised manuscript, we now explicitly explain how the modelling outcomes are used as inputs for the system integration and optimization analysis. Specifically, we clarified which key parameters derived from the process model are used in the system-level evaluation. This clarification has been added in lines 205-214 on page 9 of the revised manuscript.<br><br>Regarding part-load operation, this aspect is not included in the present study because the rSOC system is assumed to operate either at full load in one mode (SOEC or SOFC) or to remain in hot standby when grid conditions are not favorable for operation. This assumption reflects the relatively small scale of the rSOC system compared with national or regional energy demand, where system operation is primarily determined by the economic trigger point between SOEC and SOFC modes under current grid conditions. This assumption is now clarified in the revised manuscript (lines 208-210 on page 9).<br><br>Nevertheless, we acknowledge that dynamic operation and part-load performance are important aspects that could further improve system analysis. Therefore, we have added this point as a direction for future work in the revised manuscript (lines 606-607 on page 25).
9	Line 49, other projects have also addressed and tested rSOC technology, please extend your review.<br><br>Not addressed completely. A brief mention of other relevant projects, as suggested by the original Reviewer #4, would be expected.	Thank you for your comment.<br><br>In fact, we conducted a detailed literature review prior to this study to ensure that our work does not duplicate existing research. This review is presented in the Supplementary Information, Method S0. However, we agree that relevant studies should also be briefly mentioned in the main manuscript. Therefore, in this revision, we have added a concise summary and additional references in the Introduction. Please see page 3, lines 56-62.<br><br>Furthermore, several additional references have been included in the revised manuscript (e.g., References 14-20, References 21-22 on page 3-

		4).
13	The definition of "conversion efficiency" at line 77-78 is unclear (e.g., is that electricity-to-LHV?).<br><br>For better clarity and reading flow, a short description of what conversion efficiency encompasses should be added in the main Results section when first introducing the related results.	Thank you for your suggestion.<br><br>We feel that Methods section would be better to define the conversion efficiencies. For the SOEC system, the conversion efficiency is defined as the ratio between the chemical energy content of the produced methane and the total electrical power input to the system. The corresponding mathematical expression has been included in the Methods section. Please see page 29, lines 685-693 of the revised manuscript.<br><br>For the SOFC system, the electrical efficiency is defined as the ratio of the net electrical power output to the chemical energy input provided by the methane fuel. Please see page 30-31, lines 730-739 of the revised manuscript.
14	In Figures A1 and A2 in SI, electricity seems to be transferred from the grid to the rSOC stack. How is power electronics considered? This deserves some discussion.<br><br>However, I do not agree on the “relatively minor impact on overall system efficiency” point, as a loss of 5% in one direction of the conversion is magnified when computing roundtrip loss (up to 9-10% impact). Therefore, due to the relevance of this, this should be made more explicit. For example, this information can be added when describing the conversion efficiency (see comment #13). In the SI, it can be made more explicit when detailing SOFC/SOEC mode results in the text, and in Tables B1 and B2, by adding proper subscripts (such as kW_DC).<br><br>More importantly, the impact of adding power electronics conversion should be discussed when presenting	Thank you for comment.<br><br>The impact of power electronics and grid components on the round-trip efficiency, and hence on the storage requirements and LCOS, is indeed not negligible and has been assessed in the revised manuscript. For this analysis, as recommended by the reviewer, we considered 2 extreme countries, Switzerland (long-term oriented) and Estonia (short-term oriented) to limit excessive changes at this stage of the revision, while ensuring coherent information. The main findings can be found in the SI Figure B11, where a bi-directional transformer efficiency of 97.5% has been emphasized through a dotted line.<br><br>Moreover, a brief discussion on this aspect has been introduced in the revised manuscript, page 8, lines 187-189; page 22, lines 516-524.

	the results, by performing additional calculations, and compare it with main results, which do not include this relevant loss. To avoid excessive additional work at this stage of the revision, this can be done for two extreme cases (two locations of choice).	
15	Line 81, it is not clear what the "cold utility demand" refers to.<br><br>Addressed, but the purpose of the cooling water should be specified.	Thank you for the suggestion.<br><br>The cooling water is used to remove the remaining excess heat in the rSOC system after internal heat recovery. This clarification has been added to the revised manuscript. Please see page 5, lines 118-119.
16	Information on the electrochemical assumptions is essential (thermoneutral voltage? ASR taken from where? 0D or 1D modelling?)<br><br>I could not find any mention of the description of the 0D model, neither in the main paper nor the SI. The authors only briefly mention the Aspen Plus model for the system. However, no details are given about how the rSOC cell/stack is modelled. The authors say in the response to this comment that they developed the rSOC model for this study, so some description of how it is done is required in this study. The authors are encouraged to expand on this and provide more electrochemical data, such as the ASR raised by original Reviewer #4. Within the detailed results of the SI, notably, there is no instance of operating voltage in both modes, which is a key parameter to understand performance. A figure in the SI showing validation of polarization curves model against experimental data could be of aid.	Thank you for your detailed comment.<br><br>In this study, we did not conduct experimental validation of the model, as the experimental testing and validation of the rSOC system have already been carried out by our collaborators in previous study. Since these experiments are not part of the work presented in this paper, we did not include experimental validation results in the SI.<br><br>However, we agree that it is important to demonstrate the reliability of the modelling approach. Therefore, we have cited the study in which modelling and experimental validation of rSOC system was performed, and readers can refer to that work for the detailed validation results (see page 29, Reference 43).<br><br>In addition, to improve the transparency of the modelling framework, we have included several key model inputs and nominal operating conditions in the revised manuscript. These additions can be found on page 28, line 669, and page 29, line 698, together with the corresponding references. Furthermore, the ASR values used in the model are now reported in Table A2 in the SI.

17	The rationale behind the (reasonable) choice of the ratio 1:3 for SOFC and SOEC nominal power is not expressed.<br><br>As suggested in comment #16, nominal operating voltages for both modes should be provided.	Thank you for your question.<br><br>As stated in our response to the previous comment, we have now included the operating voltages for both SOEC and SOFC modes in the revised manuscript. We have also cited the SolydEra stack handbook, where readers can find additional technical details regarding the stack specifications and operating conditions (see page 28, References 40-42).
20	Fonts in Figure 1 are too small.	Thank you for your observation.<br><br>The font size has been increased for Figure 1.
23	Line 162 mentions "CO2 increase". In general, the benchmark of the overall analysis is not clear.<br><br>The benchmark is still not clear to me. What does "no further use of electricity" mean?.<br><br>I strongly suggest the authors to declare clearly the benchmark when they introduce the analysis. In particular, the benchmark is still not clear for results in Figure 1b and 1c.	Thank you for your valuable comment.<br><br>In the revised manuscript, we have clarified the definition of the reference case in the first paragraph of the "integrating long-term storage system into the current energy framework". Specifically, the reference case represents the current energy system without rSOC deployment; therefore, no additional environmental or economic benefits are realized. This scenario is used as the reference case for evaluating the impacts of rSOC integration under present grid conditions. We also clarified that this reference case applies only to the current energy framework considered in this study. In future energy systems, where higher levels of renewable integration and energy storage are expected, the rSOC system's objective shift toward self-sufficiency rather than purely environmental or economic benefits.<br><br>The corresponding clarification has been added to the revised manuscript (page 9, lines 208-212).
24	Lines 160-174 are strictly descriptive; there is no clear message.<br><br>I have one suggestion to improve clarity: rather than focusing solely on absolute values, the authors can emphasize this by adding % of OPEX increase vs. CO2 emissions decrease. In their discussion of case G vs. J, for example, decreasing 7% CO2 emissions require 20% OPEX increase.	Thank you for the comment.<br><br>In this analysis, we have presented trade-off solutions between CO_2 emissions and operational costs. We could not consider any of the Pareto solutions as a basis for comparison as all Pareto solutions are equally good mathematically, but we have compared them for implementation purposes.<br><br>Please note that we have followed a standard approach to present and discuss tradeoff solutions. Please see Figure 1(c), Trade-off solutions analysis section, and Table B5 in the SI for detailed analysis of Pareto solutions.

26	Lines 232-234: is the ratio computed on solar+wind or including all RES (including geothermal and hydro)? What about biomass? referred to which year?<br><br>This should be specified in the main text as well.	Thank you for the suggestion.<br><br>This is clarified in the revised manuscript on page 15, lines 362-363, where it is specified that the ratios are calculated based on the total renewable energy production in the 2050 future scenario.
27	The "hybrid tank for fuel and CO2 storage" is never really presented. How does it work? How does it separate the fluids?<br><br>A brief introduction to the concept of the hybrid tank should be provided before presenting the related results, for improved clarity and flow. For further details, readers can still be referred to the Methods section.	Thank you for your suggestion.<br><br>In the revised manuscript, we have included a few sentences on the hybrid tank before presenting the related results.<br><br>Please see lines 310-313, on page 13 (Hybrid tank performance).
**Methods**		
31	The use of electricity for the entire preheating (incl. evaporation) of water before the SOEC stack makes it hard to believe the high conversion efficiency obtained, without thermal recovery from cathode outlet.<br><br>It is still very difficult to me to understand the process, especially for the SOEC system. The layout is too simplistic and does not show neither all flows nor all heat interactions. I can understand that some of the steam feed is produced via heat recovery from methanation, but I could not find any description of this in the text. The authors only write in Methods section: "heating water to 750°C through sequential heating, evaporation, and superheating (H'1) before entering	Thank you for your comments.<br><br>In the manuscript, Figure 4 presents proposed SOEC and SOFC system configurations. In the SI, Figure A1 presents conventional SOEC system configuration, whereas Figure A3 presents three conventional SOFC system configurations: no AOG (anode offgas recirculation), warm AOG and hot AOG. In the SI, Tables A1 and A2 present key parameters used in the modelling of SOEC and SOFC systems.<br><br>In the SI, Tables B1 and B3 presents details of material and energy flows for the proposed and traditional SOEC and SOFC systems. In all system configurations, heaters and coolers are used to supply and remove heat. In the SI, Table B2 and B4 show duties and temperatures for different streams in the proposed and traditional SOEC and SOFC configurations.<br><br>Based on your comment, we rectified a few typos in Tables B2 and B4. In Figures 4, A1 and A3, some heaters and coolers have phase changes: H'1, C'1 and C'3 (proposed SOEC in Figure 4a, and traditional SOEC in Figure A1), C2 (proposed SOFC in Figure 4b, and traditional SOFC in Figure A3), and H2 (No AOG SOFC design in Figure A3). Further, three-stage methane fuel expansion has three heaters (H1 in Figure A3)

	the cathode side of the stack". And information about this is even scarcer in the SI. For example, what happens to water from 25°C to 750°C? Tables B2 for example mentions some streams that are not present in the presented layout (for example, what is H'3?).<br><br>I strongly suggest the authors to provide more detailed process layouts and descriptions. I see that there are many heat exchangers and the process integration is complex, but at least the readers should be able to understand the following key points:<br><br>1- What are the main heat transfer interactions within the process?<br>2- If electric heaters are used to complete evaporation and superheating in SOEC mode, to what extent?<br>3- What is the amount of heat that is left available for downstream processes? At what temperature and pressure?	before three turbines, whereas three-stage CO_2 compression has three coolers (C3 in Figure 4b and A3) after three compressors. In Tables B2 and B4 duties and temperatures are presented for all heaters and coolers, including all specific information on phase changes.<br><br>In the revised SI, we have included two paragraphs (pages 11 and 15) on different hot and cold streams, explaining phase changes and heaters/coolers for multi-stage expansion/compression. We believe the information provided on modelling parameters, material and energy flows, and temperature levels is enough for reproducibility of the results.<br><br>Heat exchange between hot and cold streams is crucial to reduce the demand for hot and cold utilities. In this study, we have performed heat cascade analysis, and corresponding results (including hot and cold utilities) are already presented in Tables B1 and B3. In case of SOEC, 0.363 kW heat must be supplied at high temperatures, and we have considered an electric heater for this purpose (Table B1). In case of SOFC, no external heat source is required (Table B3).<br><br>For the proposed SOFC system, all streams must cool down, except H1 (Table B4 in the SI, Proposed SOFC). Both SOEC and SOFC systems have some waste heat available at varying temperatures, which can be visualized using integrated composite curves (ICC) for SOEC and SOFC systems (Figure B2 in the SI). The waste heat can be valorized by integrating a Rankine cycle (RC). Figure B2 also presents ICC for SOEC and SOFC systems with RC. Finally, revised Figure B2(a) shows methanation, water evaporation and electric heater for the SOEC system.
32	The valorization of waste heat from the rSOC to drive a Rankine cycle is hard to believe, since available temperature of the streams after thermal integration for the rSOC itself is below 300°C. Needs more info and support.<br><br>Partly related to my answer to comment #31: I see that TQ composite curves have been added, but they are really confusing and unclear to me. Authors should add more descriptions to the plots, highlighting major streams,	Thank you for your comments.<br><br>In the revised SI, we have included some information on integrated composite curves (ICC), see pages 11 and 15. We chose to present ICC instead of traditional hot and cold composite curves, as ICC present valuable information for interested readers on valorization of waste heat via Rankine cycle. As stated in our response to Comment 31, we have shown major process units (methanation, water evaporation and electric heater) on the heat curves.

	contributions of utilities and other components if present (e.g., in the plot, where is the contribution of the the methanator?)	
34	Line 533, 10% unconverted fuel means 90% utilization factor, which is extremely high. How is that possible?<br><br>It should be clarified whether it refers to the global utilization factor or to the per-pass utilization within the stack — if the latter, the concern stands.<br><br>Furthermore, references 12, 31, 43, 44 do not appear to be directly linked to SolydEra. If specifications were provided by SolydEra, this should be stated explicitly in the text.	Thank you for your question.<br><br>In this study, we did not report single-pass or global fuel utilization because we observed that different studies define and calculate these metrics in different ways, which can make direct comparison unclear. However, according to the SolydEra stack specifications, the stack can accommodate up to 90% internal methane reforming. This value has been confirmed by the stack manufacturer, and accordingly, we have cited the SolydEra stack documentation. Please see page 28, References 40-42.<br><br>Regarding the references, we carefully reviewed and updated the reference list. Several references were corrected, and additional relevant references were included. Please see page 29, References 43-45.
35	Overall, it seems that the rSOC modelling is very simple / approximate. If that cannot be improved, make sure to highlight the assumptions and limitations.<br><br>I think there is something off with some the cited references (see also comment #34). The authors cite Ref. 25 when talking about modelling and validation of the SOEC BoP model, but Ref. 25 is titled "Effect of oxygen partial pressure on the electrochemical impedance of La0.8Sr0.2MnO3−δ/Zr0.92Y0.08O2 porous composite anodes in solid oxide electrolysis cell" and seems related to experiments at cell level. This and other references should be checked carefully.	Thank you for your comment.<br><br>We carefully reviewed and updated the reference list. As stated above, several references were corrected, and additional relevant references have been added to the revised manuscript. Please see page 29, Reference 43 for the model validation part.

New comments		
39	I think that the symbols in Figure 13 the (triangle and diamond) are inverted for 1k and 10k rSOC CAPEX.	Thank you for your observation.<br><br>We have corrected this typo in the revised manuscript. Please see page 19, Figure 3(e).
40	In the last version of the paper, the authors have introduced the concept of levelized cost of storage (LCOS) as an important KPI to better capture the potential benefits of using rSOC systems for long term storage. They provide details about how it is computed in the supplementary information, both for single technology and hybrid use of battery and rSOC. The authors should briefly describe how it is computed also in the main text, right before introducing the results. This must be done especially for the hybrid case, where some assumptions about rSOC and battery capacities are made.<br>Moreover, I noticed that they include only CAPEX. Shouldn't it include also operation and maintenance costs? Usually these costs are in the range of 2-3% of the total CAPEX per year, lower than the annual fraction of CAPEX, but I would not say negligible.	Thank you for your comment.<br><br>We agree that the CAPEX and LCOS calculations should be presented in the main manuscript. Accordingly, we have added a dedicated section at the end of the Methods section describing the CAPEX and LCOS calculations. Please see page 37, lines 880-893, in the revised manuscript.<br><br>Regarding your second comment, we have updated the LCOS calculations to account for maintenance costs, assumed to be 2% of the initial capital expenditure. This update slightly modifies the results. During this revision, we also improved the CAPEX calculations by accounting for the time value of money in future investments using the interest rate (*i*). Further, methodological details can be found in the SI (Method S4).<br><br>For the impact of maintenance costs on the results, please see page 19, Figures 3(d) and 3(e), which have been updated accordingly. Tables B9 and B10 in the SI have also been revised.
